# Supplementary material for: Manganese is a physiologically relevant TORC1 activator in yeast and mammals
Source: eLife. 2022 Jul 29;11:e80497. doi: 10.7554/eLife.80497 (PMC9337852; doi:10.7554/eLife.80497)

Figure 1E
Data shown in Figure 1E correspond to Expt2.

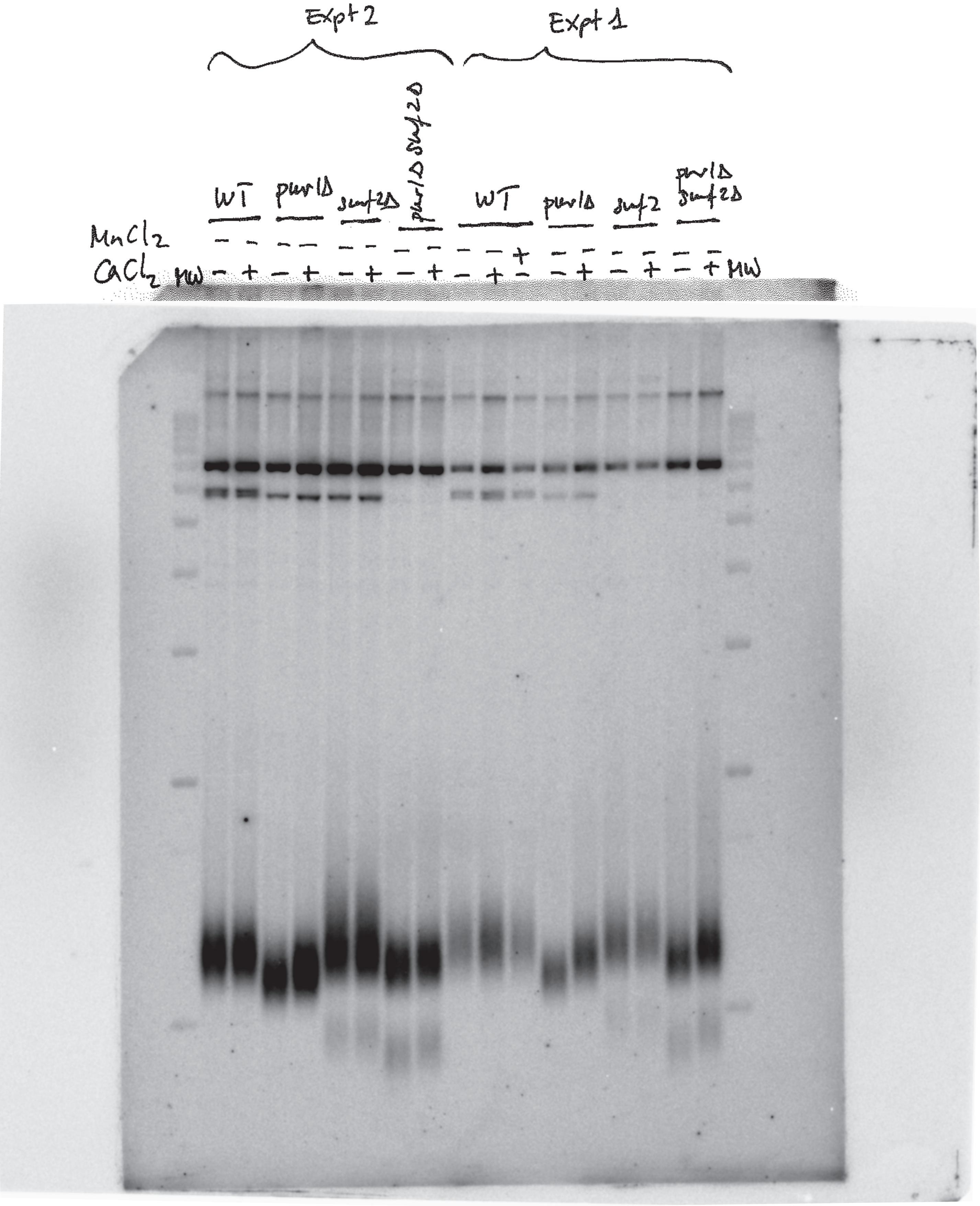

Supplement: Figure 1—source data 1. [file elife-80497-fig1-data1.pdf]
