# Supplementary figures and images for: Manganese is a physiologically relevant TORC1 activator in yeast and mammals
Source: eLife. 2022 Jul 29;11:e80497. doi: 10.7554/eLife.80497 (PMC9337852; doi:10.7554/eLife.80497)

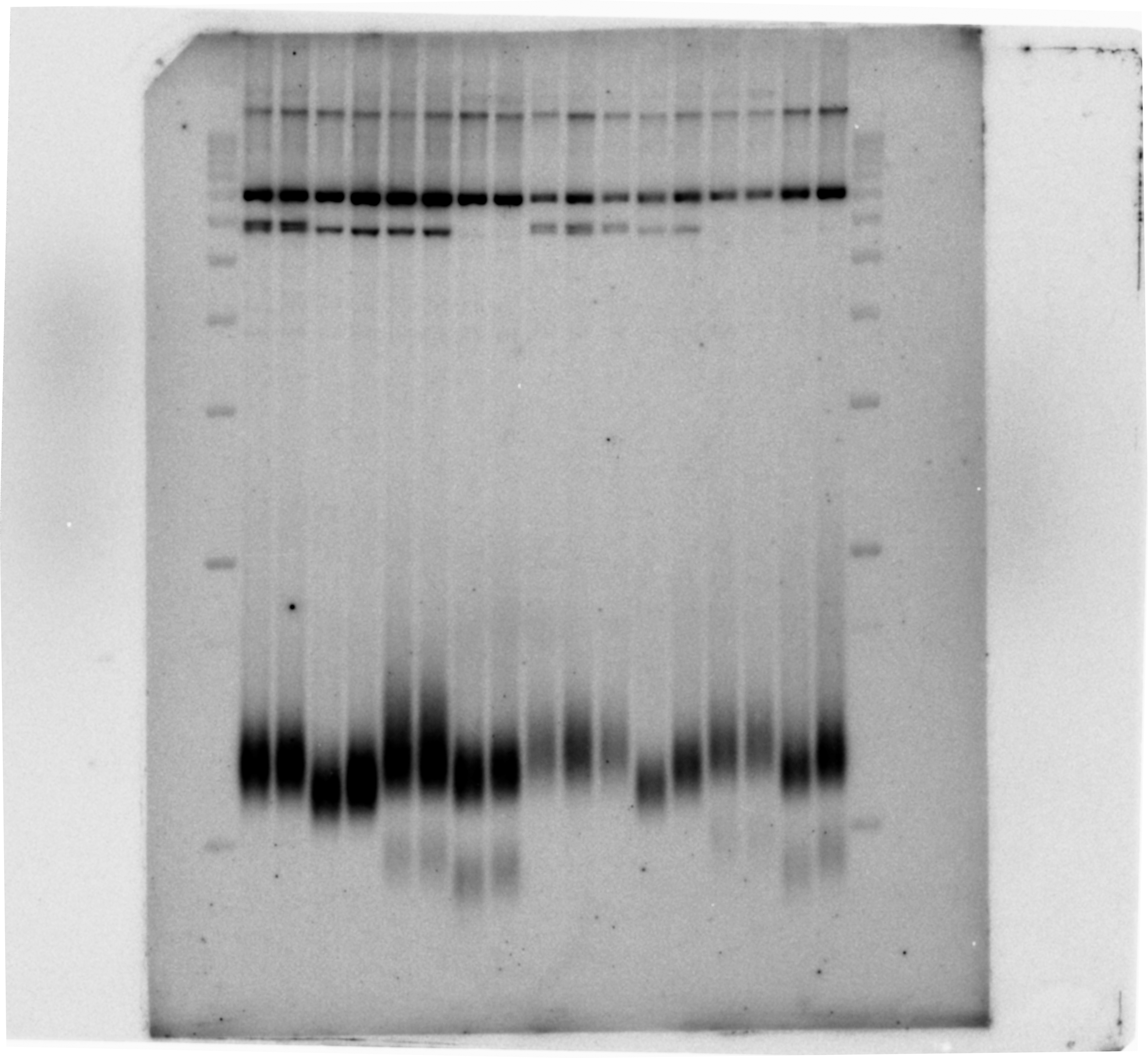

Supplement: Figure 1—source data 2. [file elife-80497-fig1-data2.zip › Figure 1-source data 2.tif]

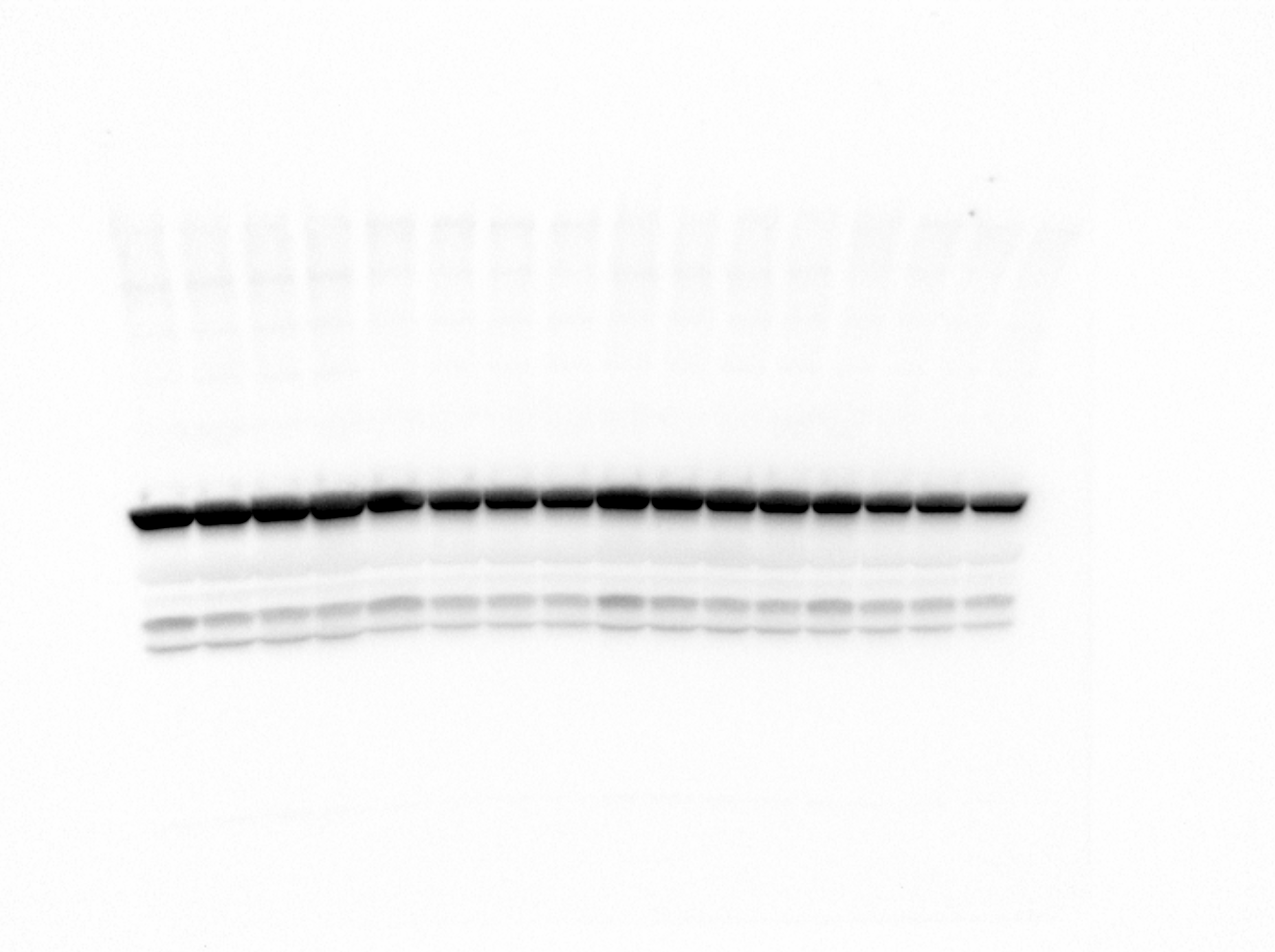

Supplement: Figure 1—figure supplement 1—source data 2. [file elife-80497-fig1-figsupp1-data2.zip › Figure 1-figure supplement 1-source data 2/G6PDH.tif]

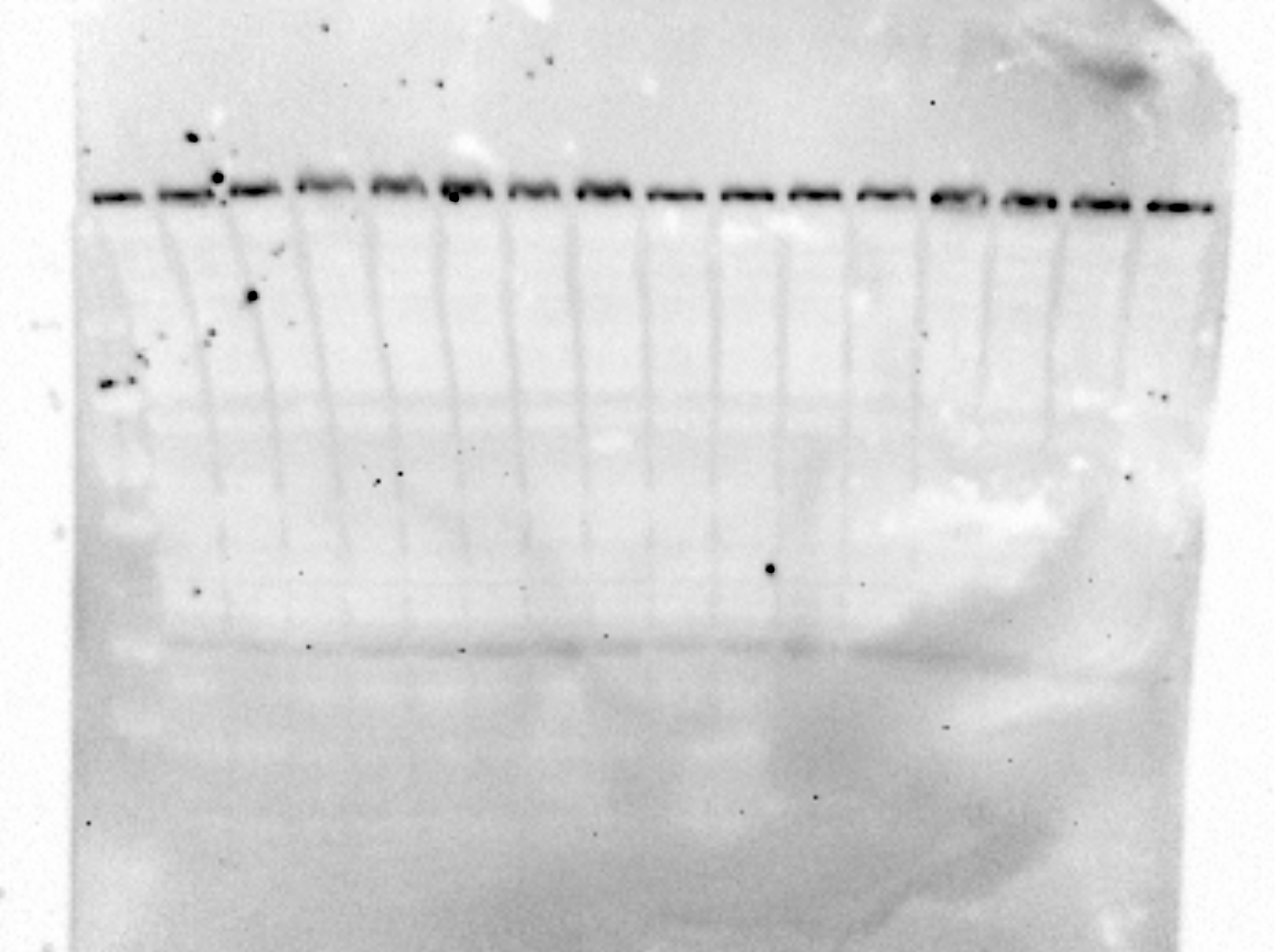

Supplement: Figure 1—figure supplement 1—source data 2. [file elife-80497-fig1-figsupp1-data2.zip › Figure 1-figure supplement 1-source data 2/GFP-Tor1.tif]

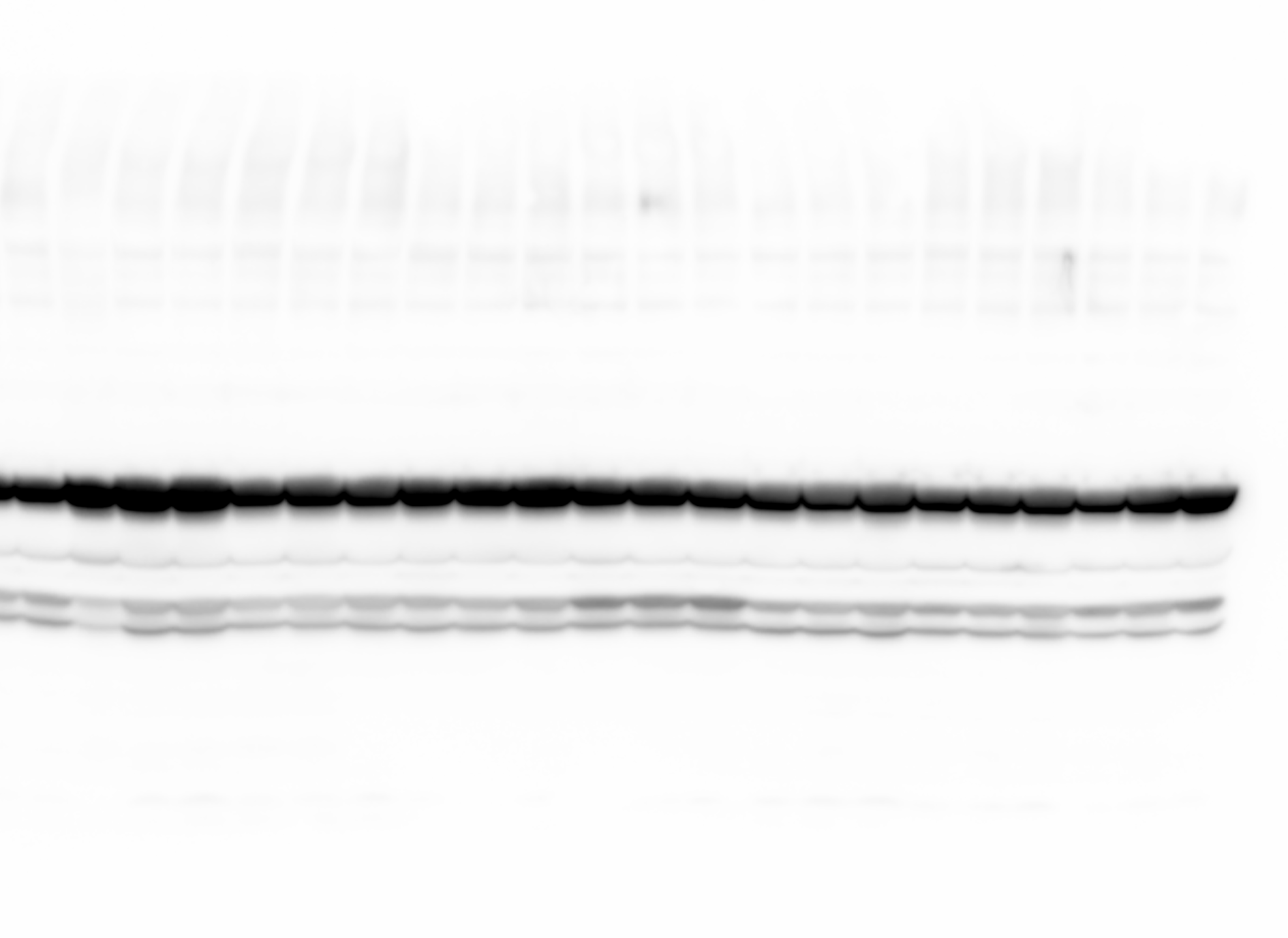

Supplement: Figure 2—source data 3. [file elife-80497-fig2-data3.zip › Figure 2-source data 3/Figure 2F/rtg3_Expts1&2_right_G6PDH.tif]

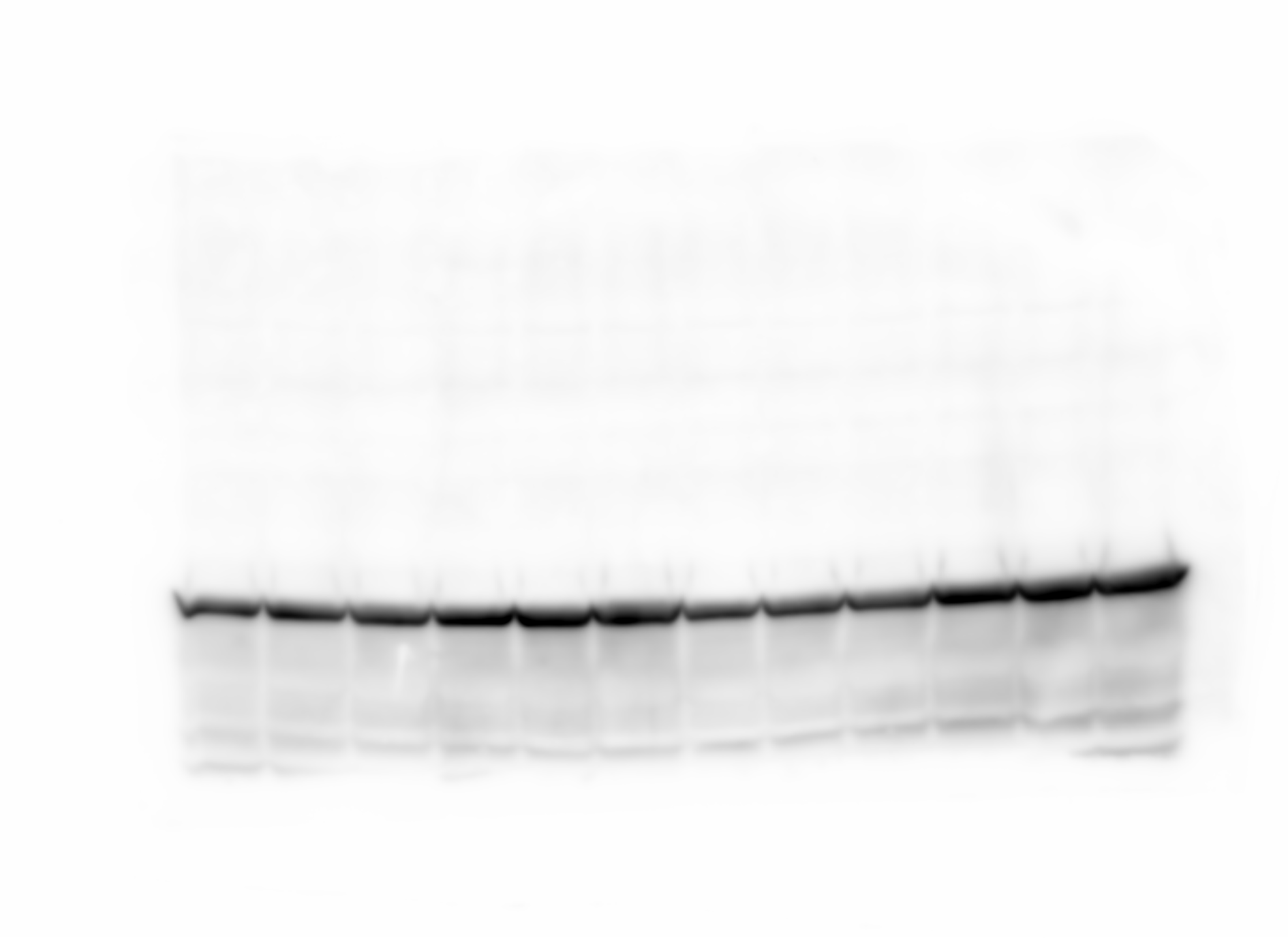

Supplement: Figure 2—source data 3. [file elife-80497-fig2-data3.zip › Figure 2-source data 3/Figure 2F/rtg3_Expt4_G6PDH.tif]

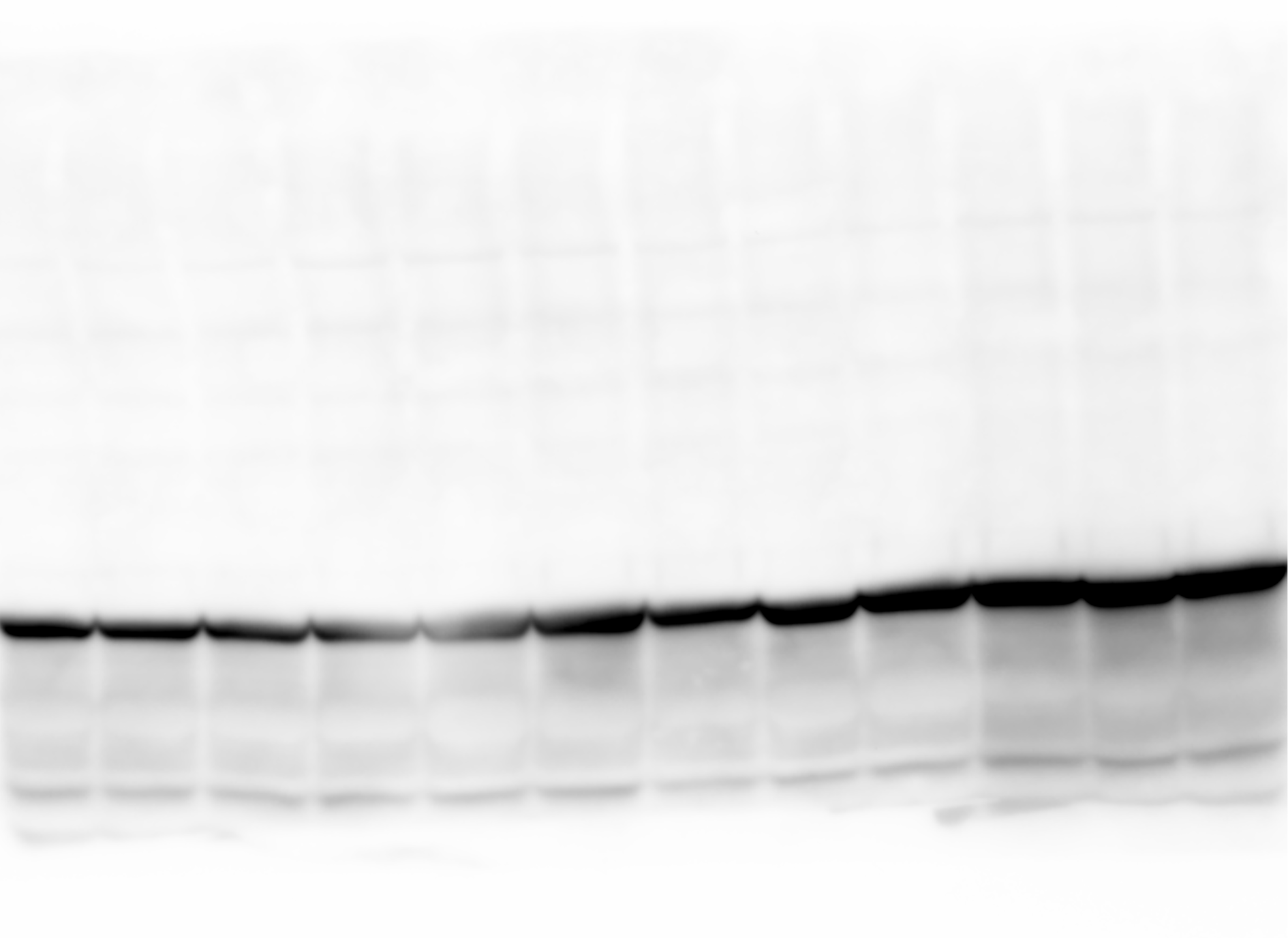

Supplement: Figure 2—source data 3. [file elife-80497-fig2-data3.zip › Figure 2-source data 3/Figure 2F/rtg3_Expt3_G6PDH.tif]

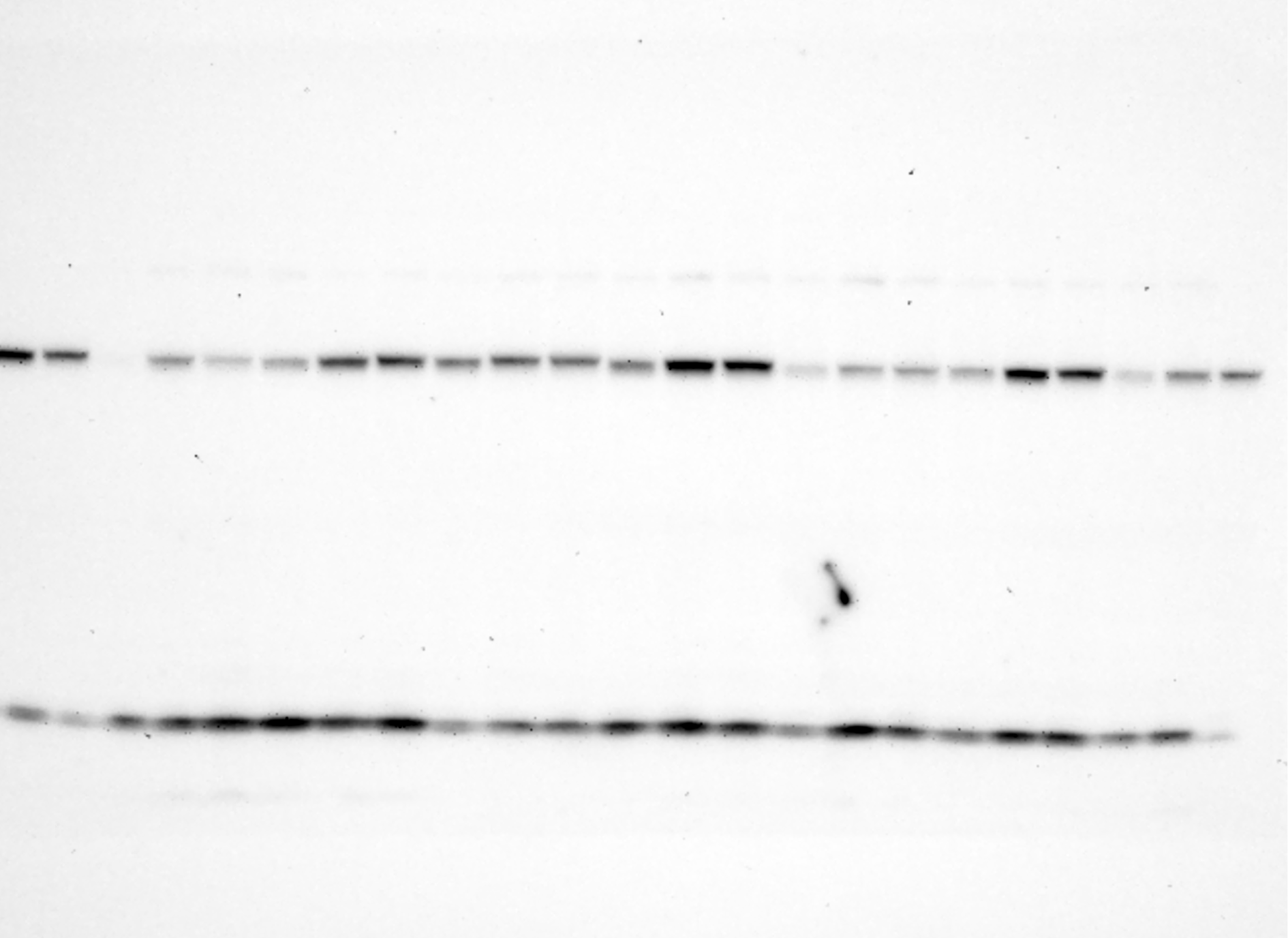

Supplement: Figure 2—source data 3. [file elife-80497-fig2-data3.zip › Figure 2-source data 3/Figure 2F/rtg3_Expts1&2_right.tif]

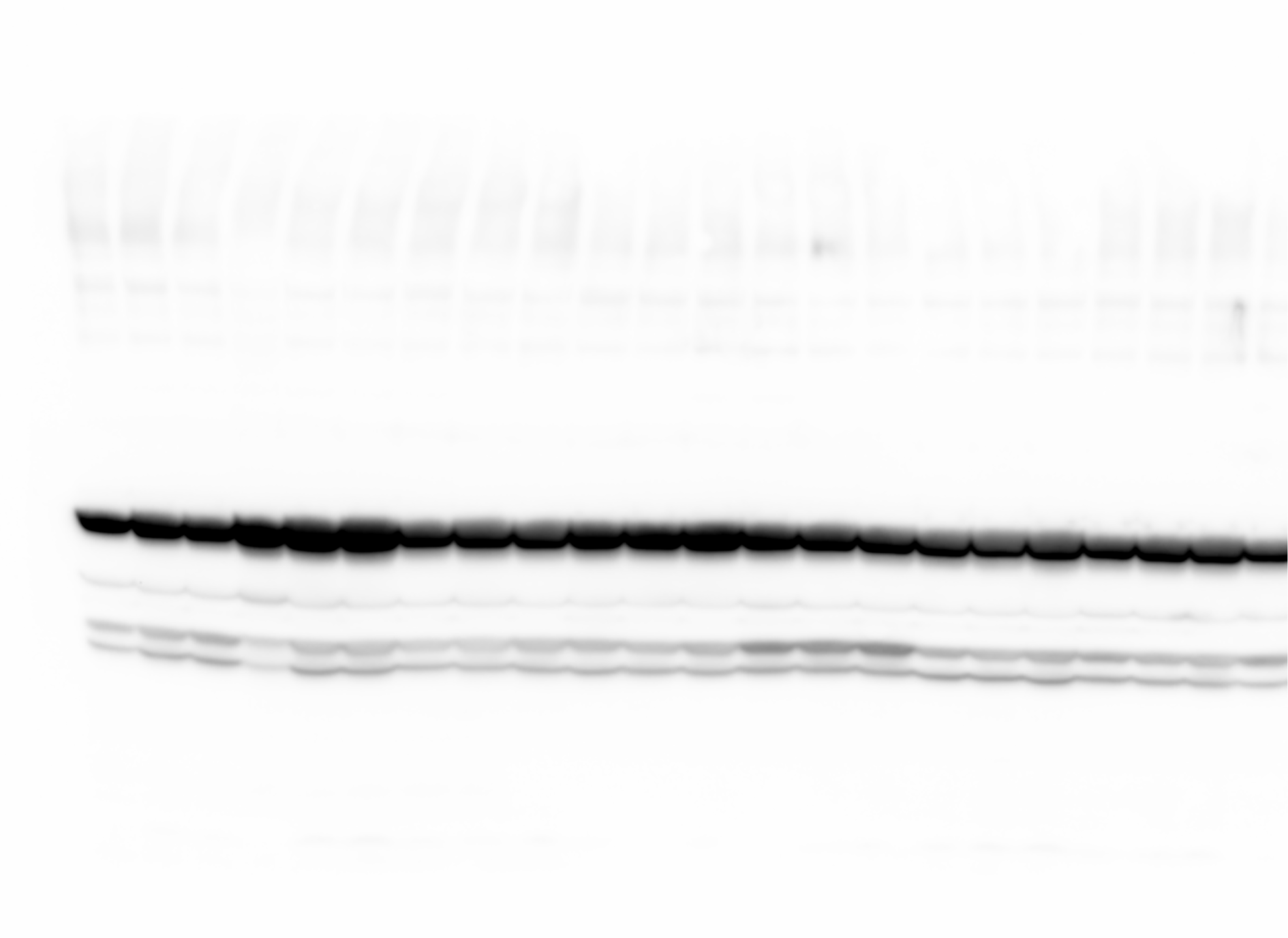

Supplement: Figure 2—source data 3. [file elife-80497-fig2-data3.zip › Figure 2-source data 3/Figure 2F/rtg3_Expts1&2_left_G6PDH.tif]

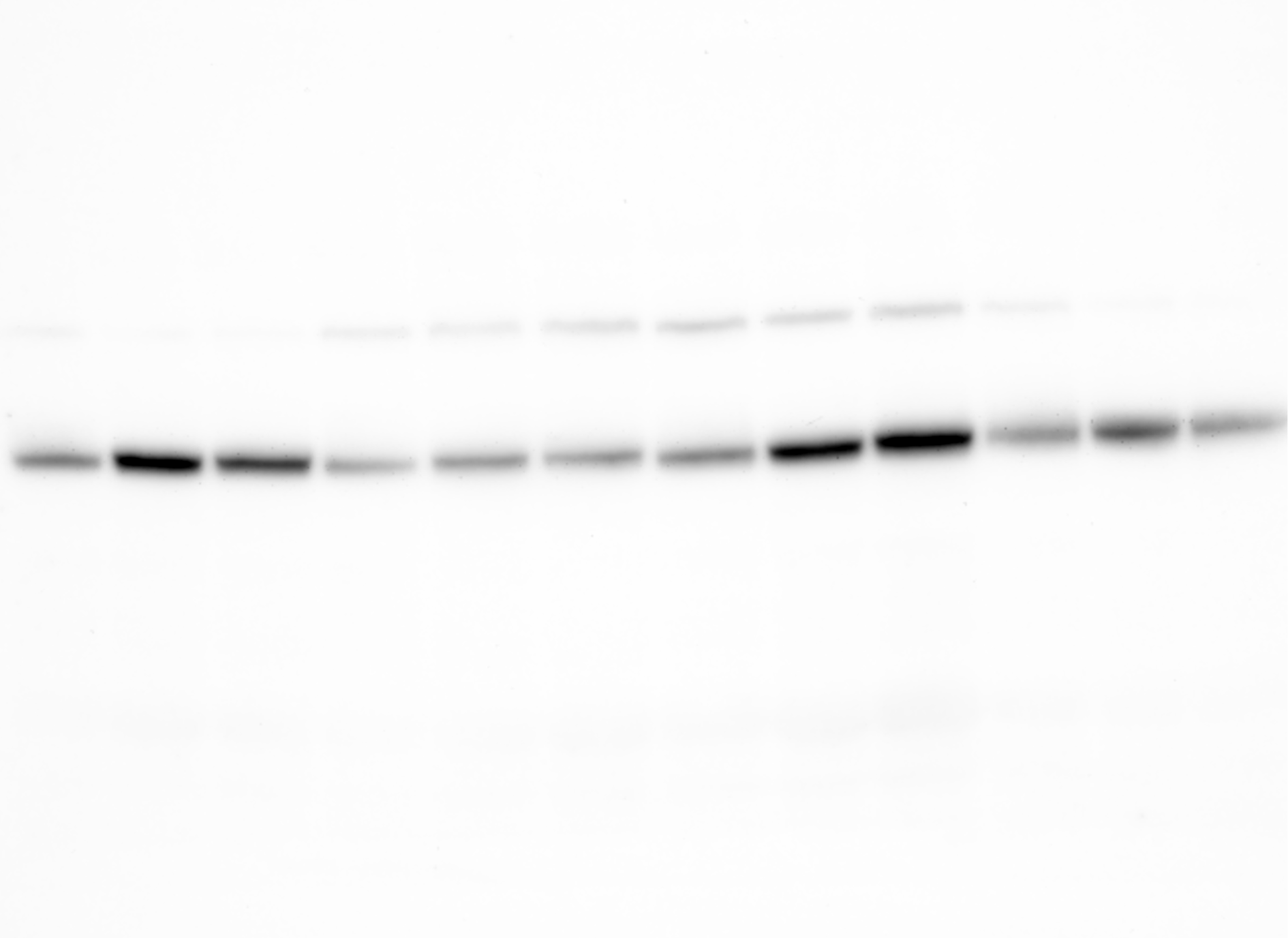

Supplement: Figure 2—source data 3. [file elife-80497-fig2-data3.zip › Figure 2-source data 3/Figure 2F/rtg3_Expt3.tif]

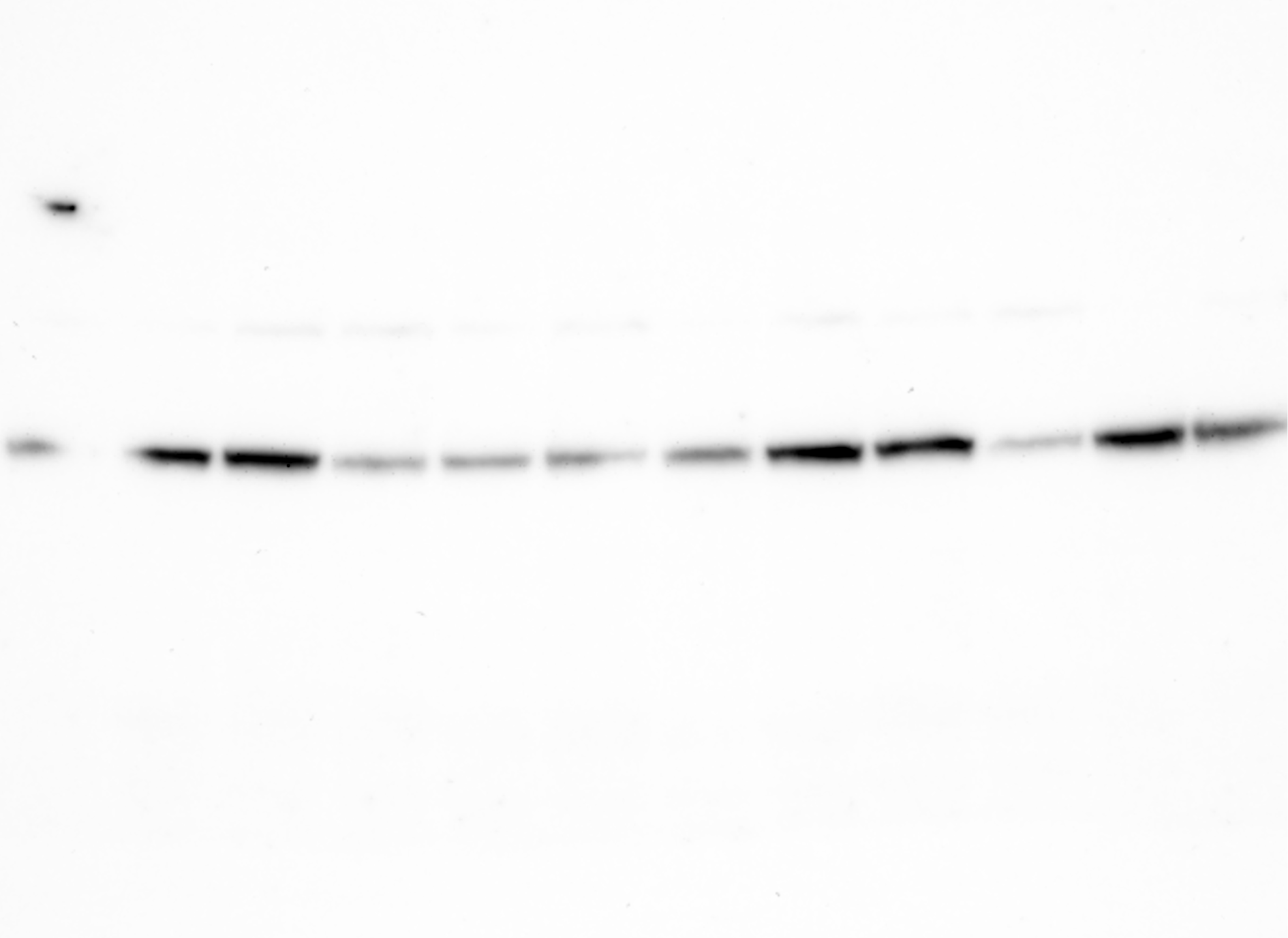

Supplement: Figure 2—source data 3. [file elife-80497-fig2-data3.zip › Figure 2-source data 3/Figure 2F/rtg3_Expt4.tif]

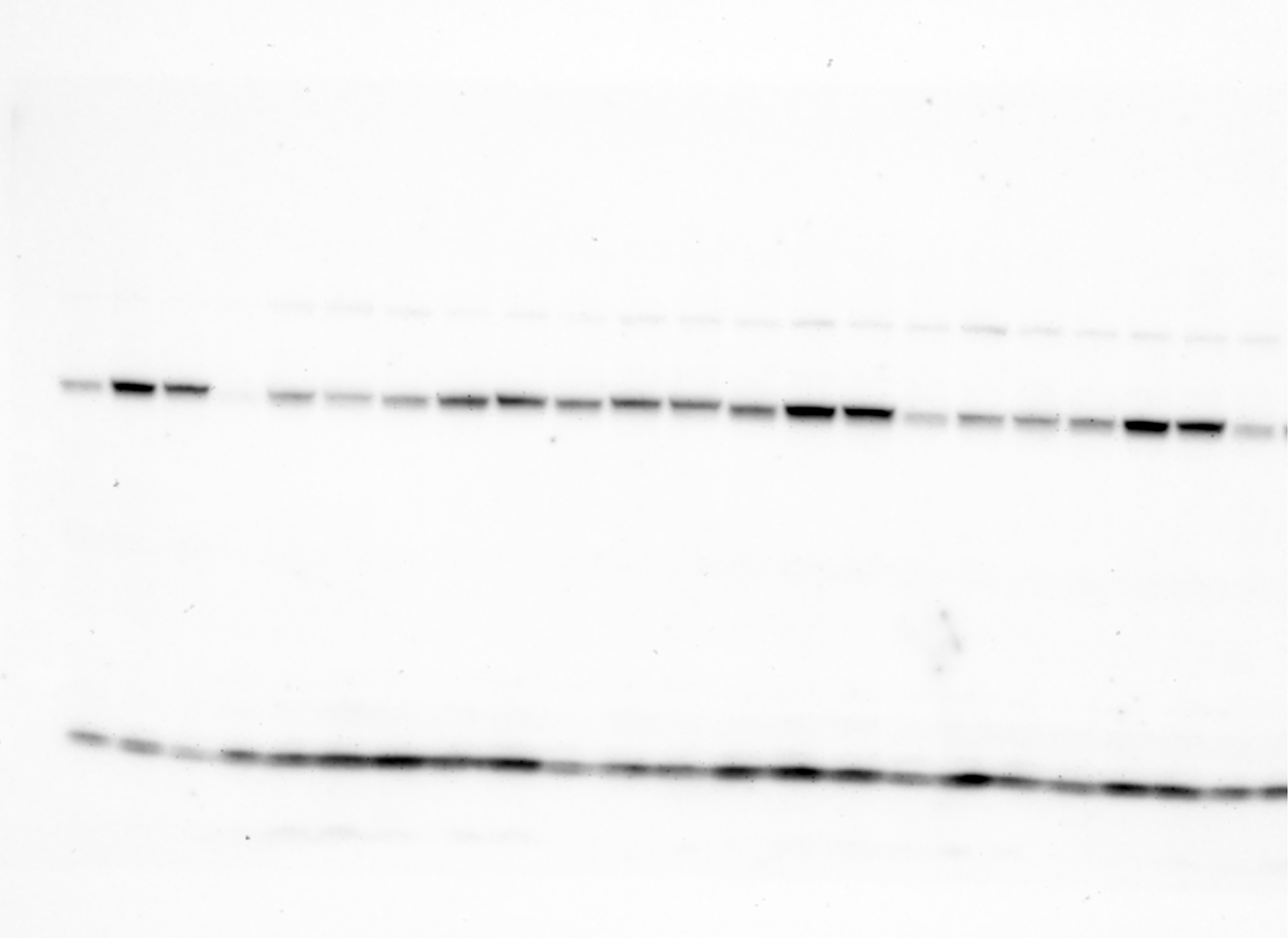

Supplement: Figure 2—source data 3. [file elife-80497-fig2-data3.zip › Figure 2-source data 3/Figure 2F/rtg3_Expts1&2_left.tif]

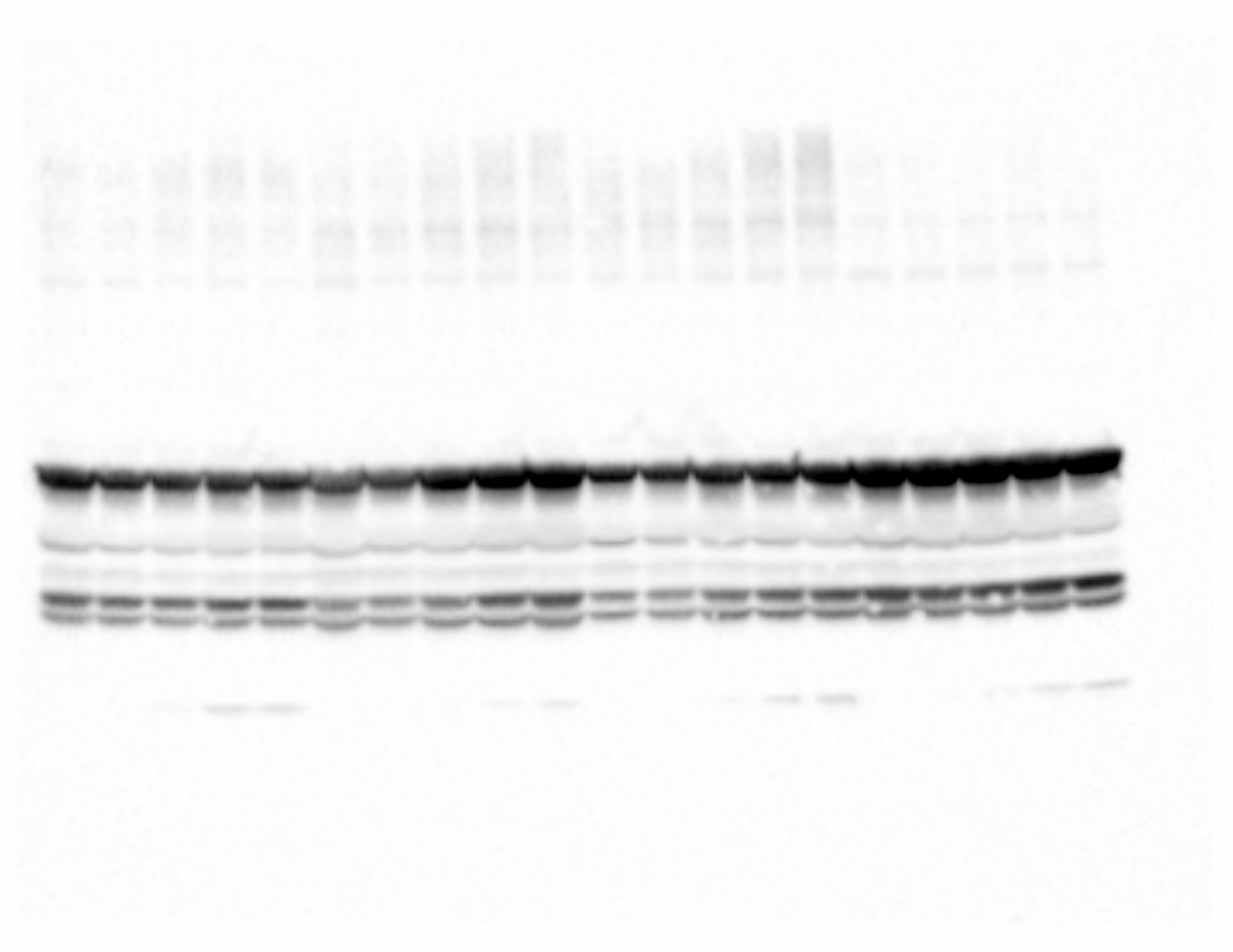

Supplement: Figure 2—source data 3. [file elife-80497-fig2-data3.zip › Figure 2-source data 3/Figure 2A and 2C/Atg8_Expt2_G6PDH.tif]

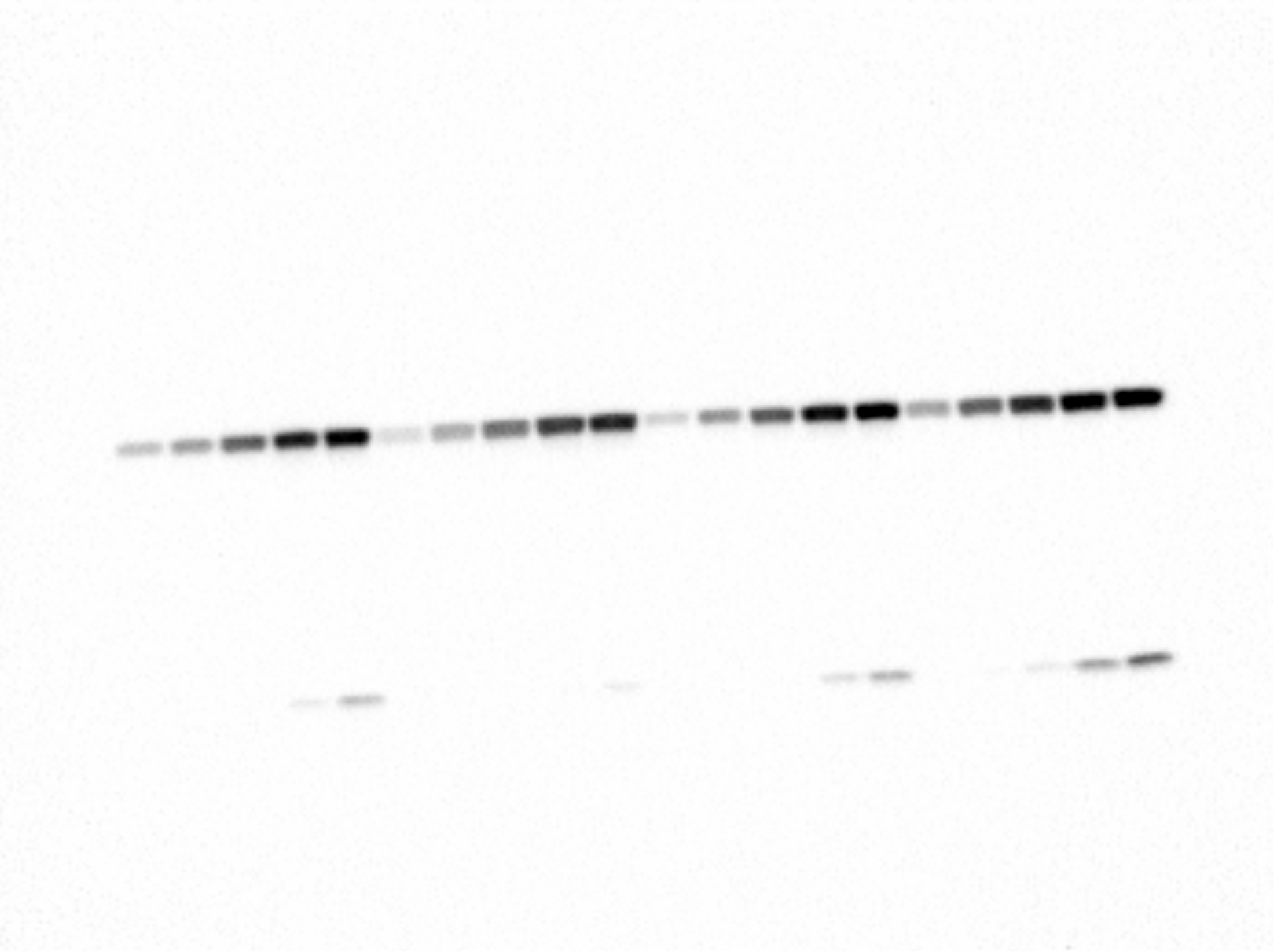

Supplement: Figure 2—source data 3. [file elife-80497-fig2-data3.zip › Figure 2-source data 3/Figure 2A and 2C/OM45_Expt2.tif]

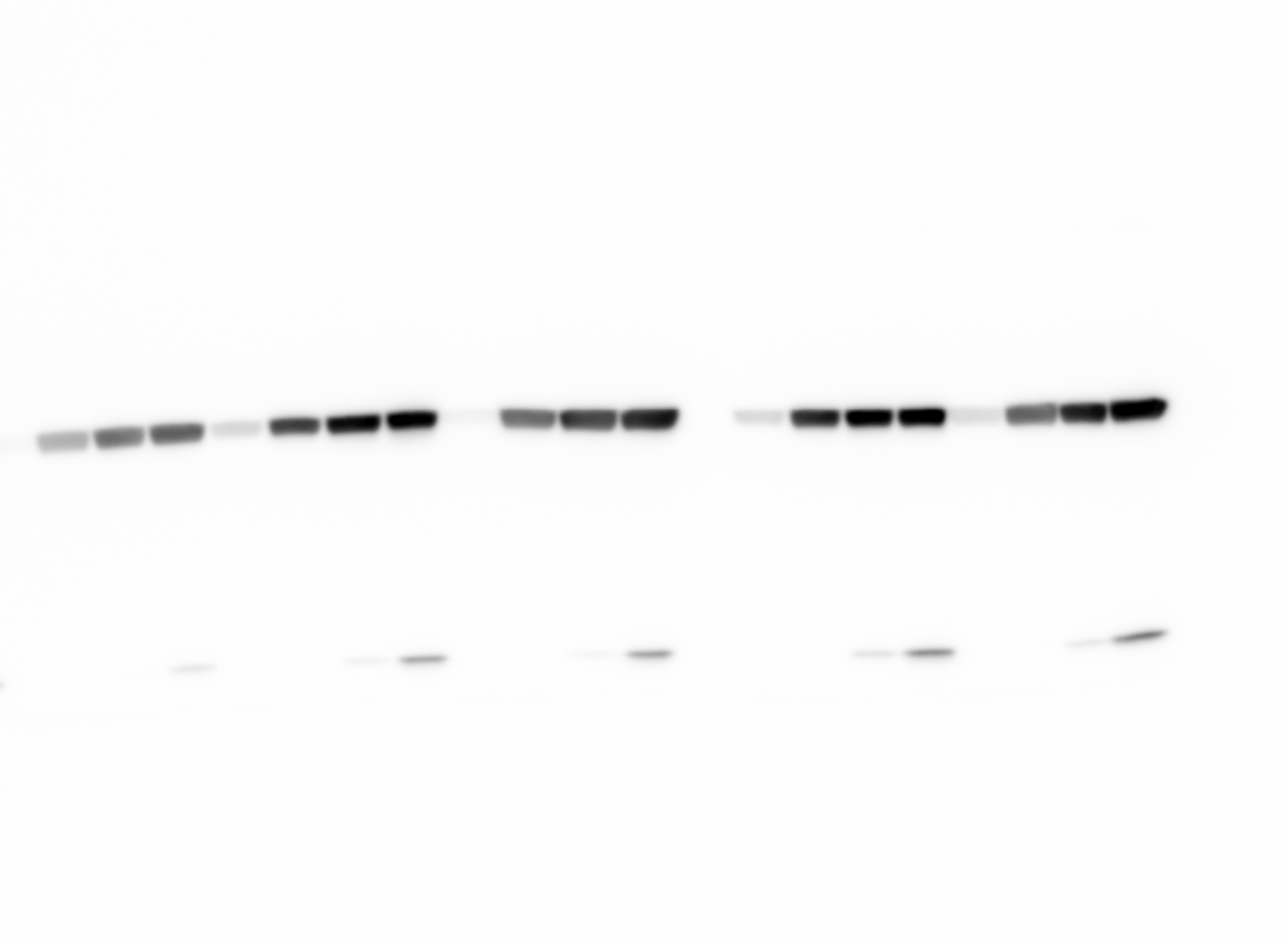

Supplement: Figure 2—source data 3. [file elife-80497-fig2-data3.zip › Figure 2-source data 3/Figure 2A and 2C/OM45_expt3_right.tif]

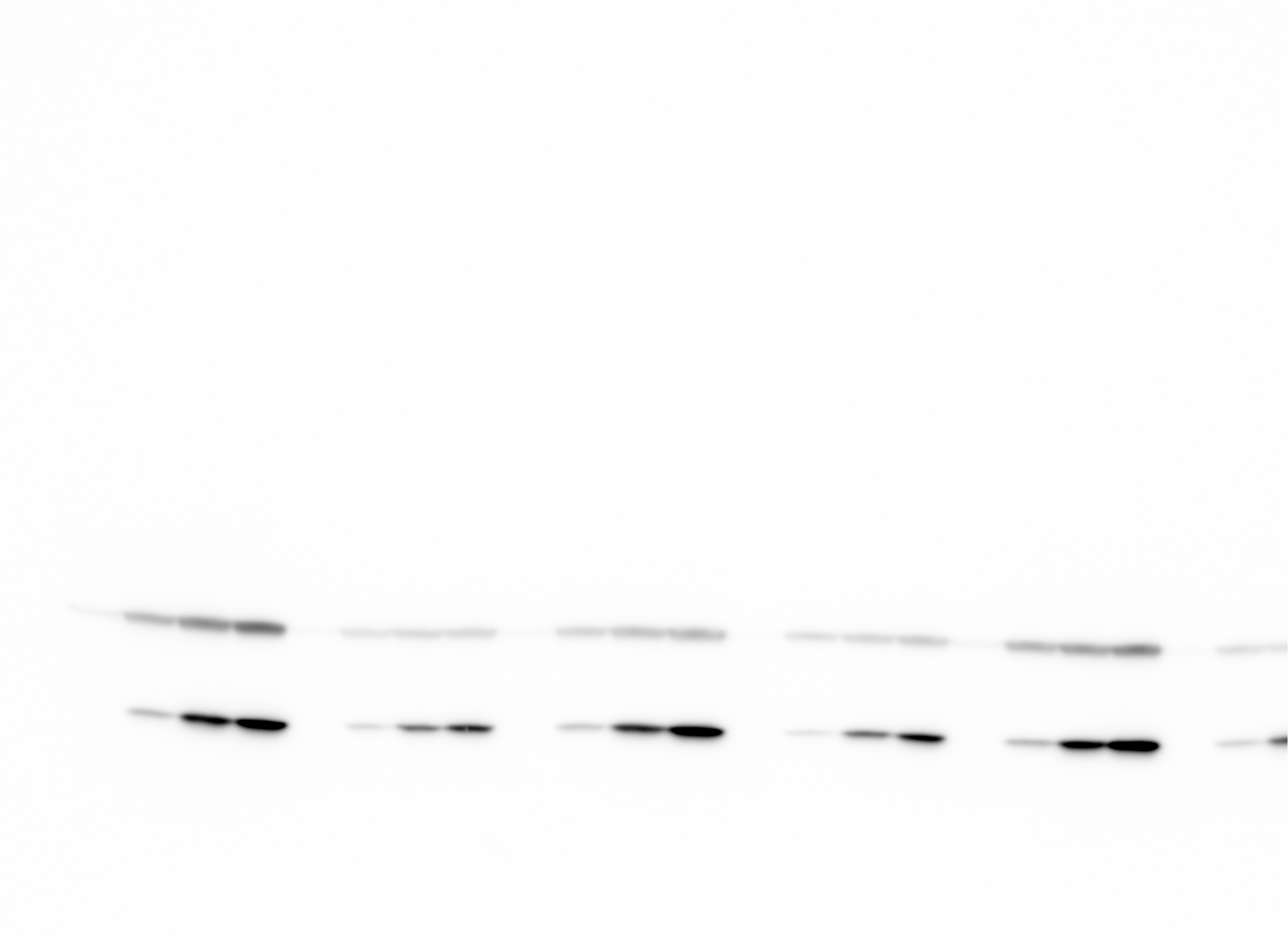

Supplement: Figure 2—source data 3. [file elife-80497-fig2-data3.zip › Figure 2-source data 3/Figure 2A and 2C/Atg8_expt3_left.tif]

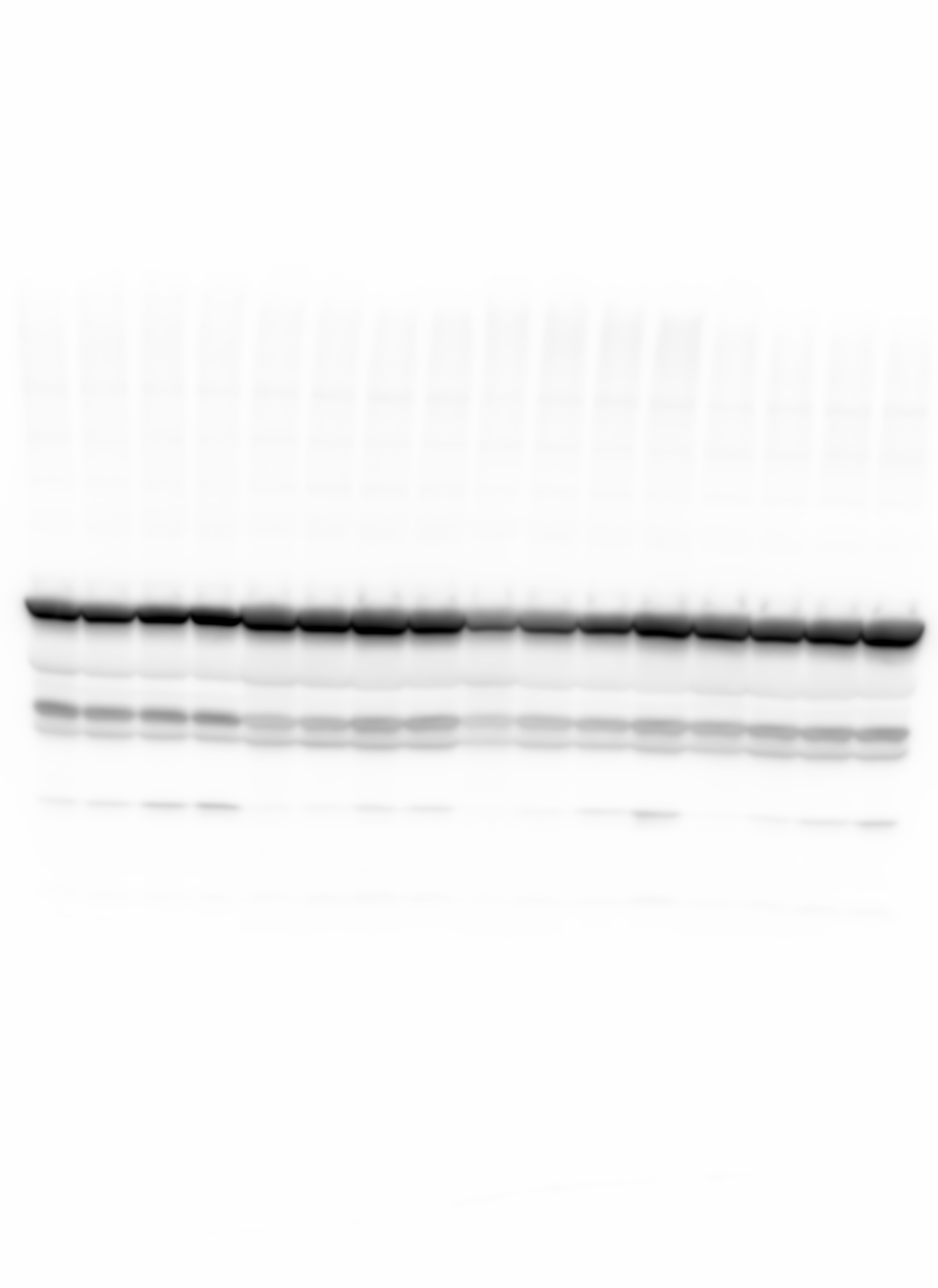

Supplement: Figure 2—source data 3. [file elife-80497-fig2-data3.zip › Figure 2-source data 3/Figure 2A and 2C/Atg8_expt4_G6PDH.tif]

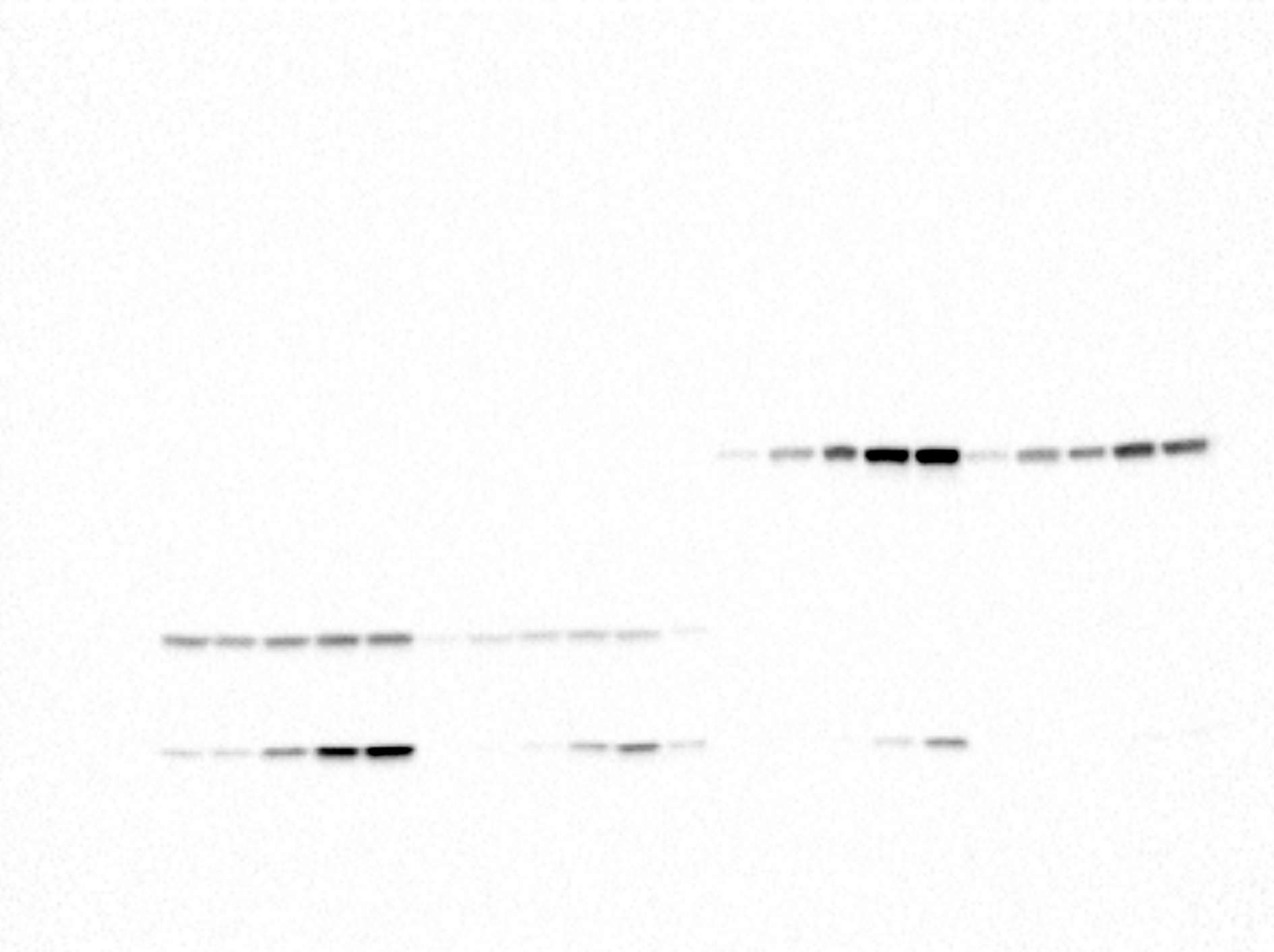

Supplement: Figure 2—source data 3. [file elife-80497-fig2-data3.zip › Figure 2-source data 3/Figure 2A and 2C/Atg8_OM45_Expts1.tif]

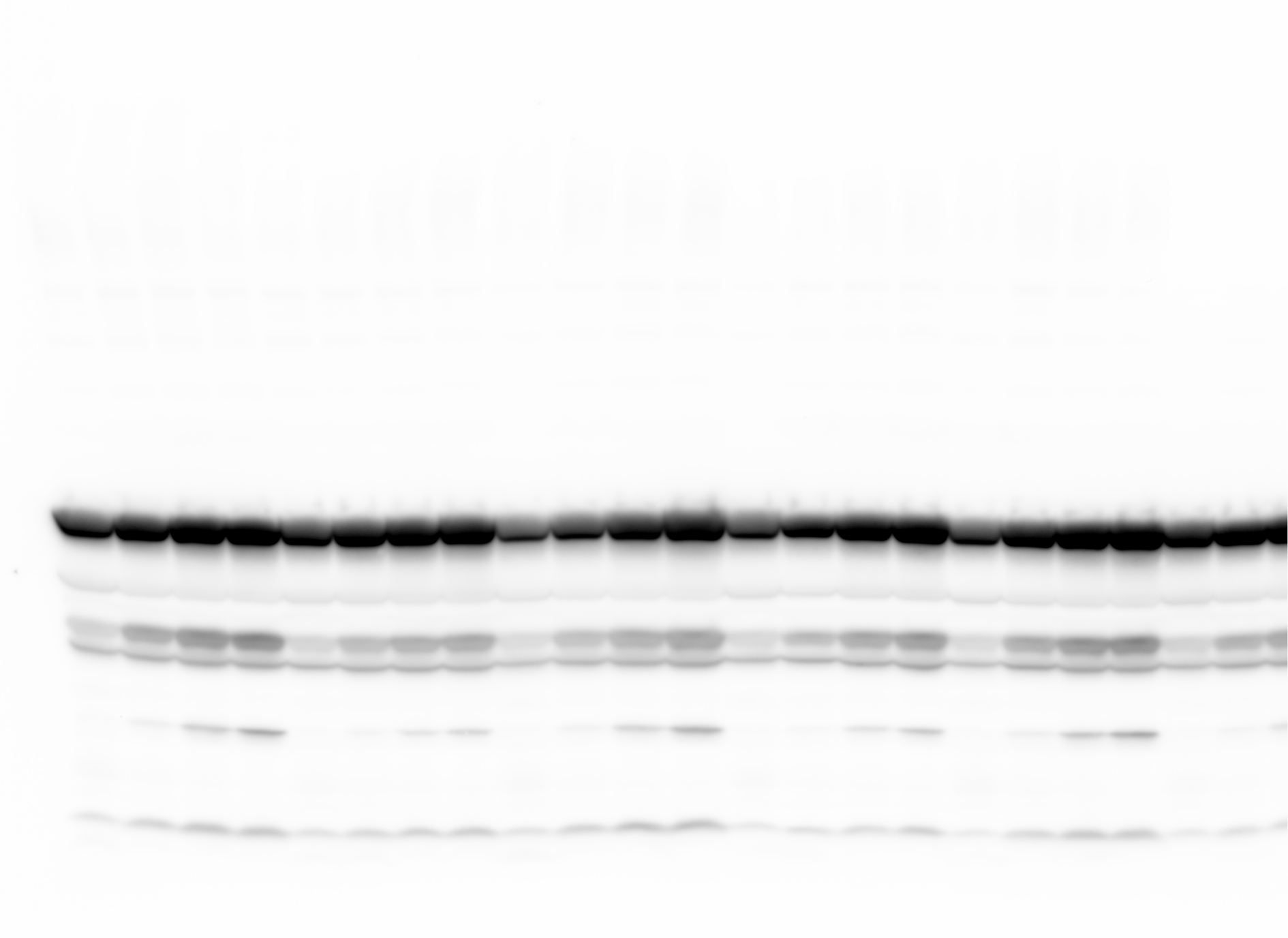

Supplement: Figure 2—source data 3. [file elife-80497-fig2-data3.zip › Figure 2-source data 3/Figure 2A and 2C/Atg8_expt3_G6PDH_left.tif]

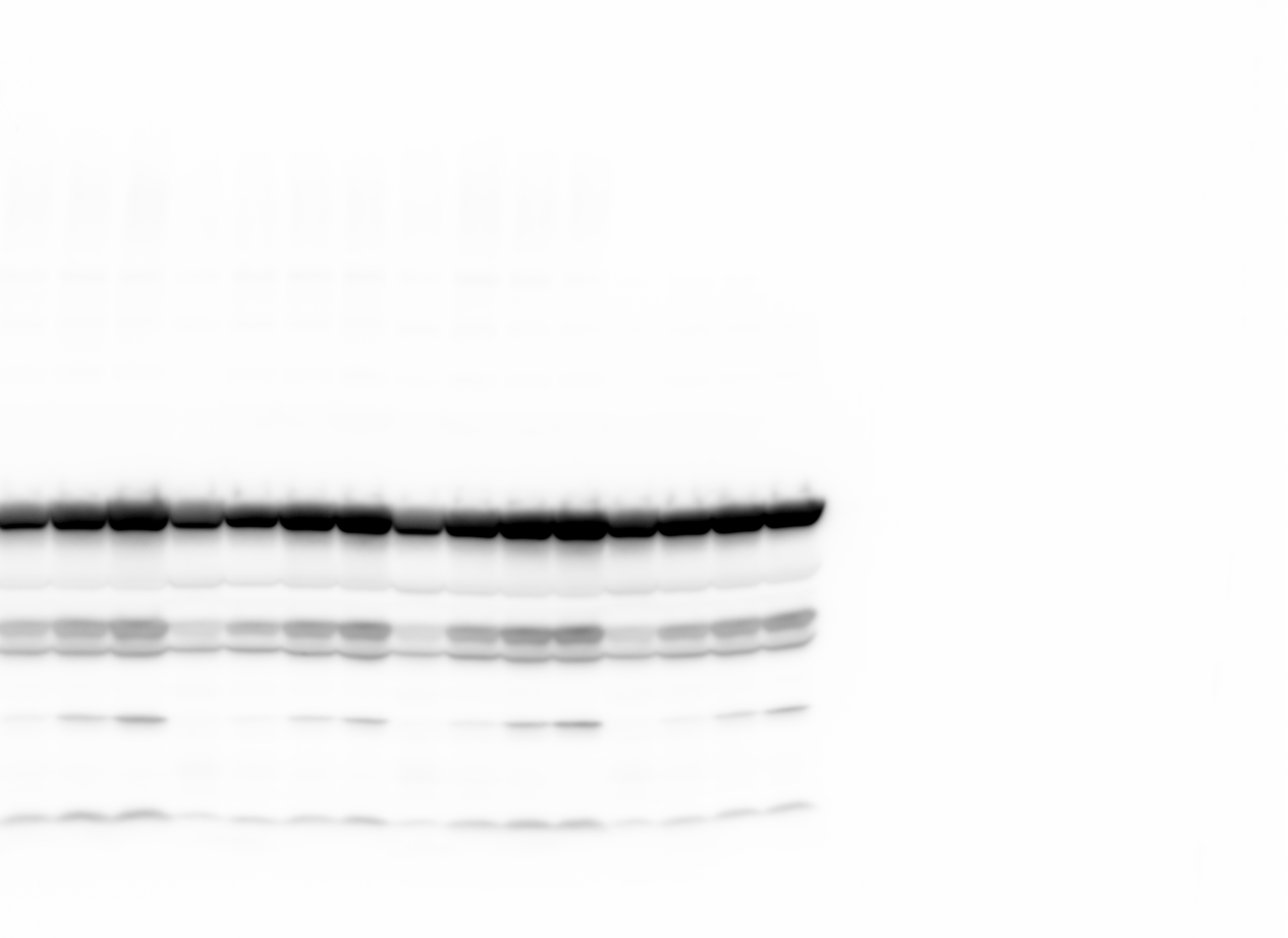

Supplement: Figure 2—source data 3. [file elife-80497-fig2-data3.zip › Figure 2-source data 3/Figure 2A and 2C/Atg8_expt3_G6PDH_right.tif]

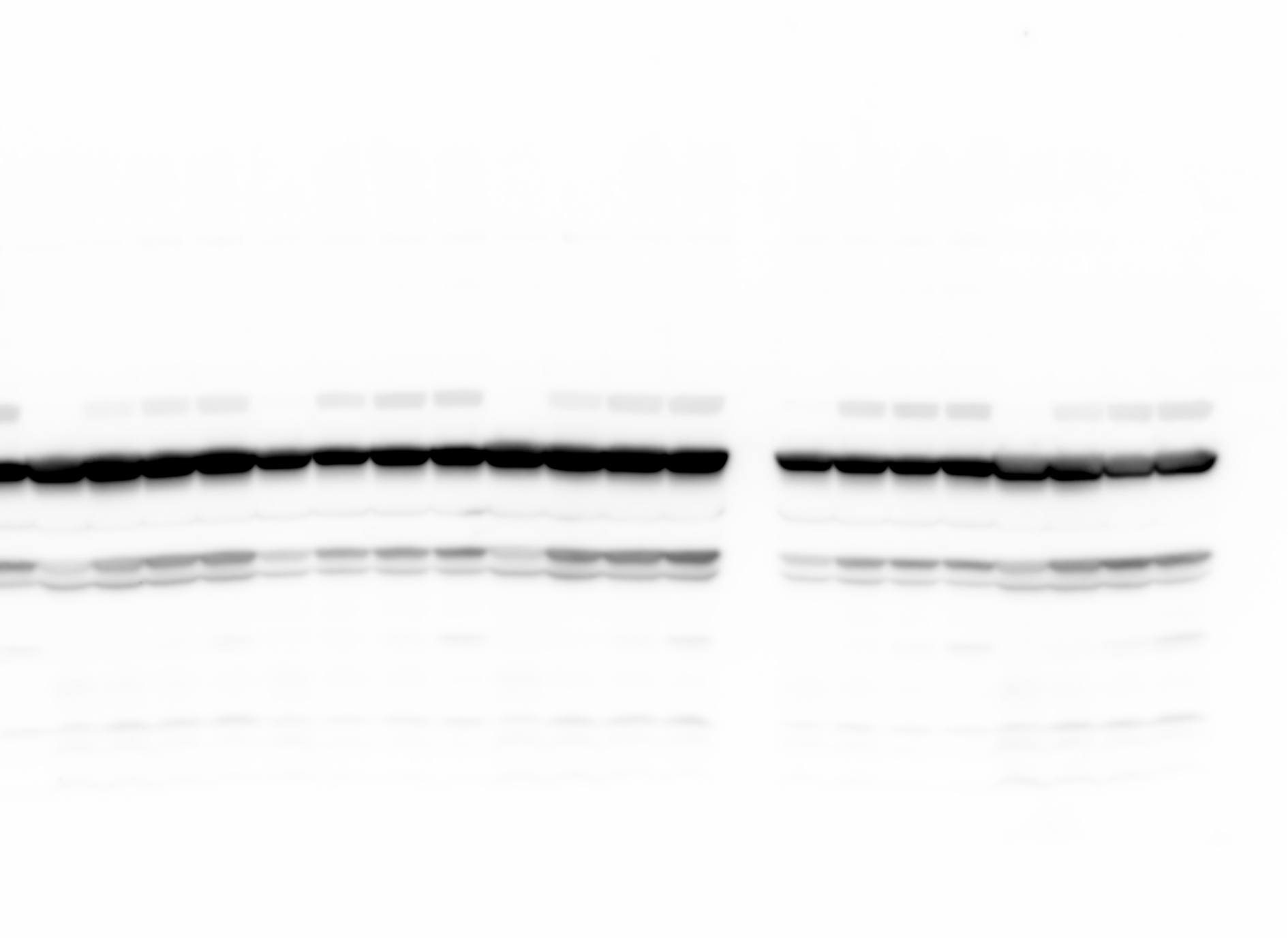

Supplement: Figure 2—source data 3. [file elife-80497-fig2-data3.zip › Figure 2-source data 3/Figure 2A and 2C/OM45_expt3_G6PDH_right.tif]

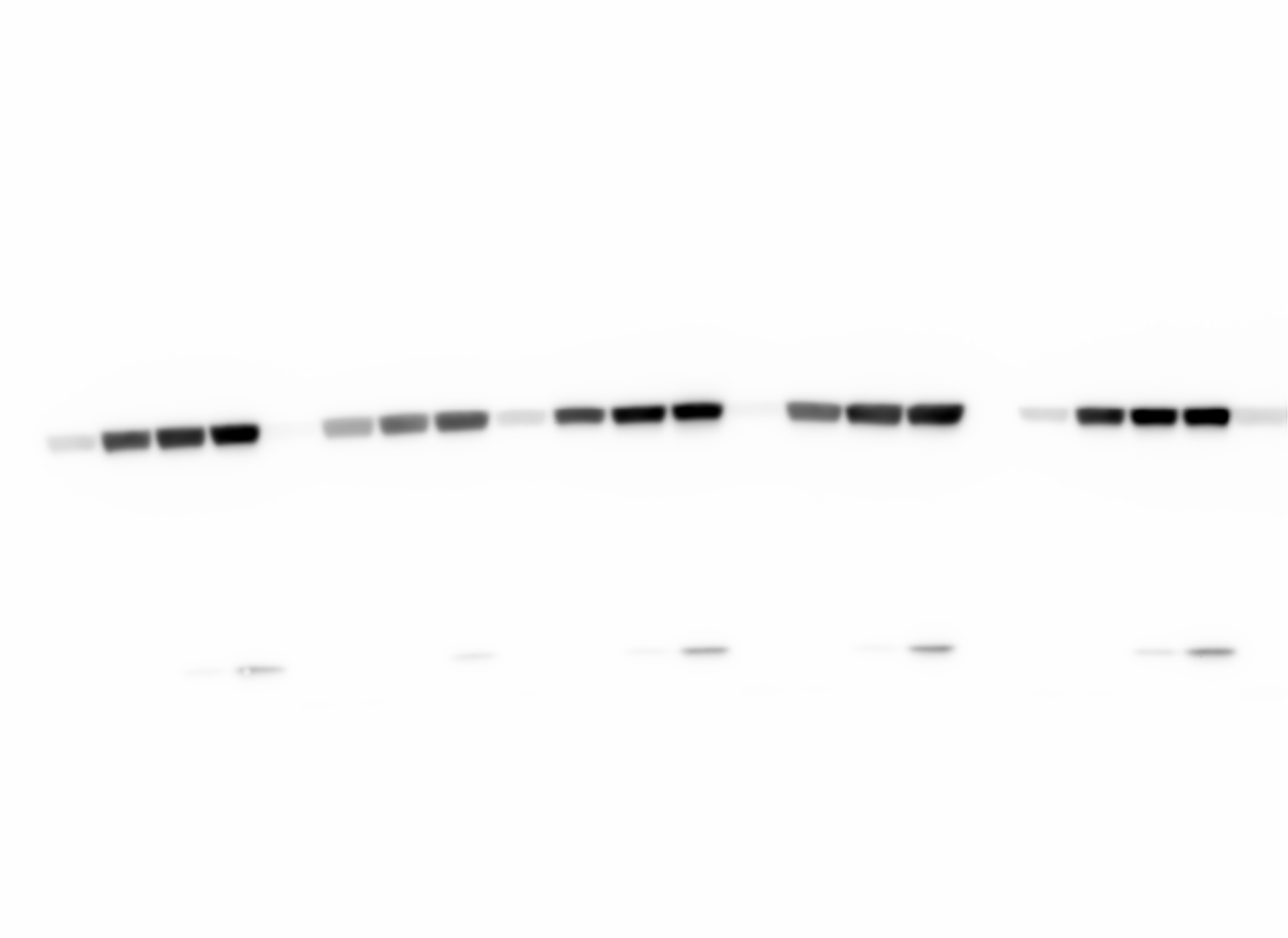

Supplement: Figure 2—source data 3. [file elife-80497-fig2-data3.zip › Figure 2-source data 3/Figure 2A and 2C/OM45_Exp3_left.tif]

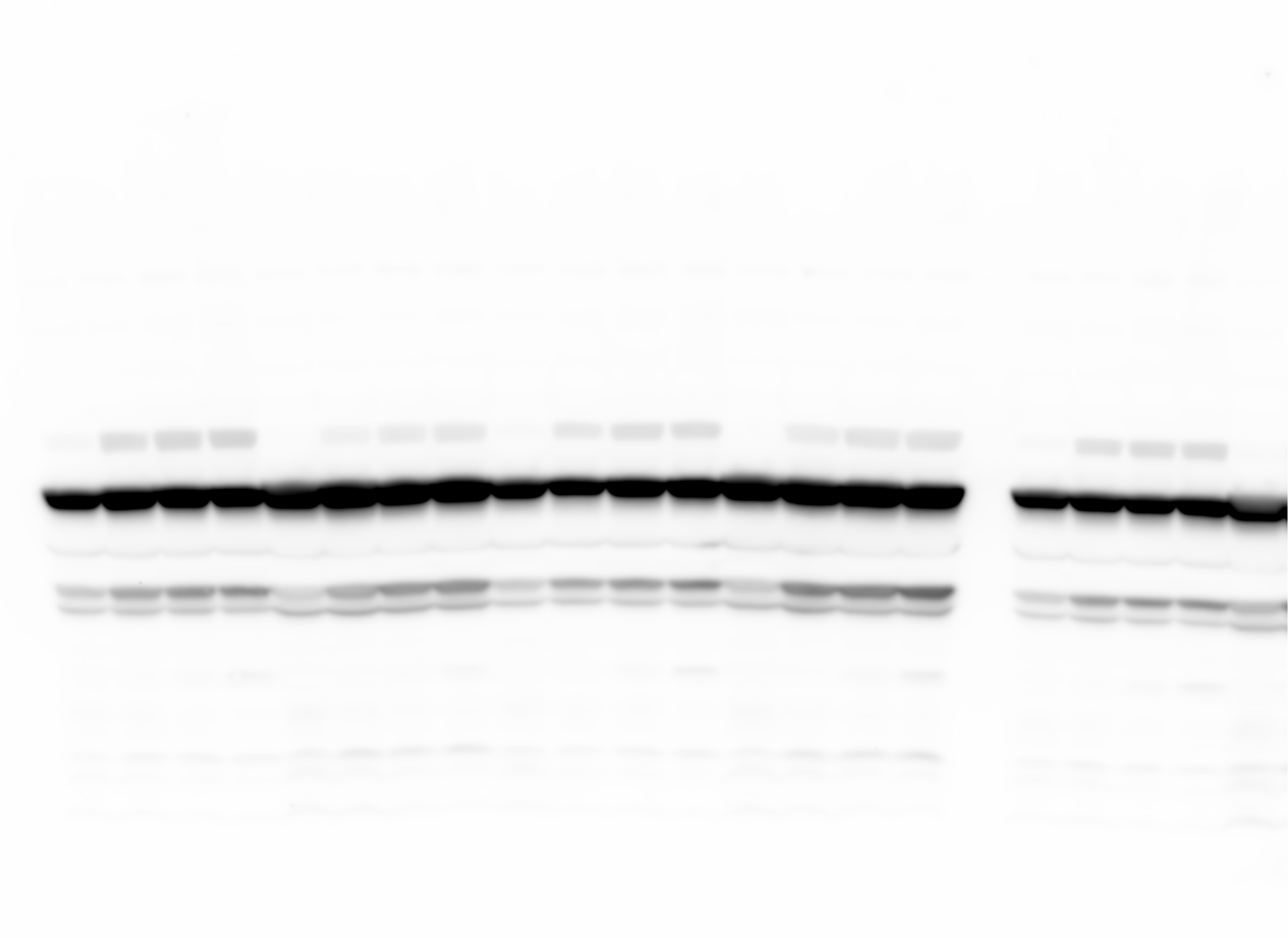

Supplement: Figure 2—source data 3. [file elife-80497-fig2-data3.zip › Figure 2-source data 3/Figure 2A and 2C/OM45_Expt3_G6PDH_left.tif]

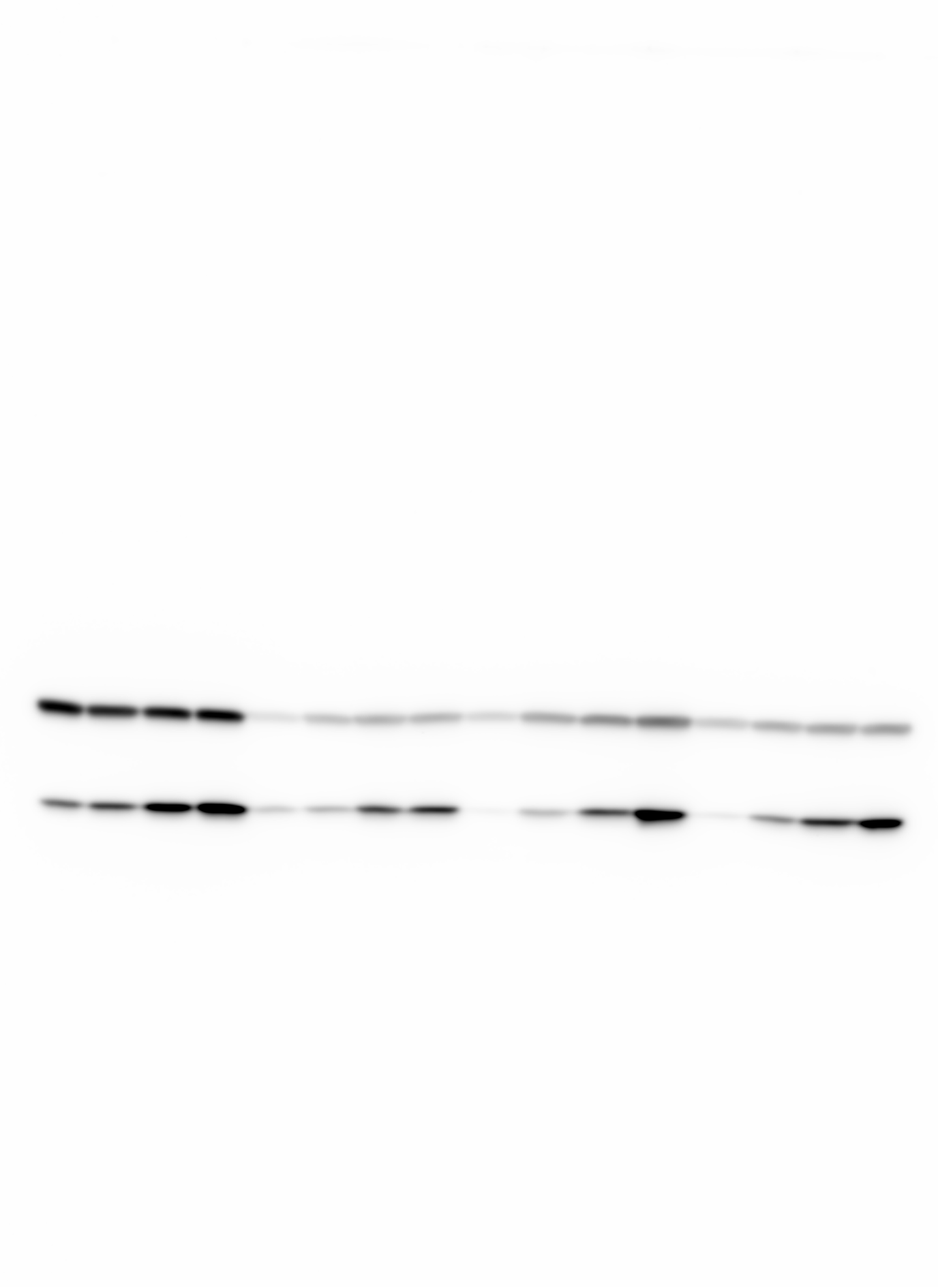

Supplement: Figure 2—source data 3. [file elife-80497-fig2-data3.zip › Figure 2-source data 3/Figure 2A and 2C/Atg8_expt4.tif]

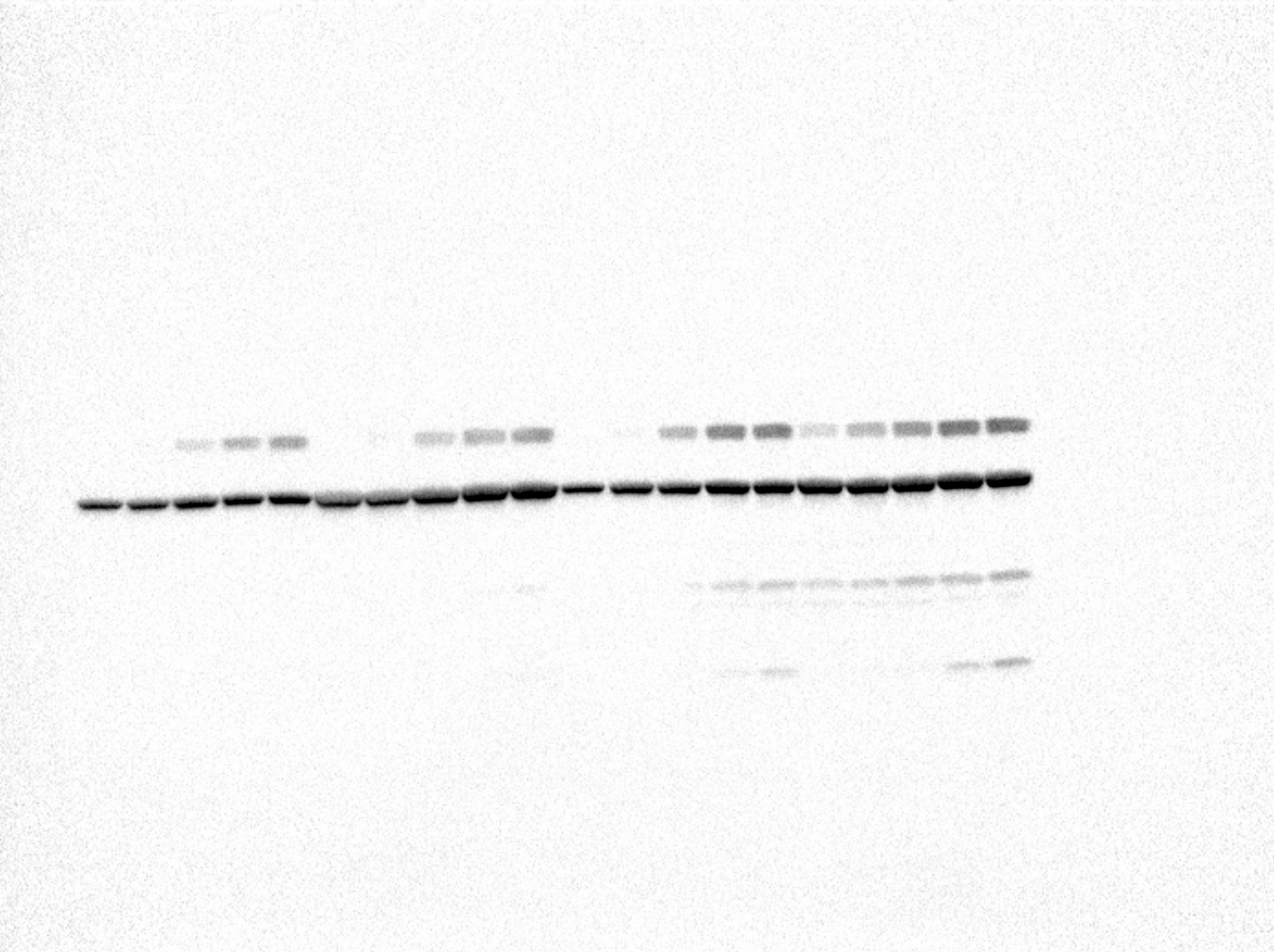

Supplement: Figure 2—source data 3. [file elife-80497-fig2-data3.zip › Figure 2-source data 3/Figure 2A and 2C/OM45_Expt2_G6PDH.tif]

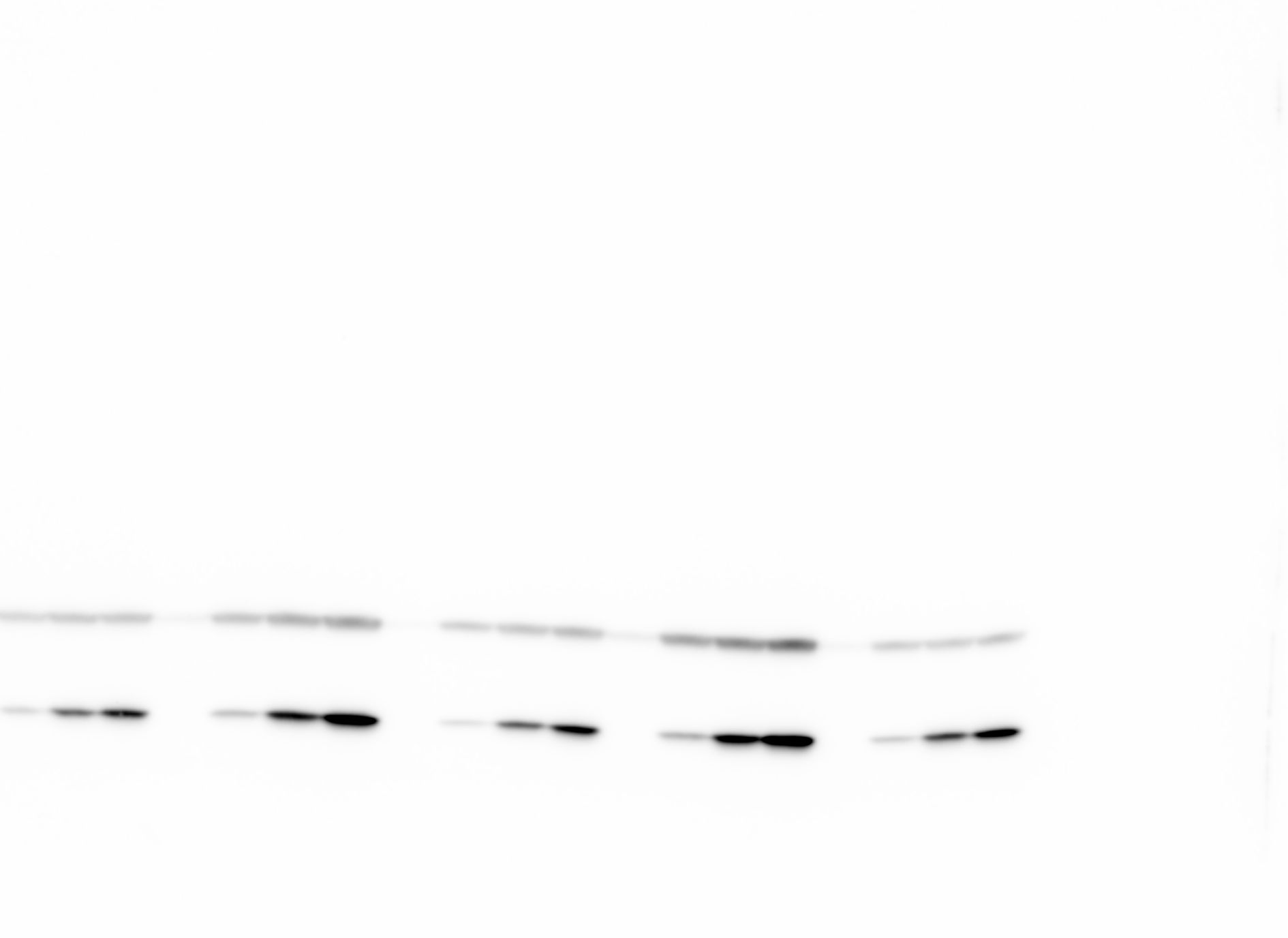

Supplement: Figure 2—source data 3. [file elife-80497-fig2-data3.zip › Figure 2-source data 3/Figure 2A and 2C/Atg8_expt3_right.tif]

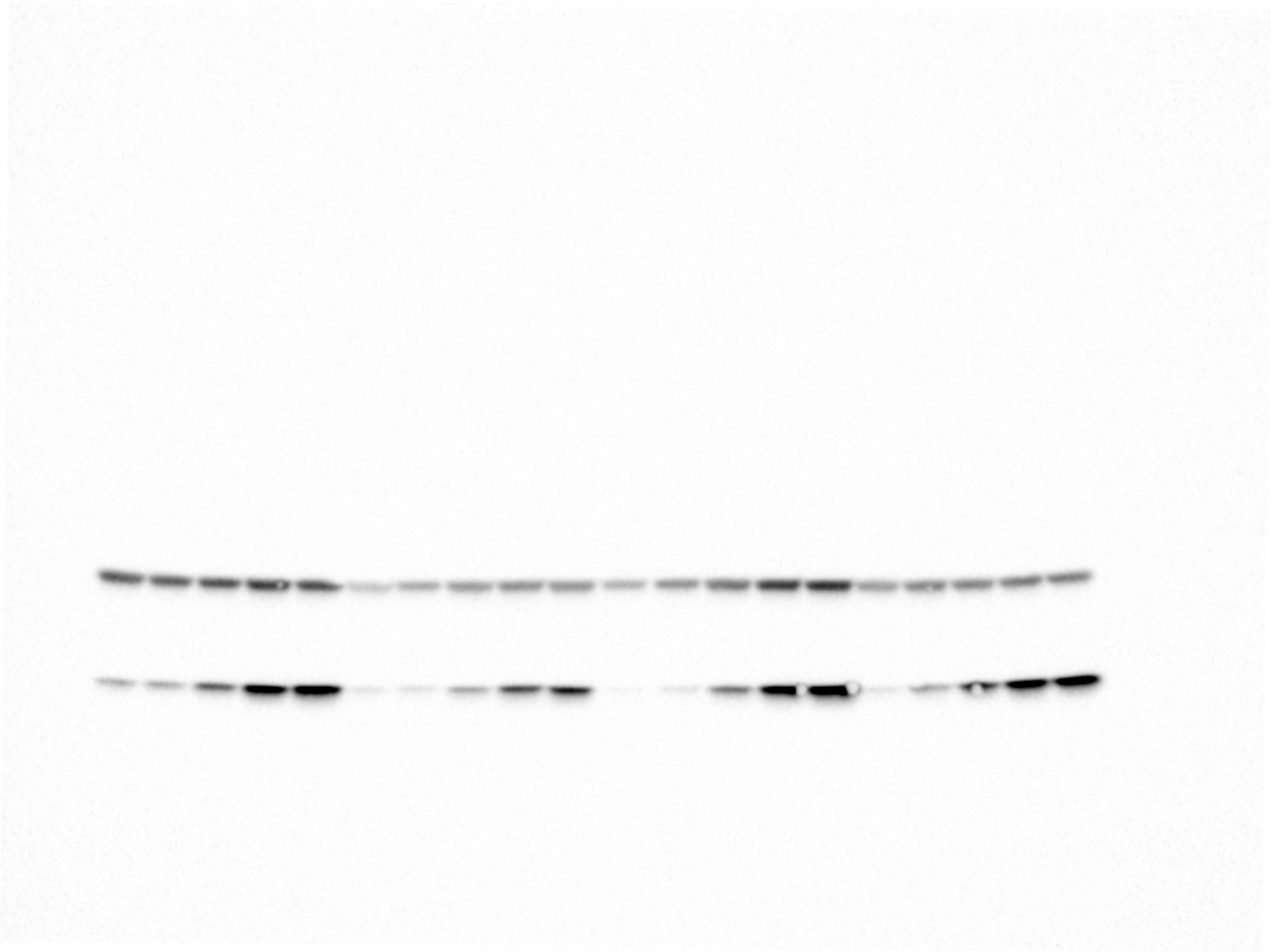

Supplement: Figure 2—source data 3. [file elife-80497-fig2-data3.zip › Figure 2-source data 3/Figure 2A and 2C/Atg8_Expt2.tif]

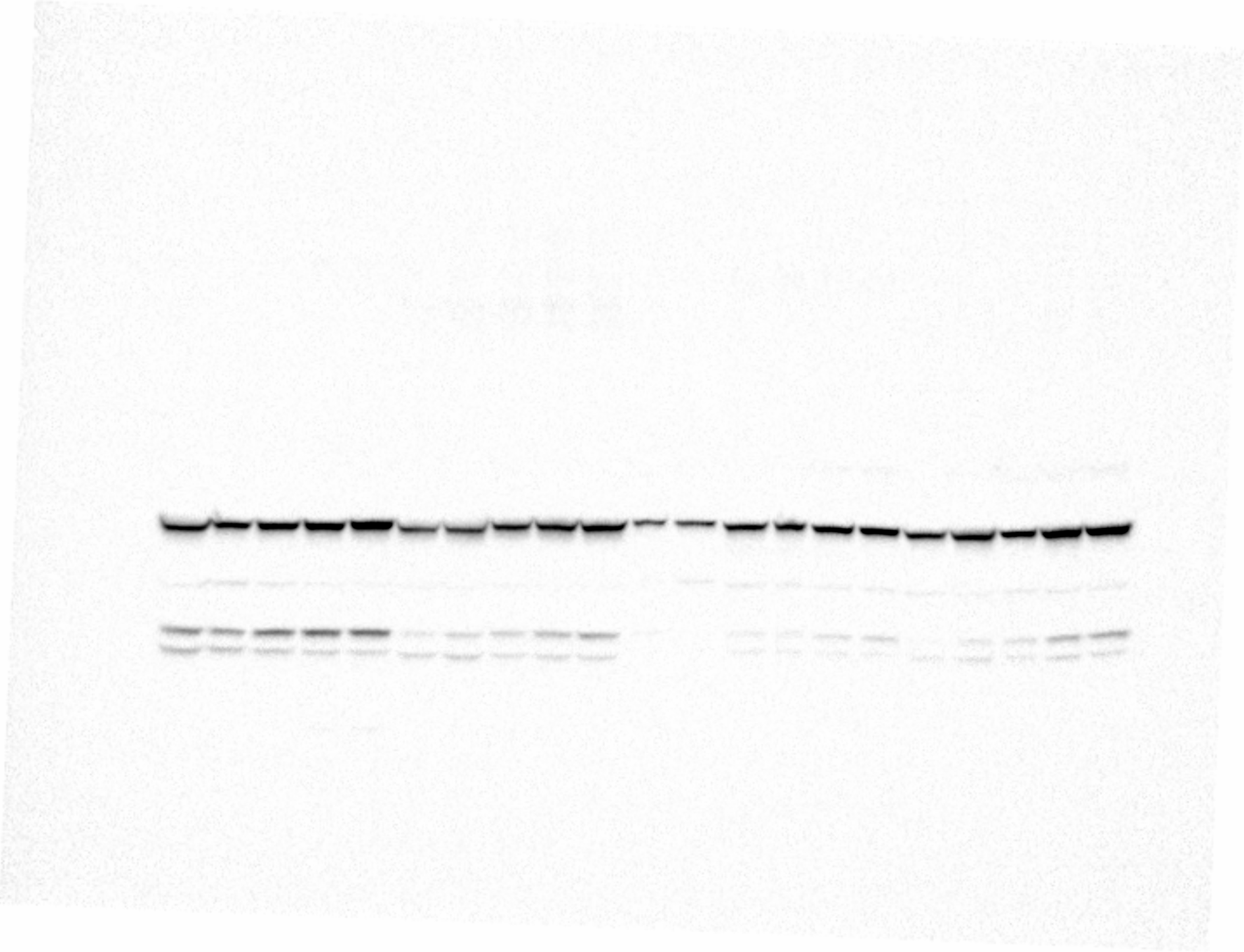

Supplement: Figure 2—source data 3. [file elife-80497-fig2-data3.zip › Figure 2-source data 3/Figure 2A and 2C/Atg8_OM45_Expts1_G6PDH.tif]

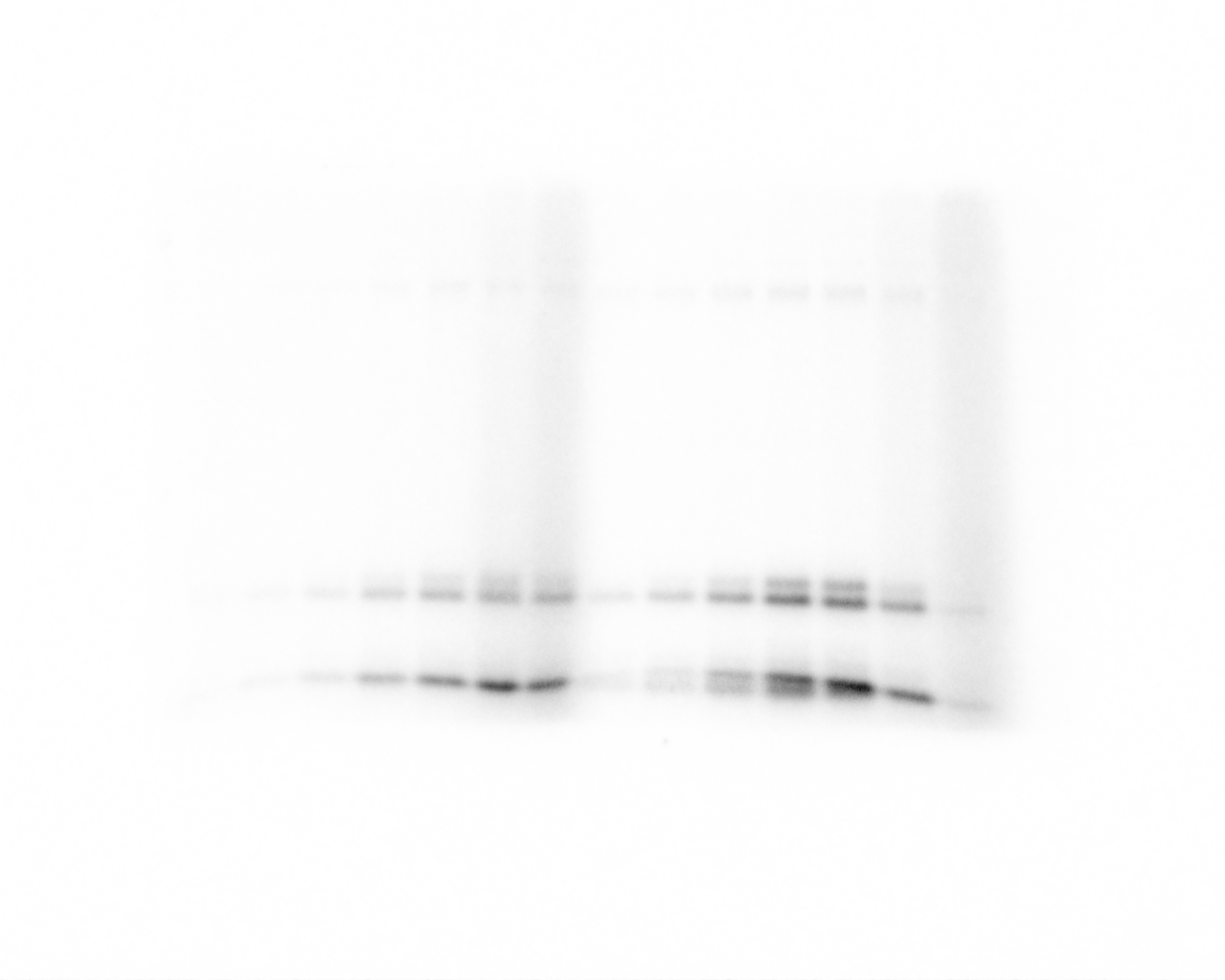

Supplement: Figure 3—source data 3. [file elife-80497-fig3-data3.zip › Figure 3-source data 3/Figure 3C/32P/Replica 4.tif]

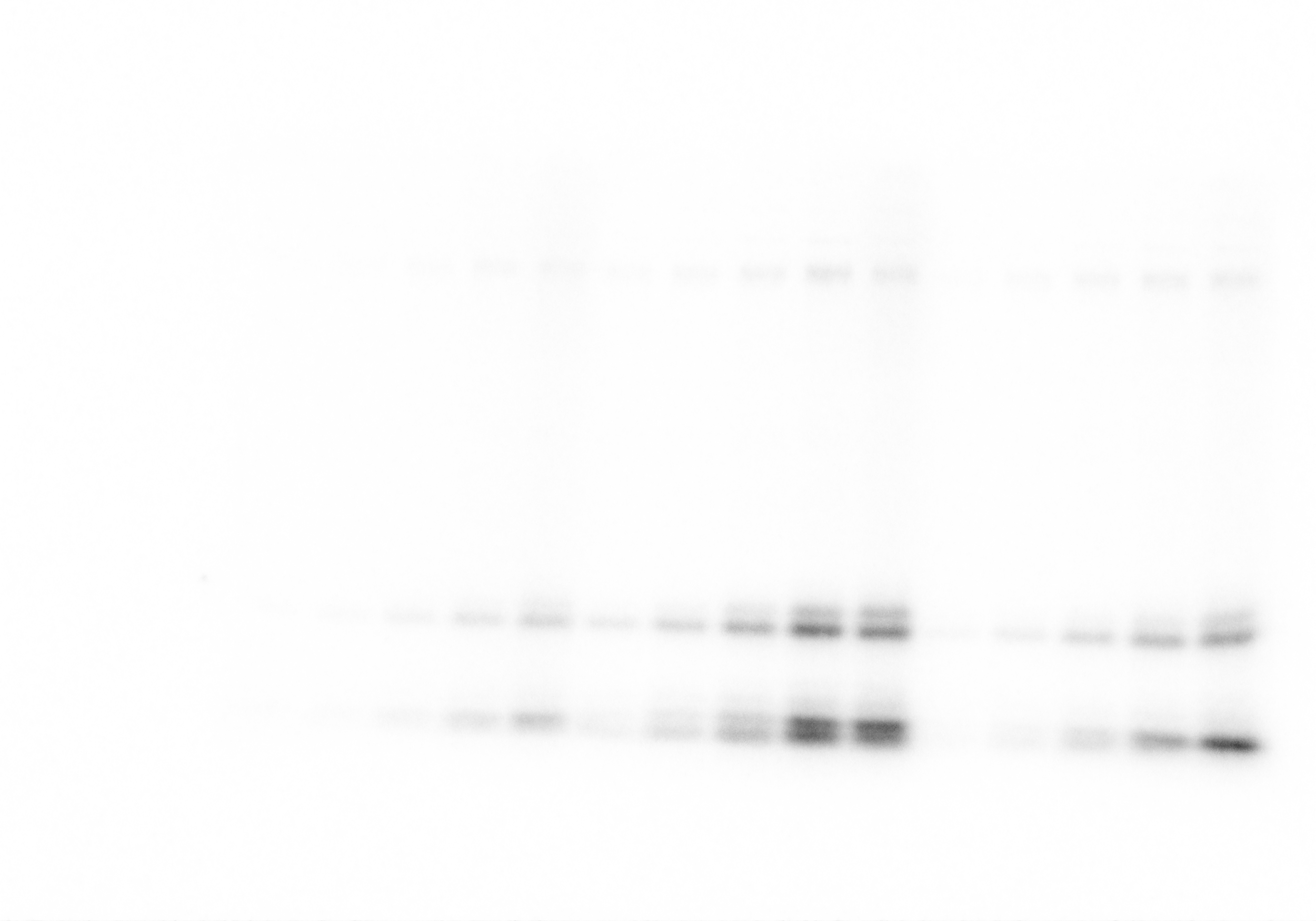

Supplement: Figure 3—source data 3. [file elife-80497-fig3-data3.zip › Figure 3-source data 3/Figure 3C/32P/Replica 2.tif]

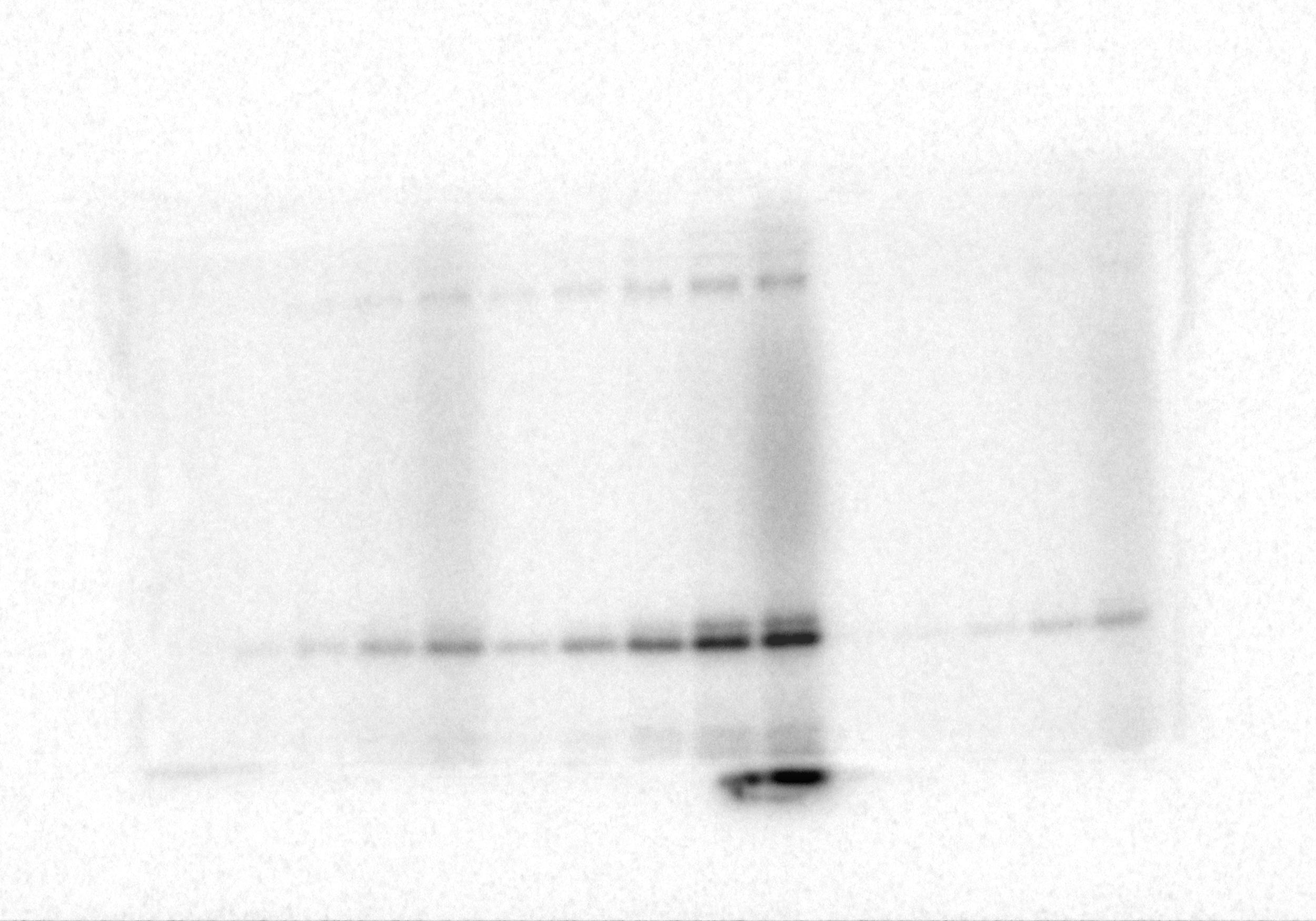

Supplement: Figure 3—source data 3. [file elife-80497-fig3-data3.zip › Figure 3-source data 3/Figure 3C/32P/Replica 3.tif]

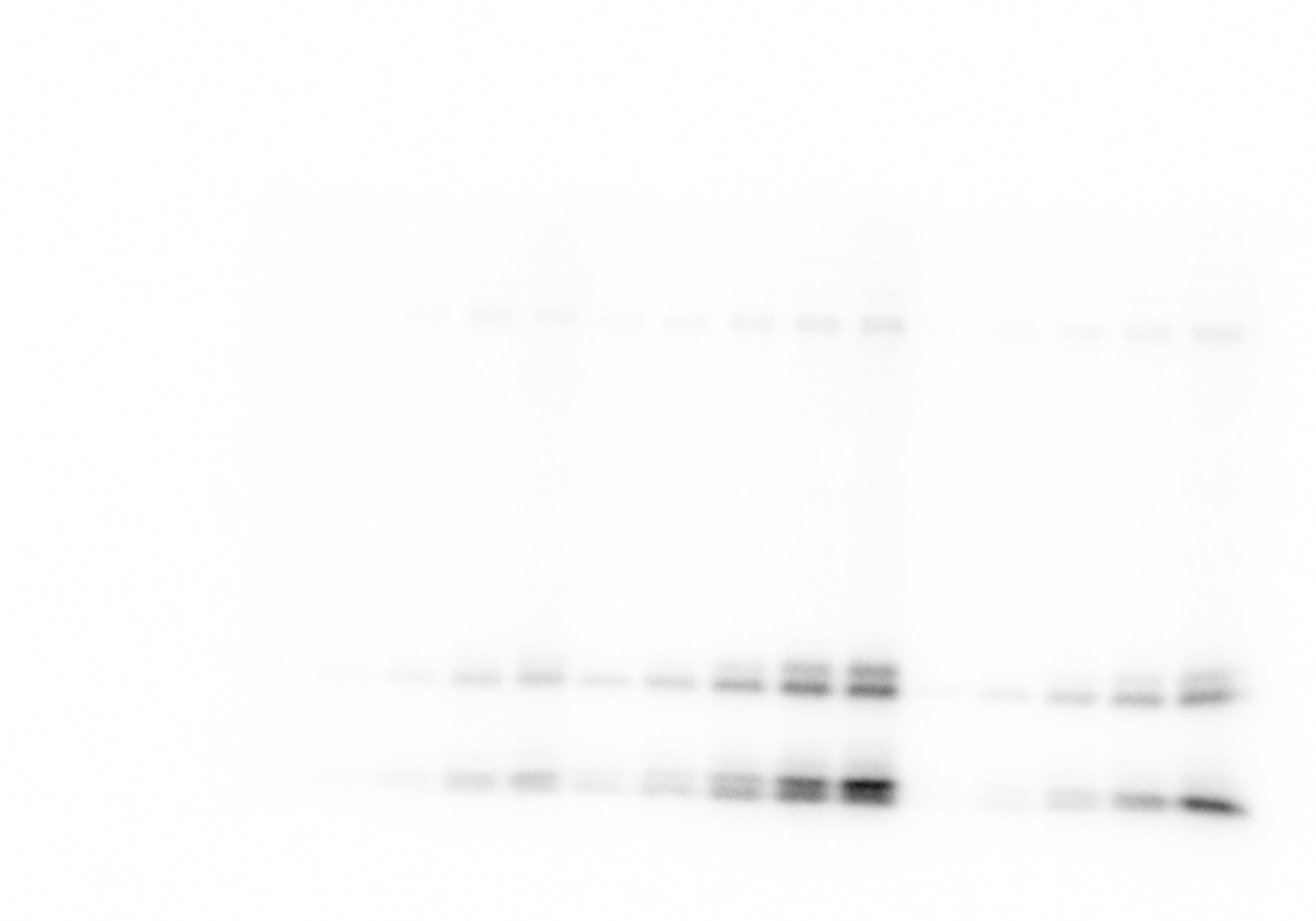

Supplement: Figure 3—source data 3. [file elife-80497-fig3-data3.zip › Figure 3-source data 3/Figure 3C/32P/Replica 1.tif]

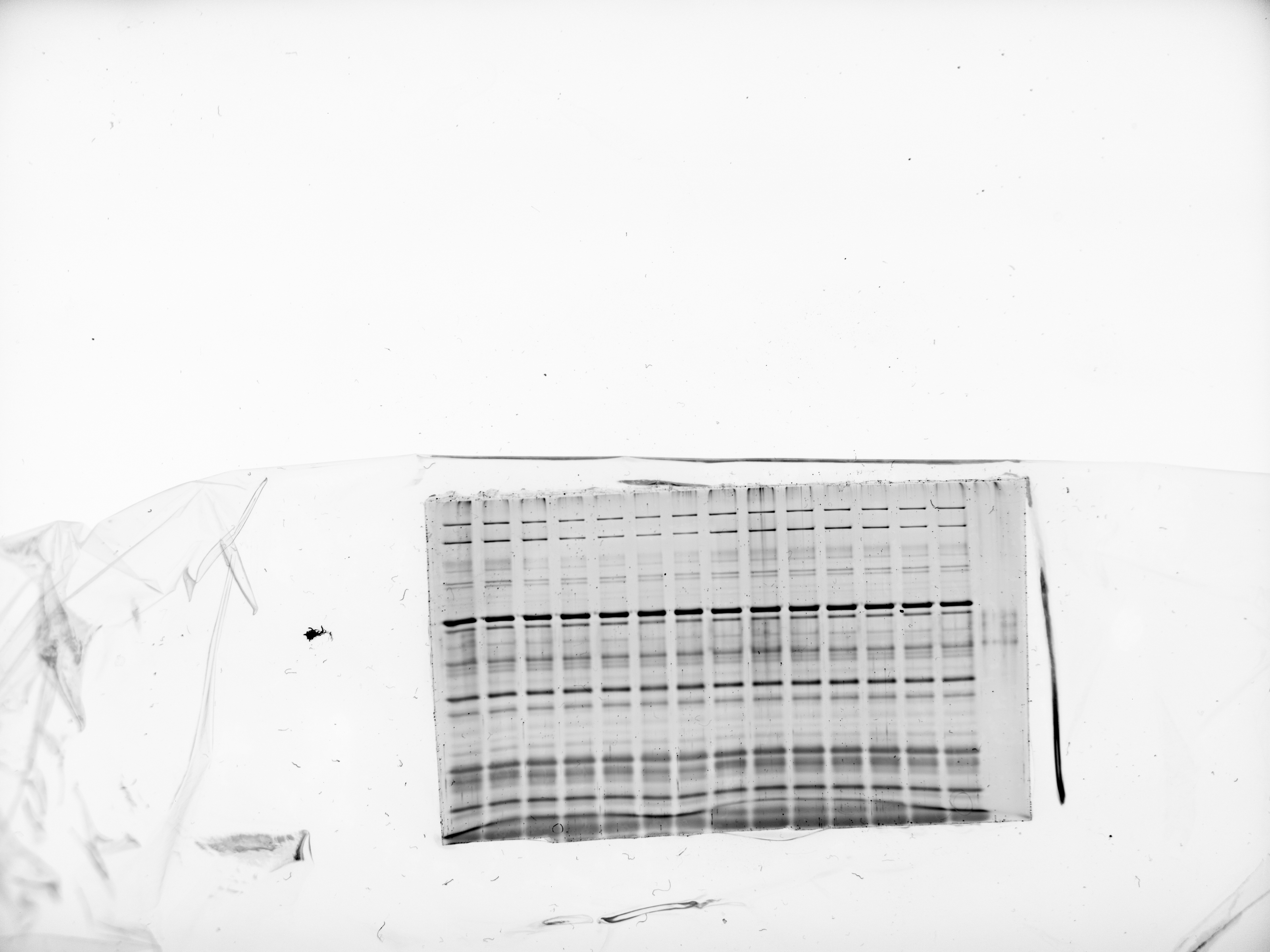

Supplement: Figure 3—source data 3. [file elife-80497-fig3-data3.zip › Figure 3-source data 3/Figure 3C/Sypro/Replica 4.tif]

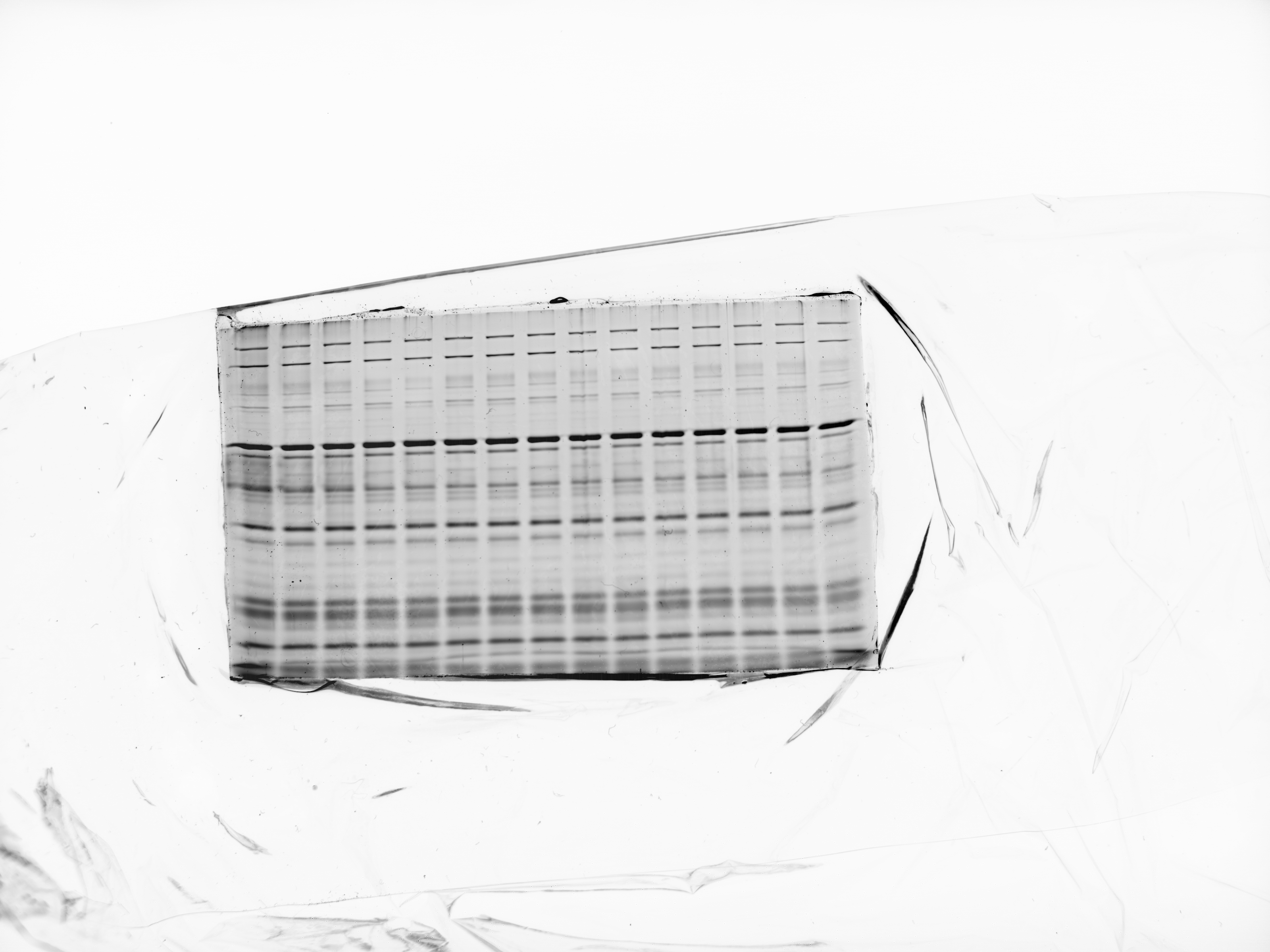

Supplement: Figure 3—source data 3. [file elife-80497-fig3-data3.zip › Figure 3-source data 3/Figure 3C/Sypro/Replica 2.tif]

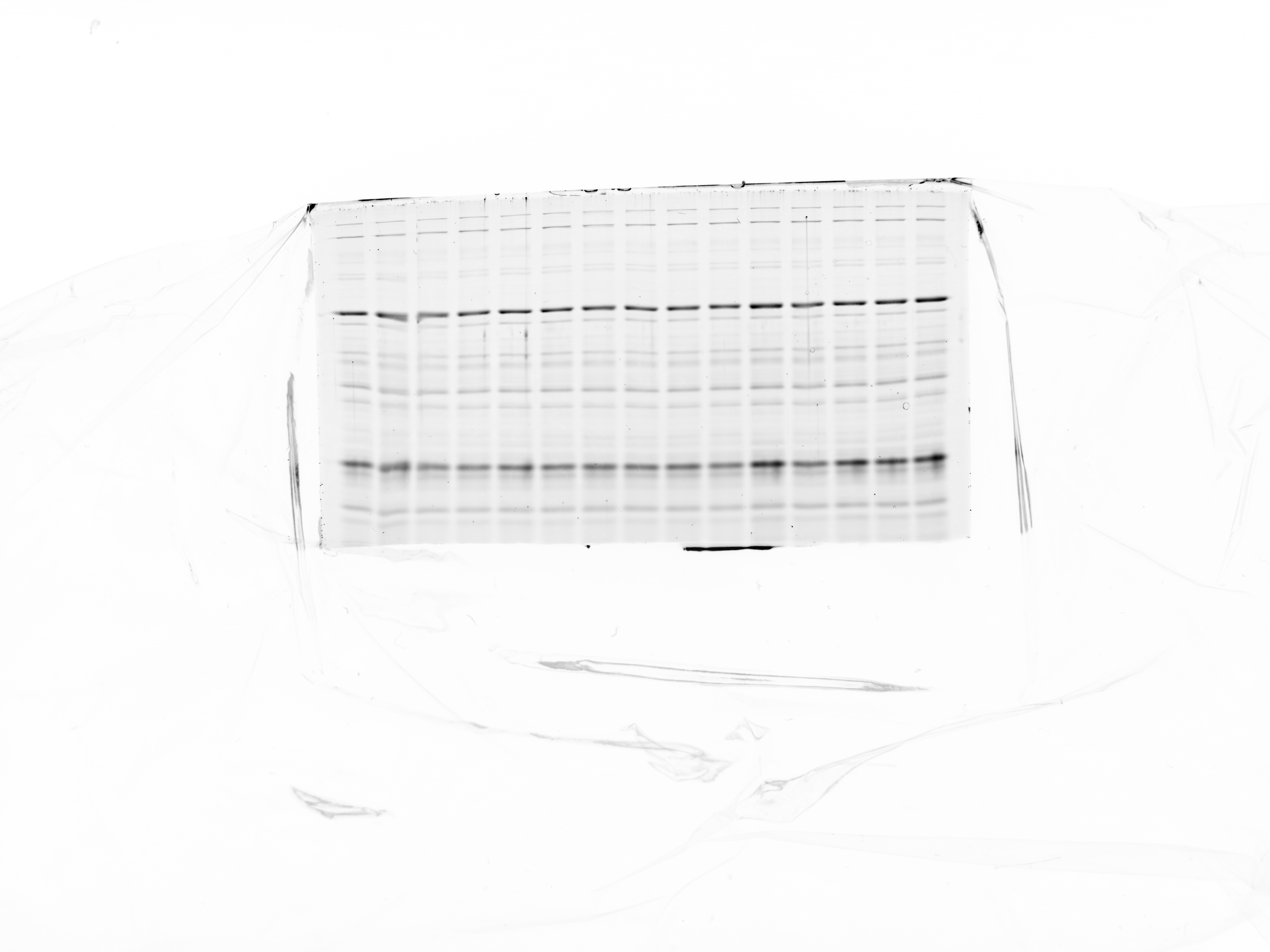

Supplement: Figure 3—source data 3. [file elife-80497-fig3-data3.zip › Figure 3-source data 3/Figure 3C/Sypro/Replica 3.tif]

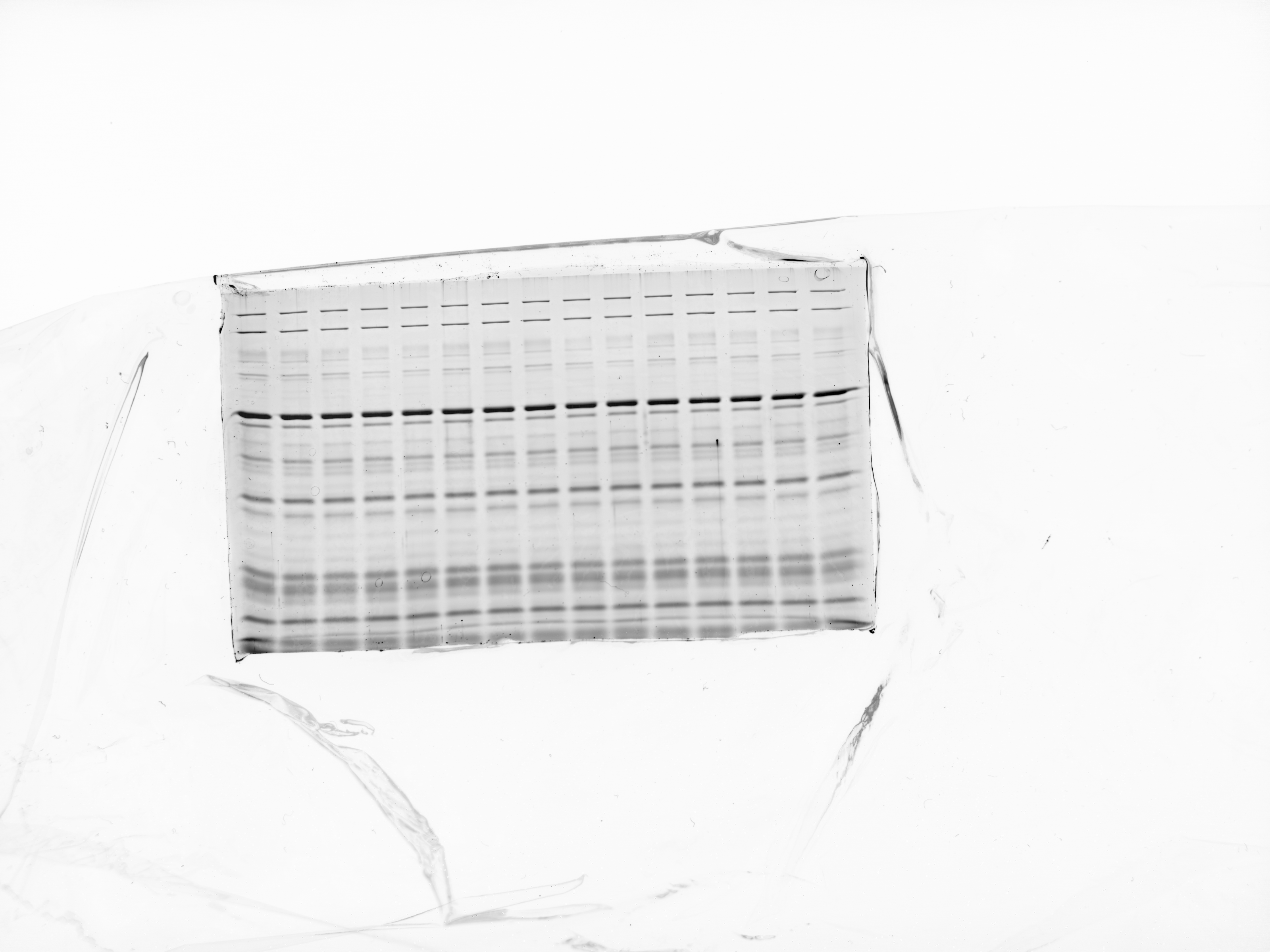

Supplement: Figure 3—source data 3. [file elife-80497-fig3-data3.zip › Figure 3-source data 3/Figure 3C/Sypro/Replica 1.tif]

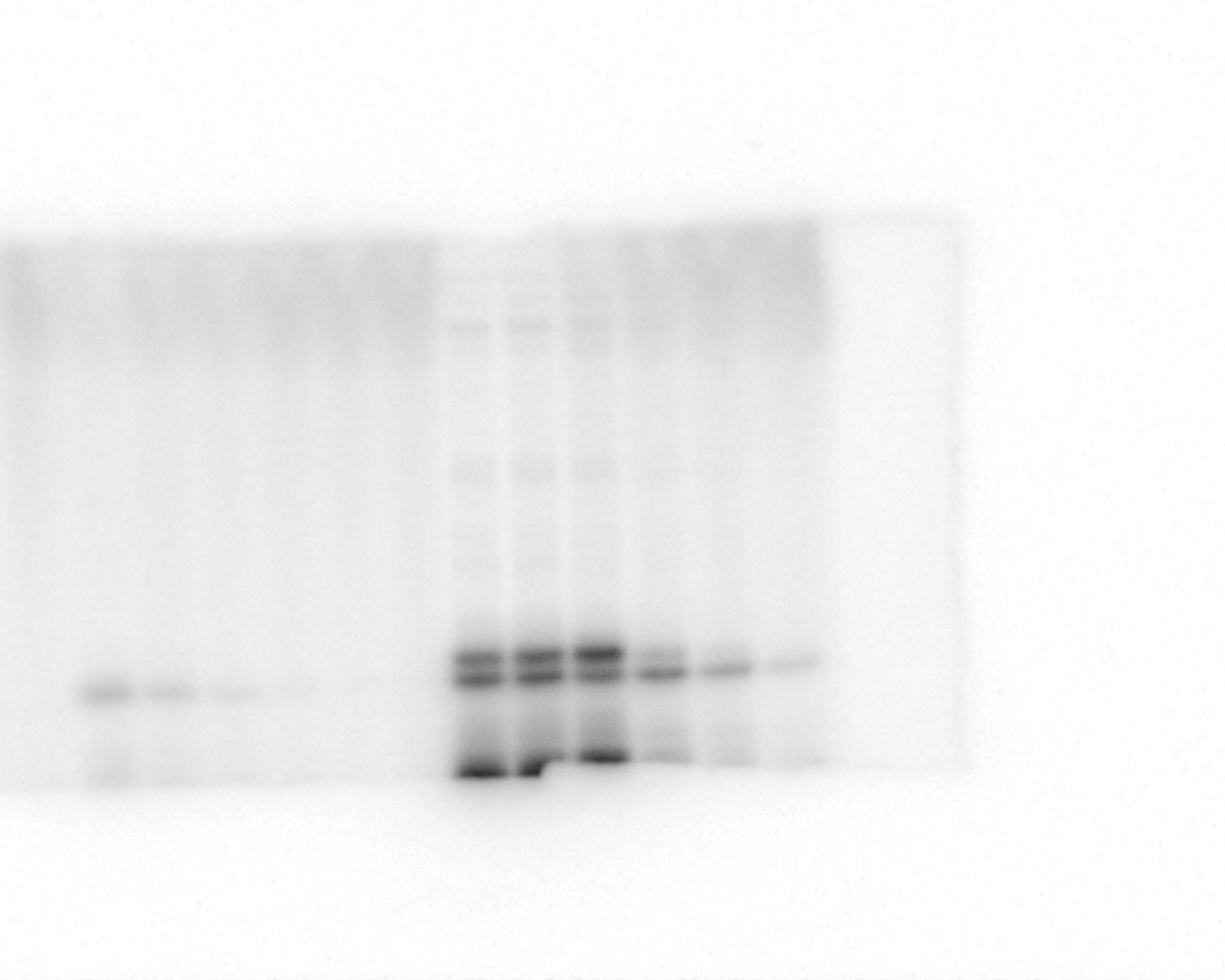

Supplement: Figure 3—source data 3. [file elife-80497-fig3-data3.zip › Figure 3-source data 3/Figure 3A/32P/Replica 2.tif]

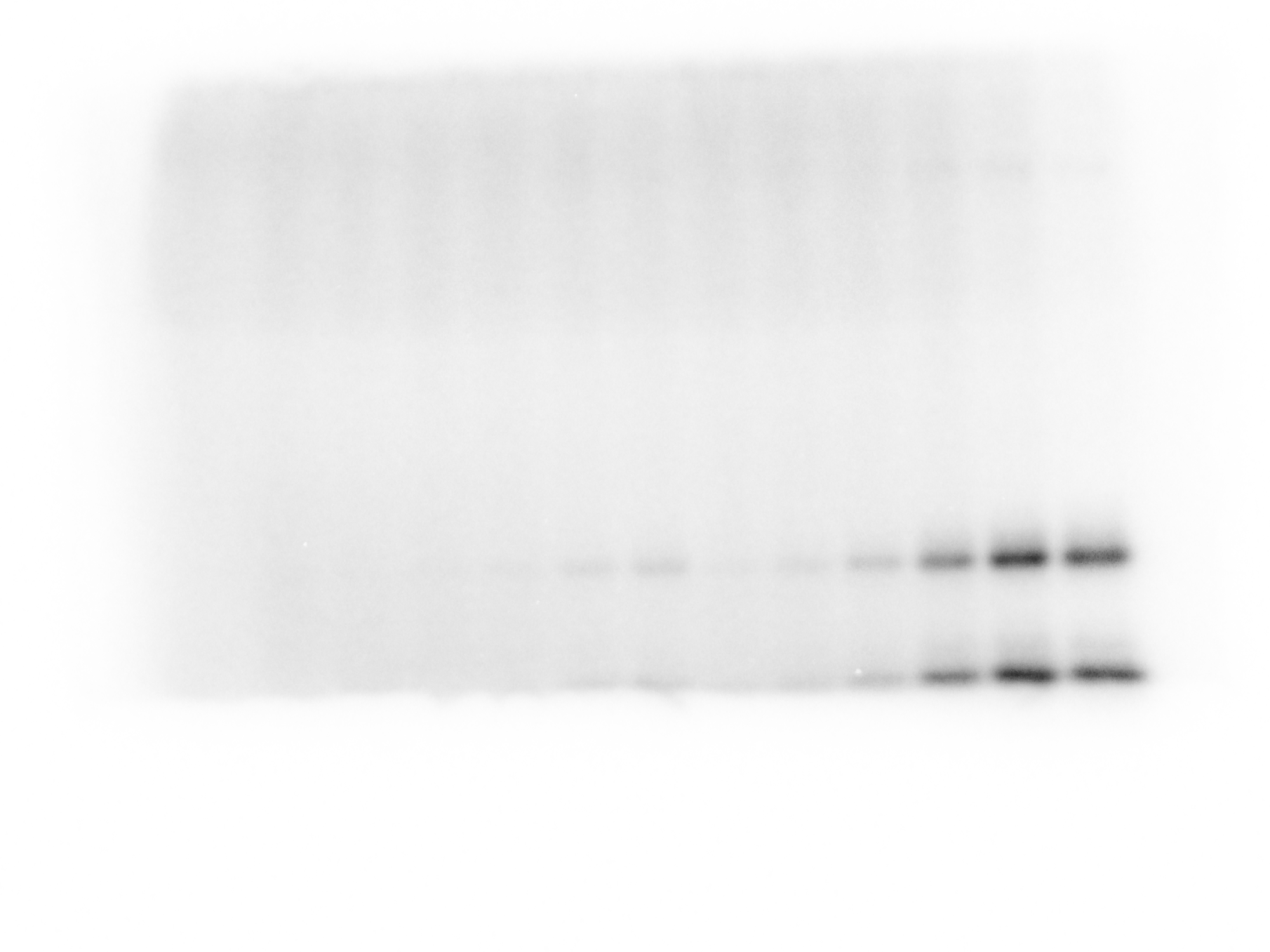

Supplement: Figure 3—source data 3. [file elife-80497-fig3-data3.zip › Figure 3-source data 3/Figure 3A/32P/Replica 3.tif]

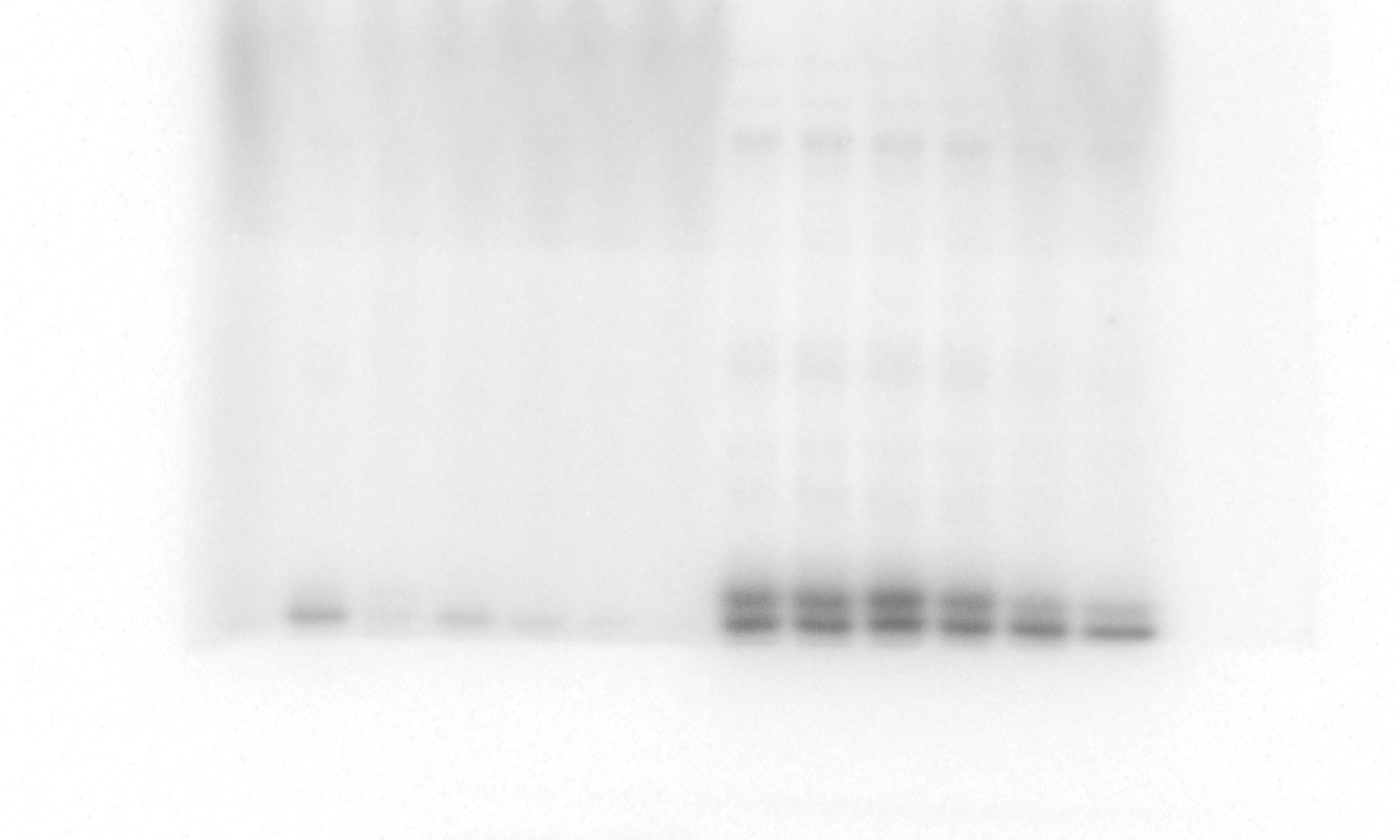

Supplement: Figure 3—source data 3. [file elife-80497-fig3-data3.zip › Figure 3-source data 3/Figure 3A/32P/Replica 1.tif]

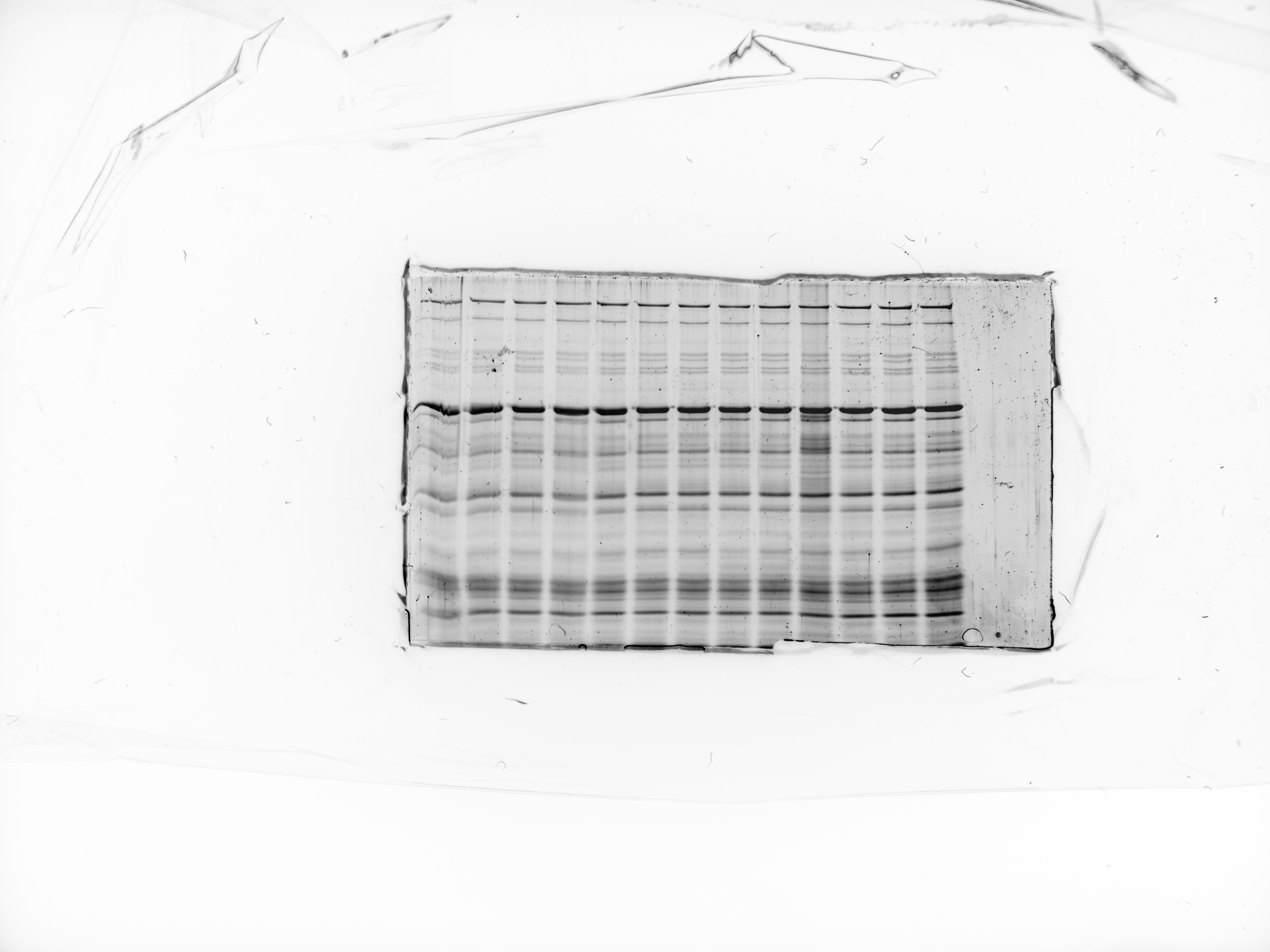

Supplement: Figure 3—source data 3. [file elife-80497-fig3-data3.zip › Figure 3-source data 3/Figure 3A/Sypro/Replica 2.tif]

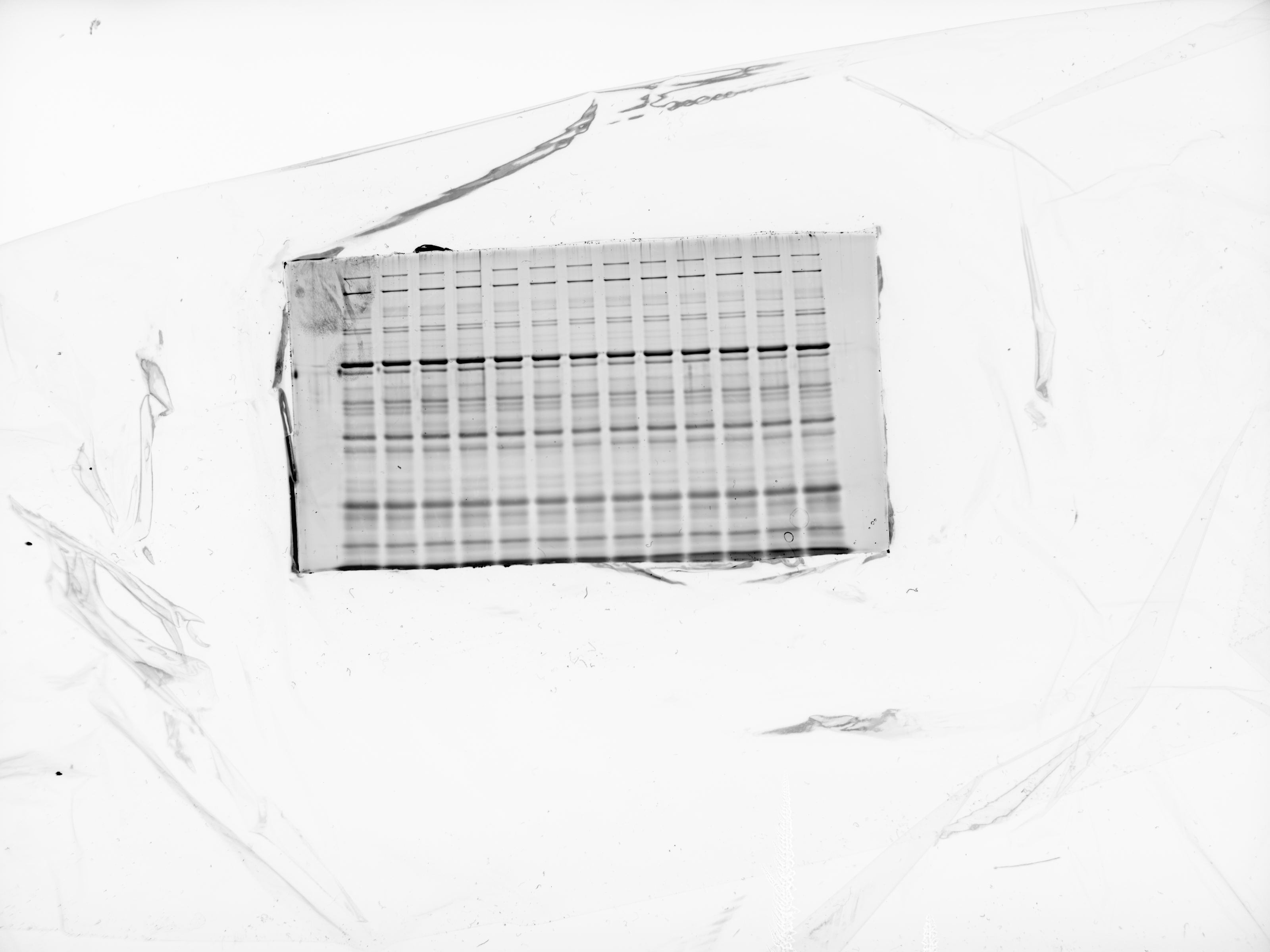

Supplement: Figure 3—source data 3. [file elife-80497-fig3-data3.zip › Figure 3-source data 3/Figure 3A/Sypro/Replica 3.tif]

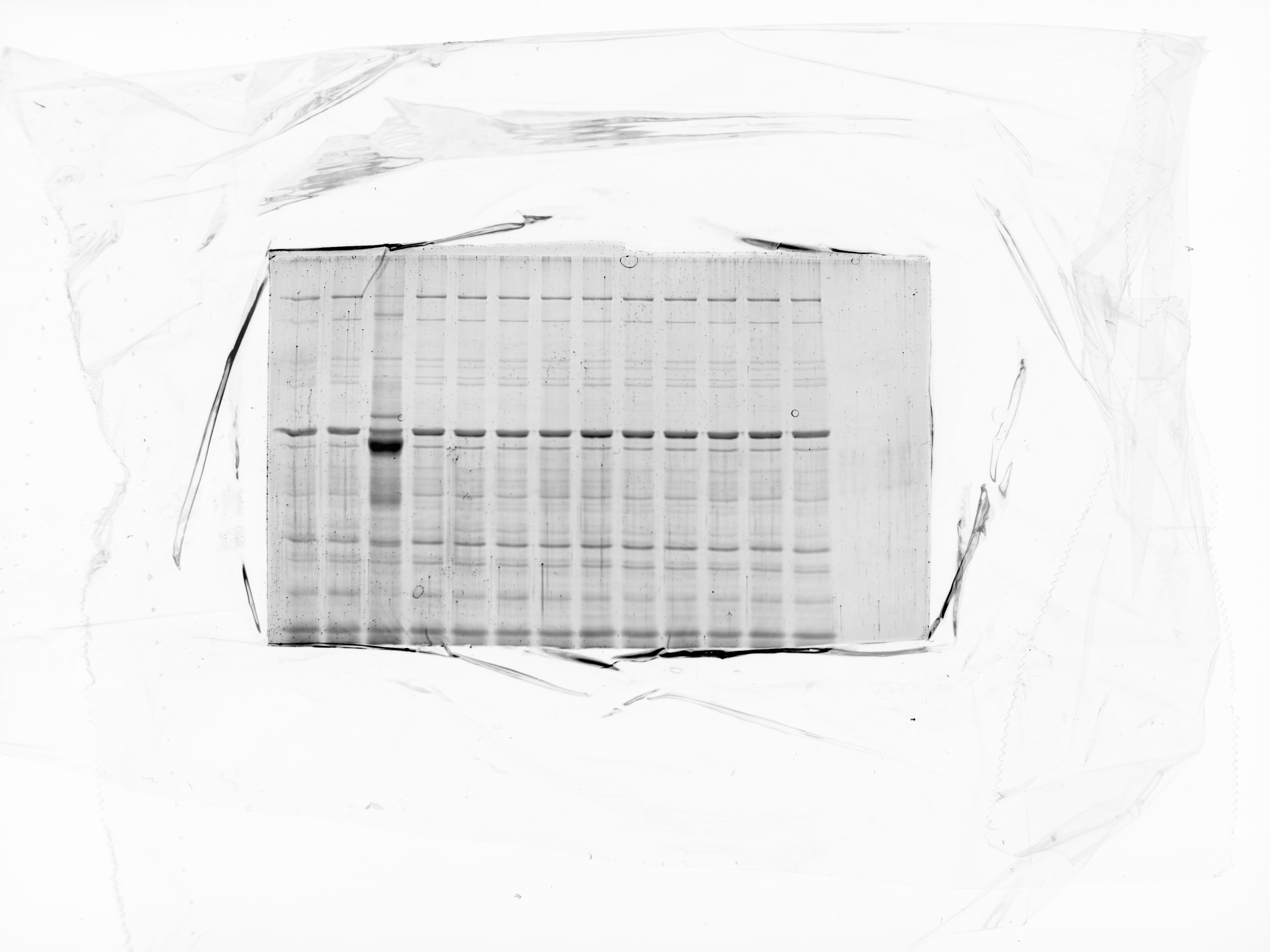

Supplement: Figure 3—source data 3. [file elife-80497-fig3-data3.zip › Figure 3-source data 3/Figure 3A/Sypro/Replica 1.tif]

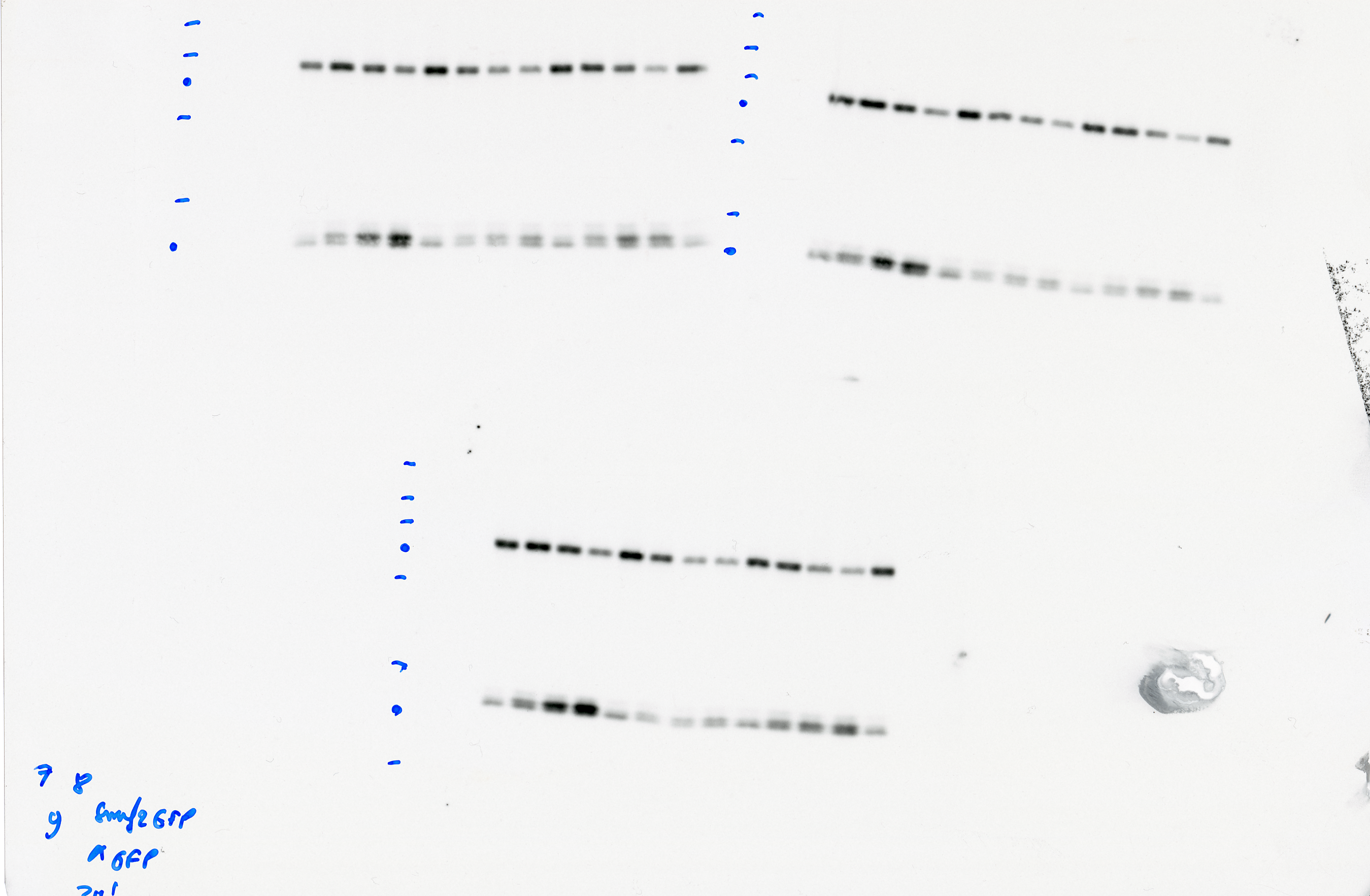

Supplement: Figure 4—source data 3. [file elife-80497-fig4-data3.zip › Figure 4-source data 3/Figure 4D/GFP-Smf2_anti-GFP.tif]

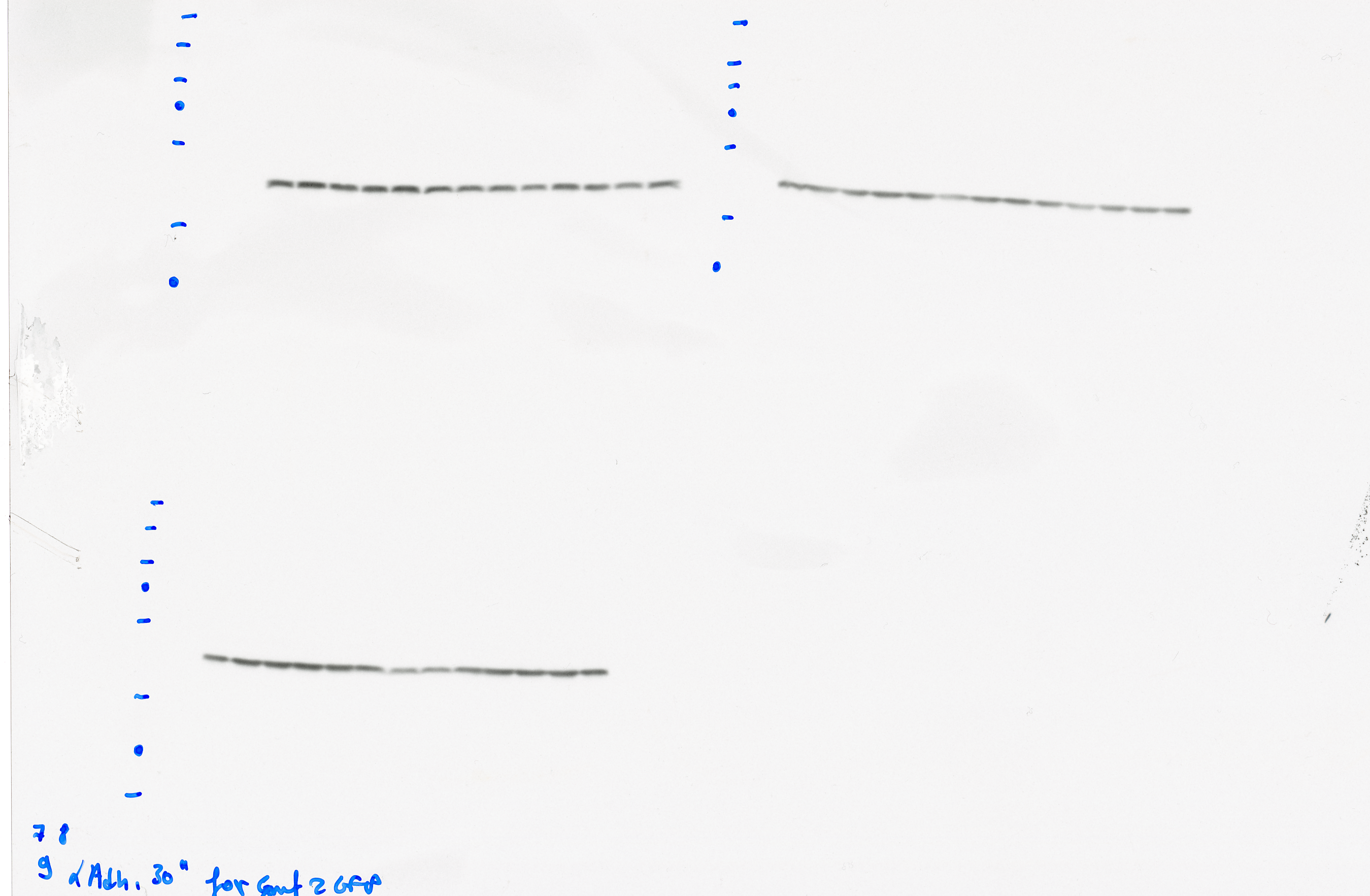

Supplement: Figure 4—source data 3. [file elife-80497-fig4-data3.zip › Figure 4-source data 3/Figure 4D/GFP-Smf2_anti-Adh1.tif]

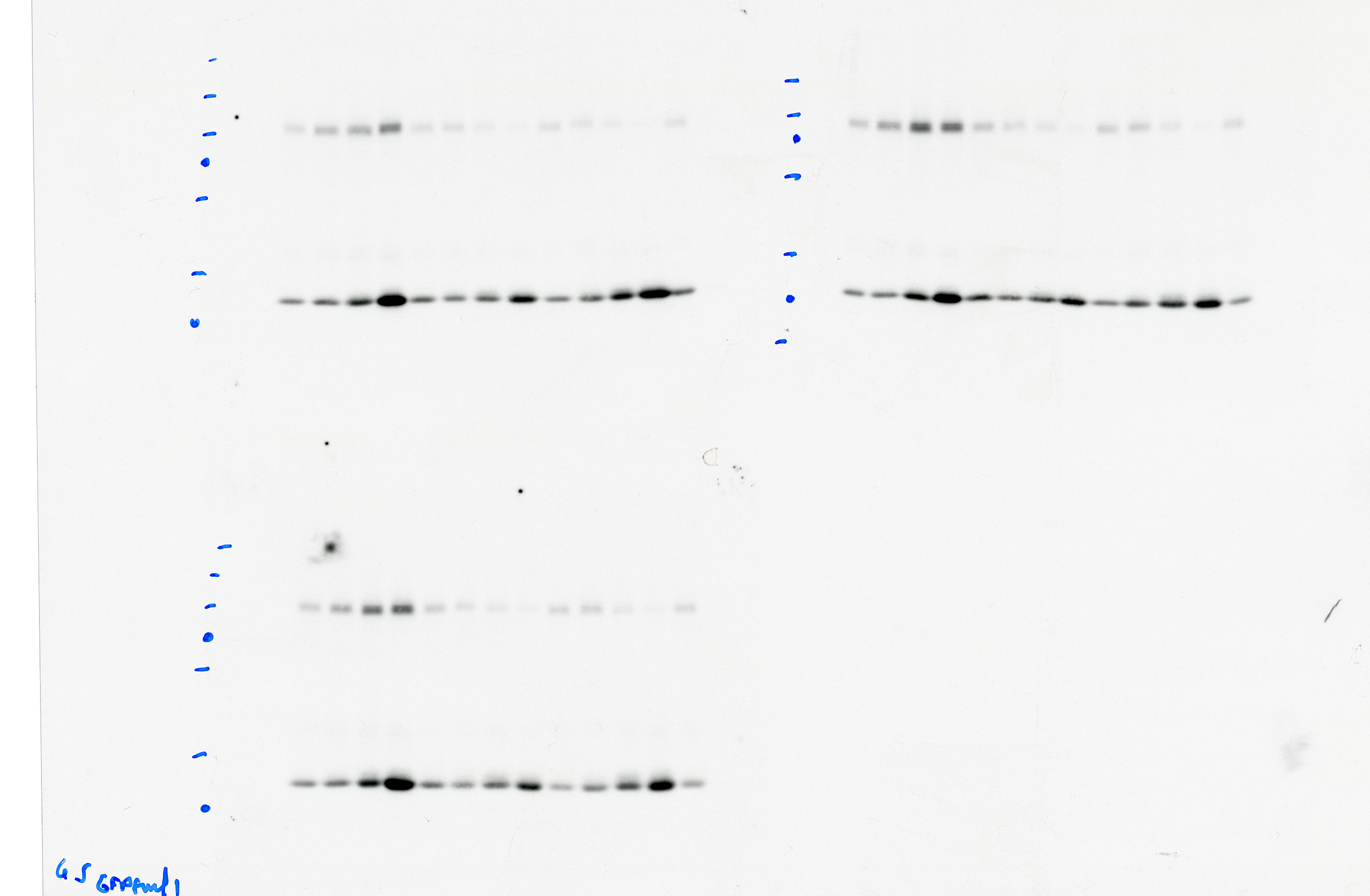

Supplement: Figure 4—source data 3. [file elife-80497-fig4-data3.zip › Figure 4-source data 3/Figure 4A/GFP-Smf1_anti-GFP.tif]

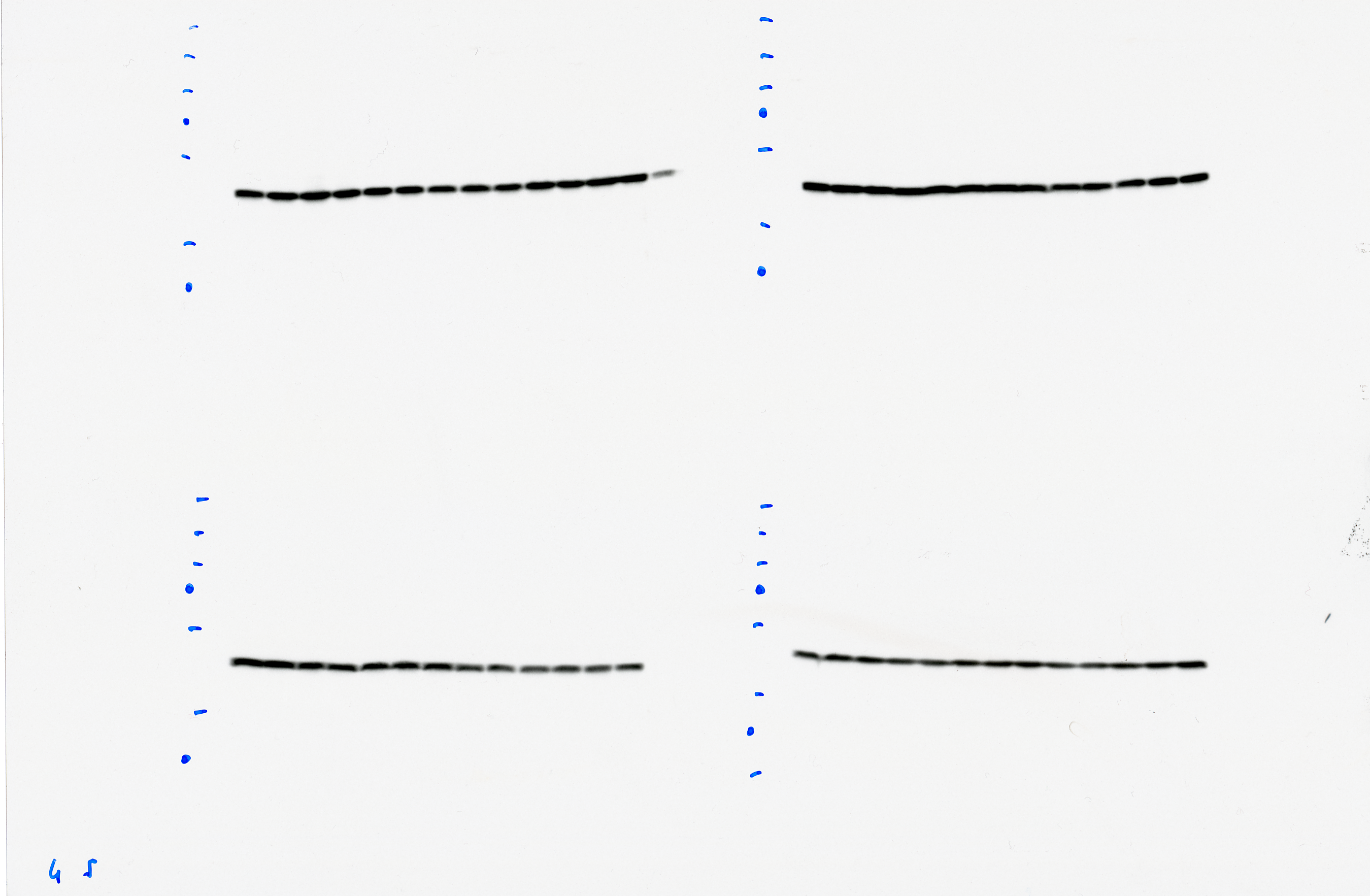

Supplement: Figure 4—source data 3. [file elife-80497-fig4-data3.zip › Figure 4-source data 3/Figure 4A/GFP-Smf1_anti-Adh1.tif]

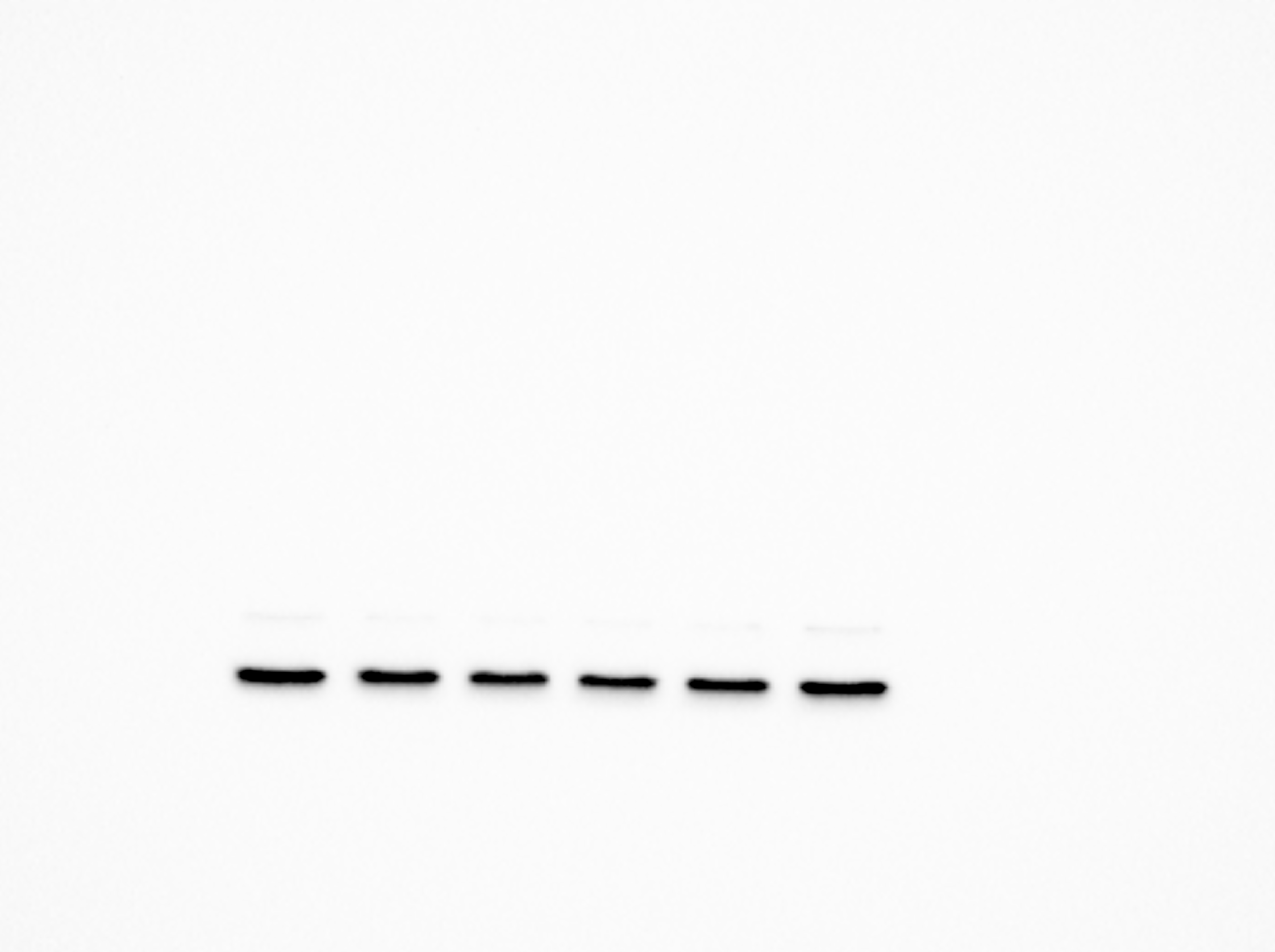

Supplement: Figure 5—source data 3. [file elife-80497-fig5-data3.zip › Figure 5-source data 3/Figure 5D-E/Figure 5 D-E - actin.tif]

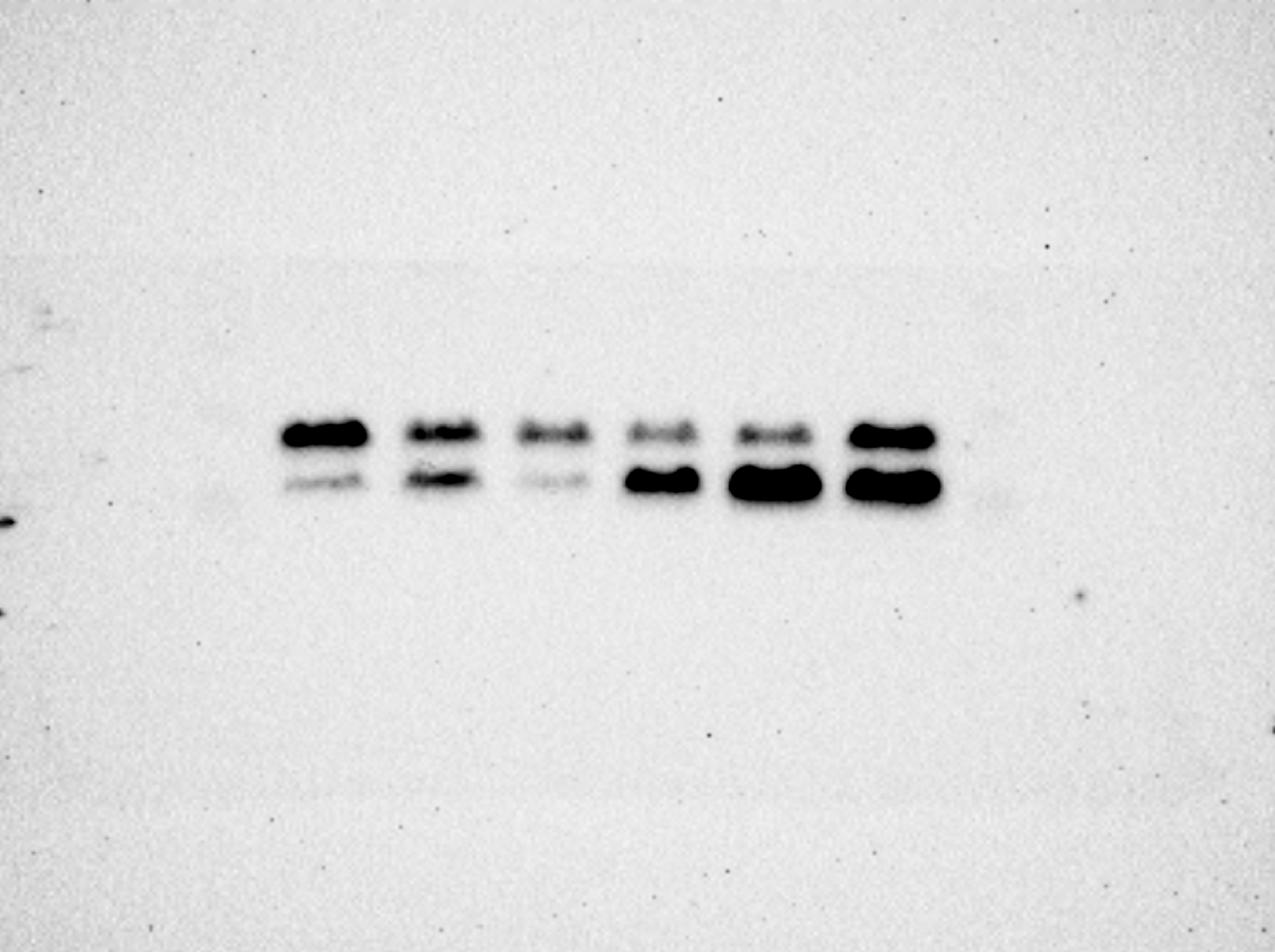

Supplement: Figure 5—source data 3. [file elife-80497-fig5-data3.zip › Figure 5-source data 3/Figure 5D-E/Figure 5 D-E - MAP1LC3.tif]

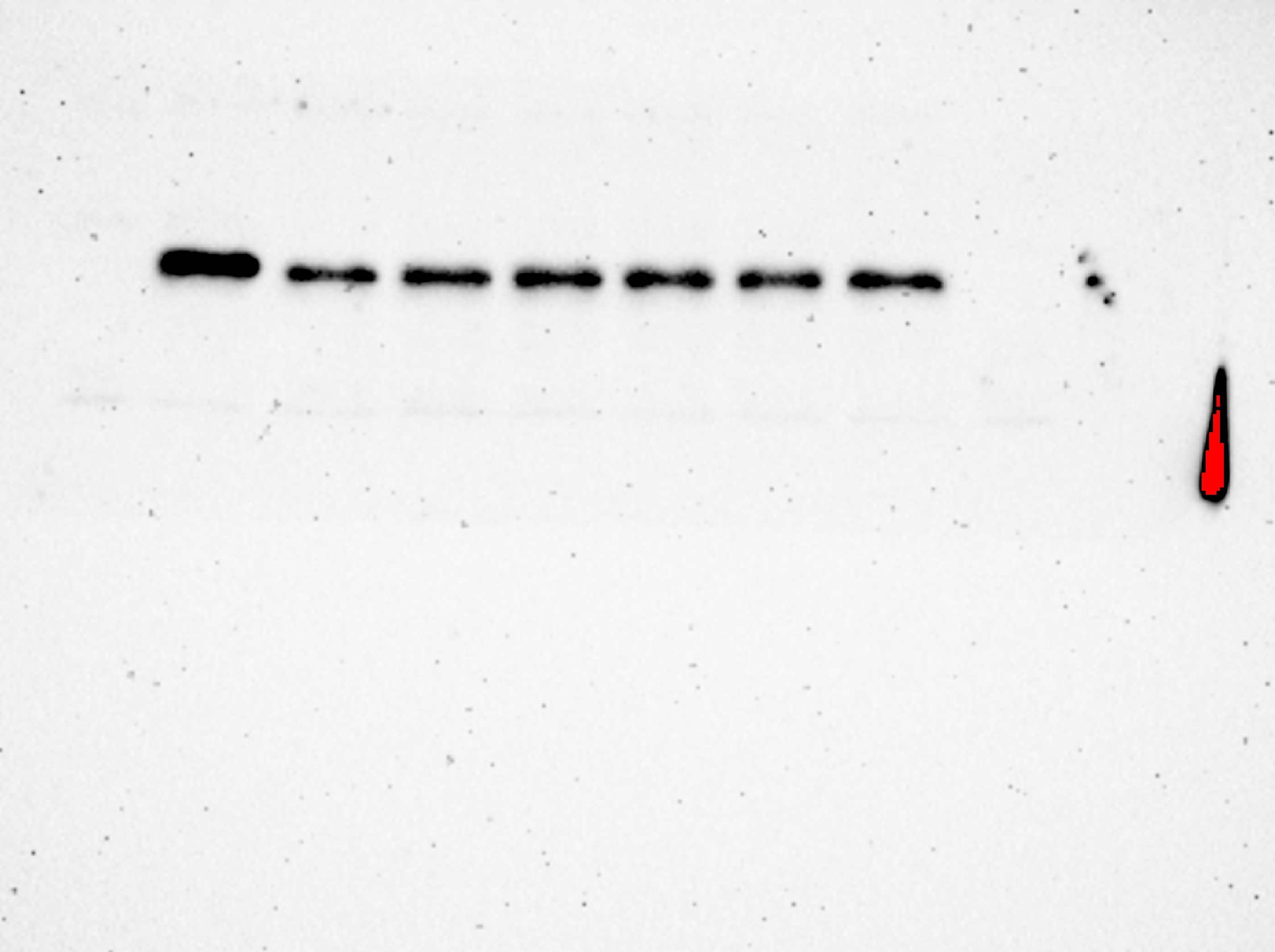

Supplement: Figure 5—source data 3. [file elife-80497-fig5-data3.zip › Figure 5-source data 3/Figure 5A/Figure 5 B - RPS6.tif]

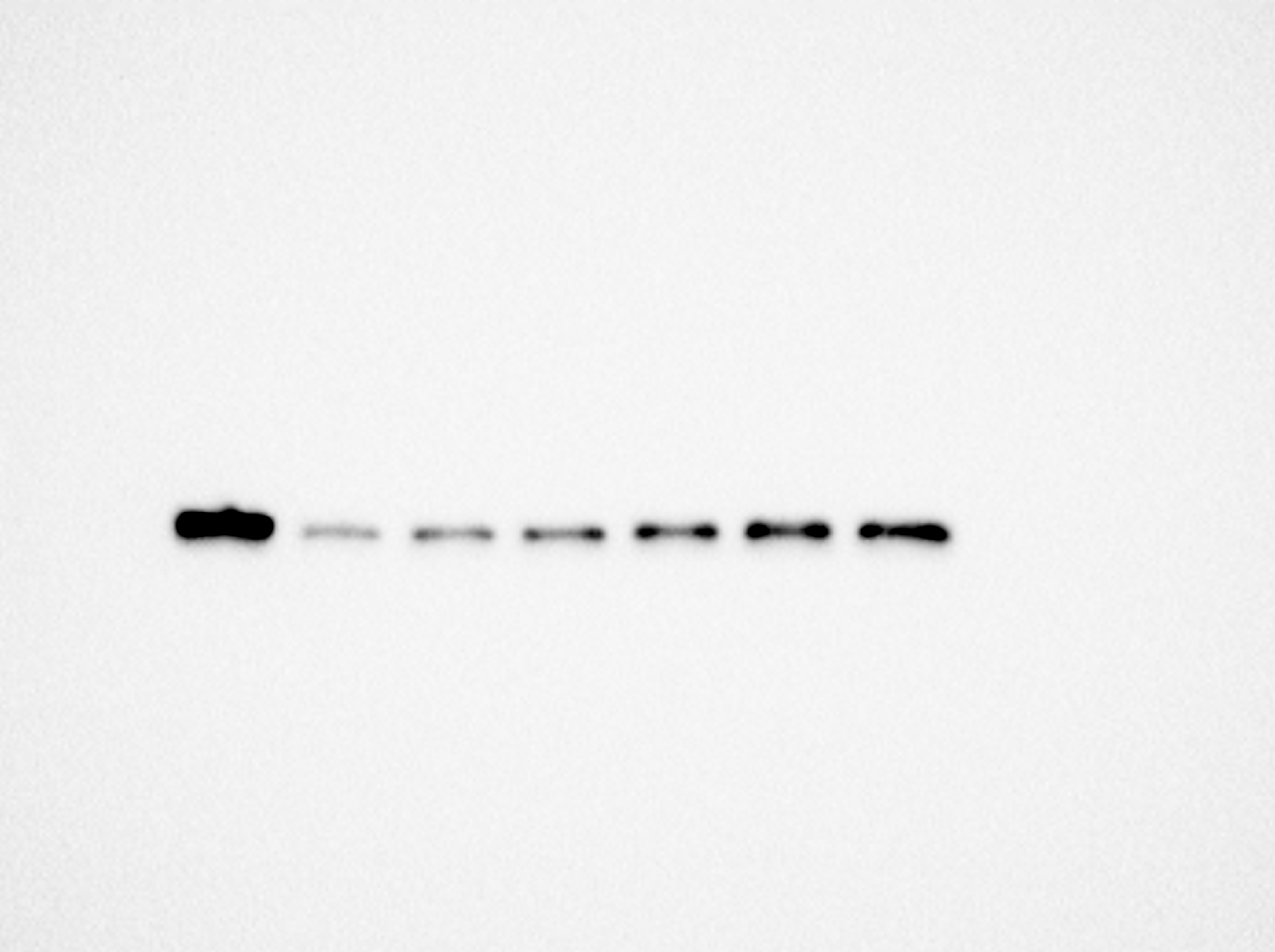

Supplement: Figure 5—source data 3. [file elife-80497-fig5-data3.zip › Figure 5-source data 3/Figure 5A/Figure 5 A - P(Ser235-236) -RPS6.tif]

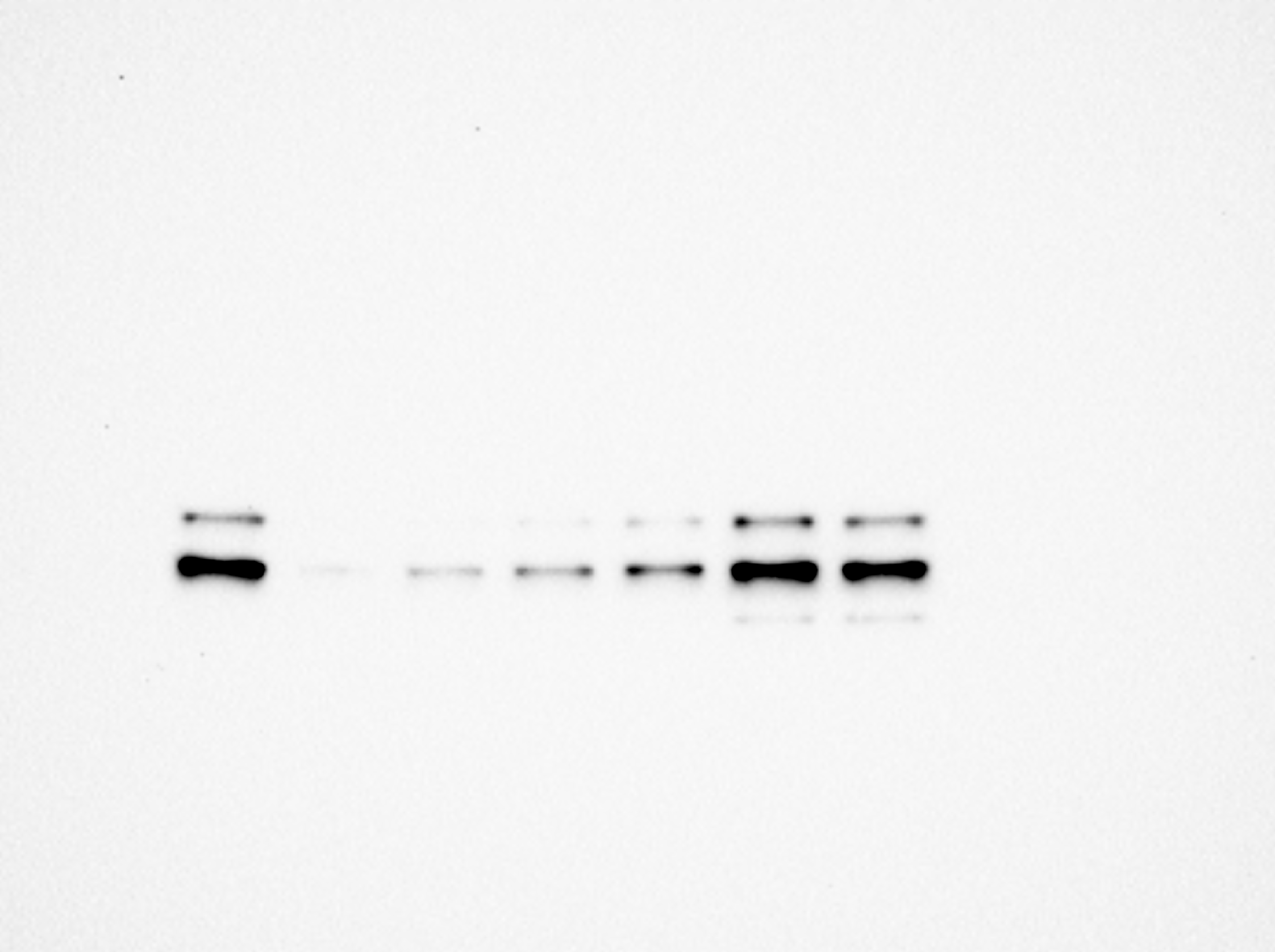

Supplement: Figure 5—source data 3. [file elife-80497-fig5-data3.zip › Figure 5-source data 3/Figure 5A/Figure 5 A - P(Thr389)-RPS6KB.tif]

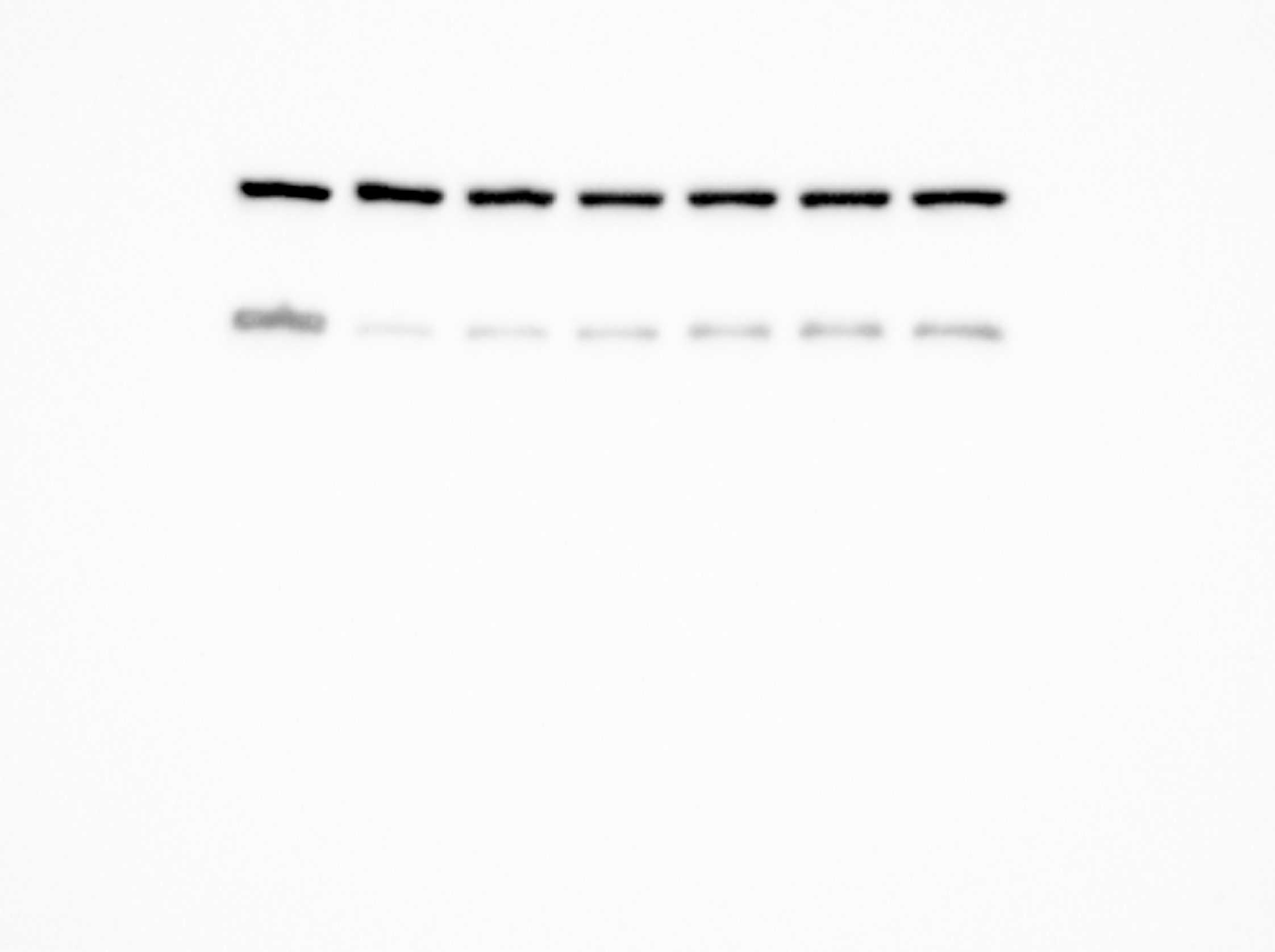

Supplement: Figure 5—source data 3. [file elife-80497-fig5-data3.zip › Figure 5-source data 3/Figure 5A/Figure 5 A - actin.tif]

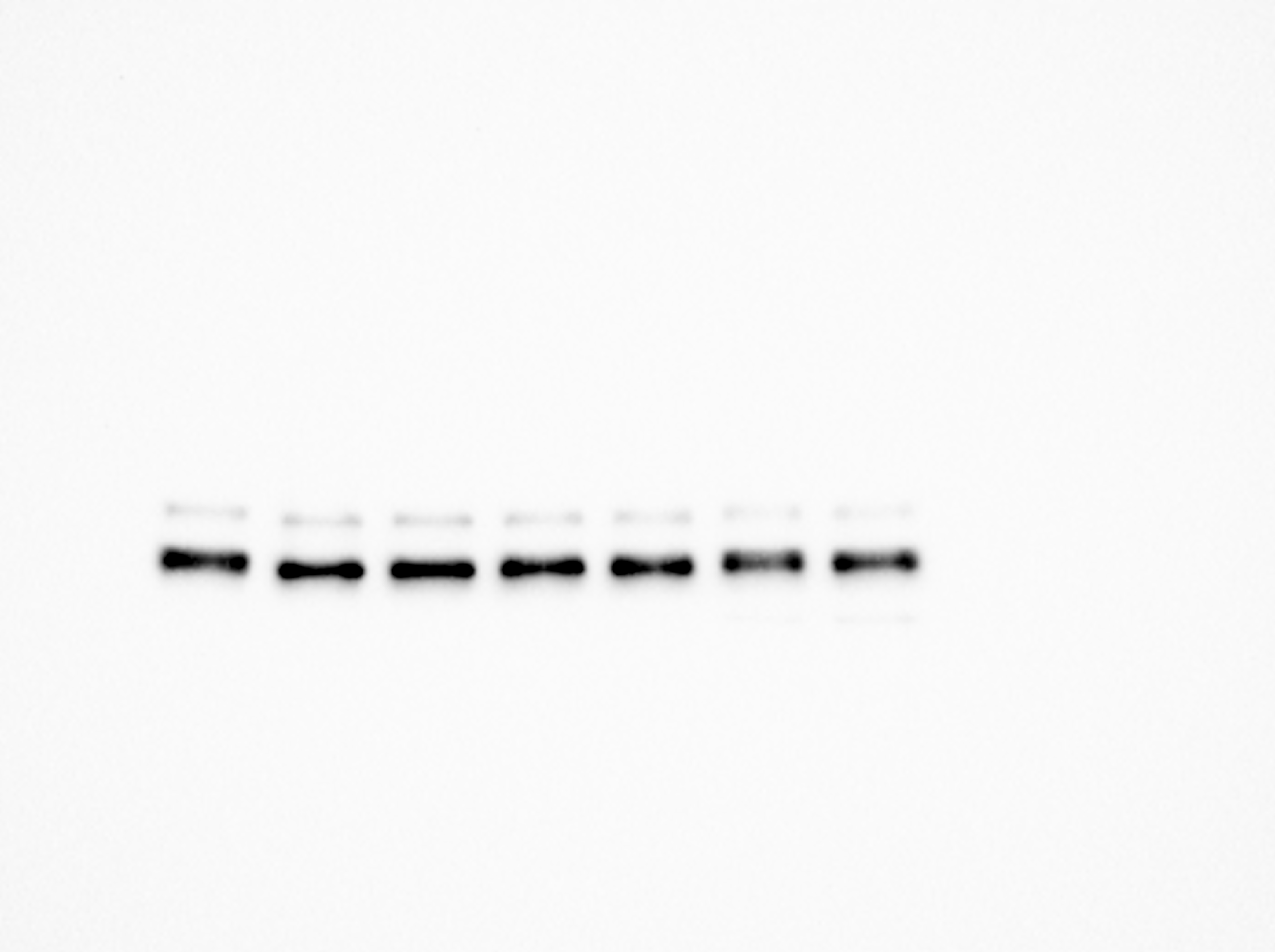

Supplement: Figure 5—source data 3. [file elife-80497-fig5-data3.zip › Figure 5-source data 3/Figure 5A/Figure 5 A - RPS6KB.tif]

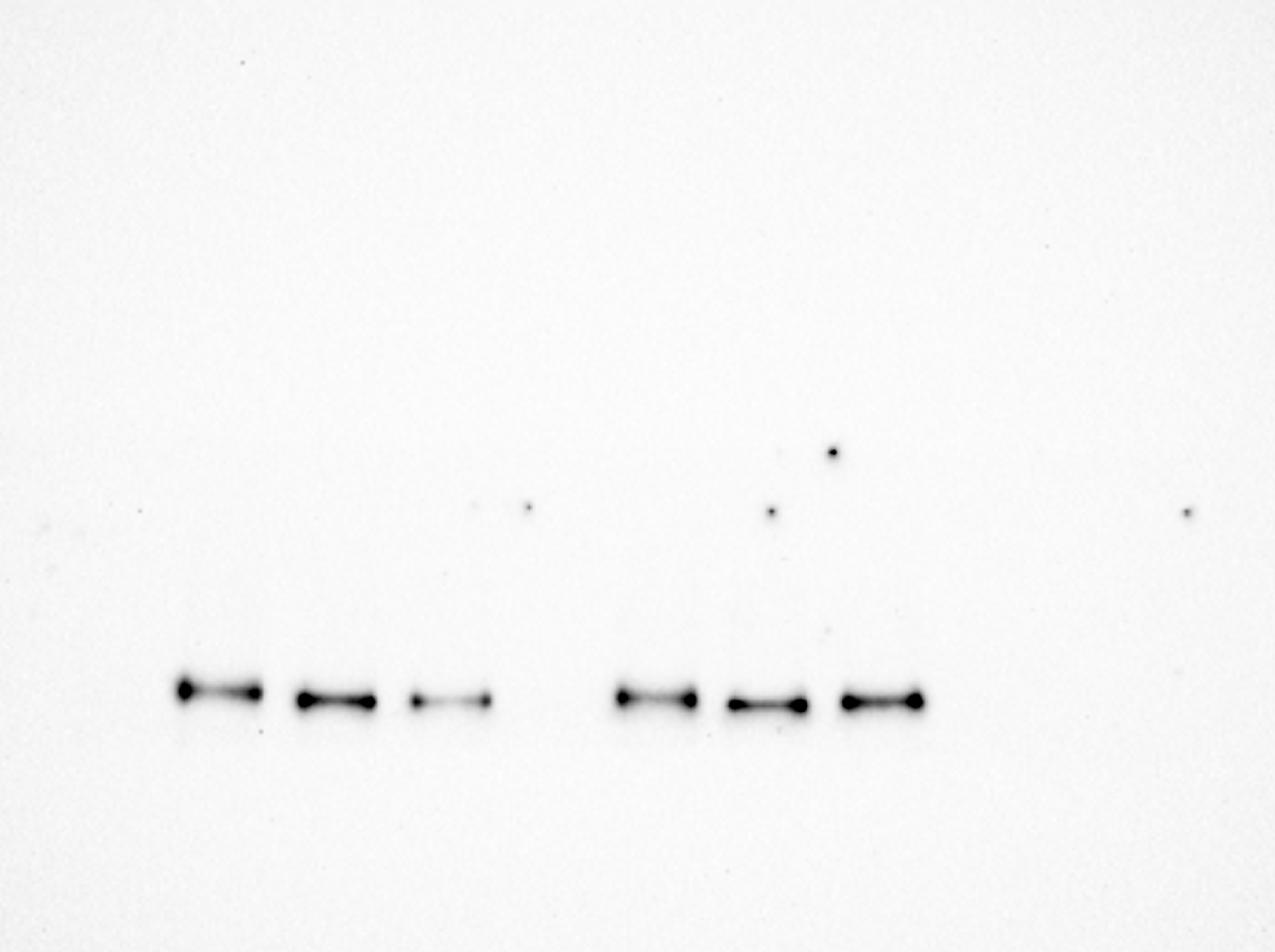

Supplement: Figure 5—source data 3. [file elife-80497-fig5-data3.zip › Figure 5-source data 3/Figure 5C/Figure 5 C - ULK1.tif]

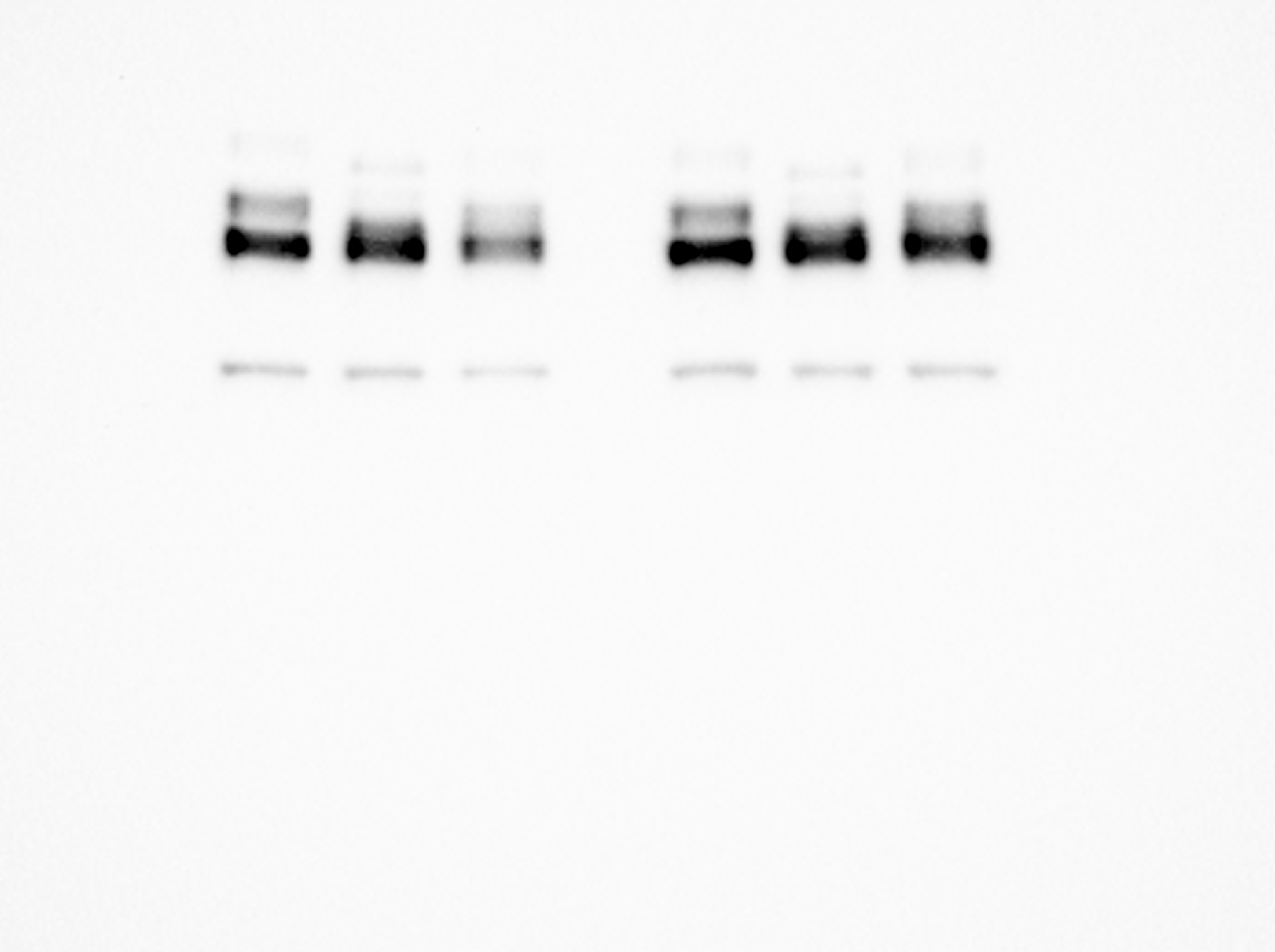

Supplement: Figure 5—source data 3. [file elife-80497-fig5-data3.zip › Figure 5-source data 3/Figure 5C/Figure 5 C - AKT1.tif]

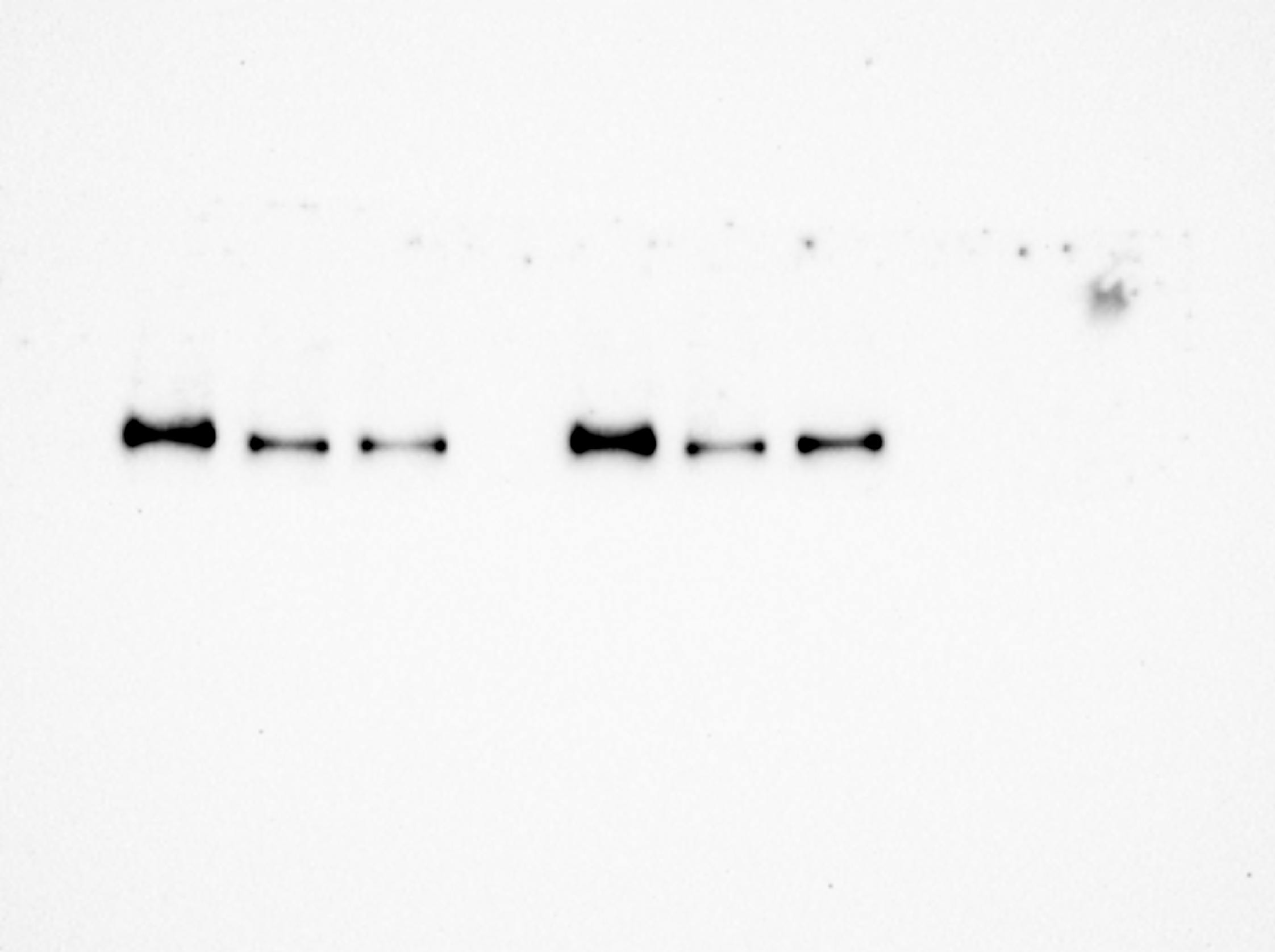

Supplement: Figure 5—source data 3. [file elife-80497-fig5-data3.zip › Figure 5-source data 3/Figure 5C/Figure 5 C - P(Ser757)-ULK1.tif]

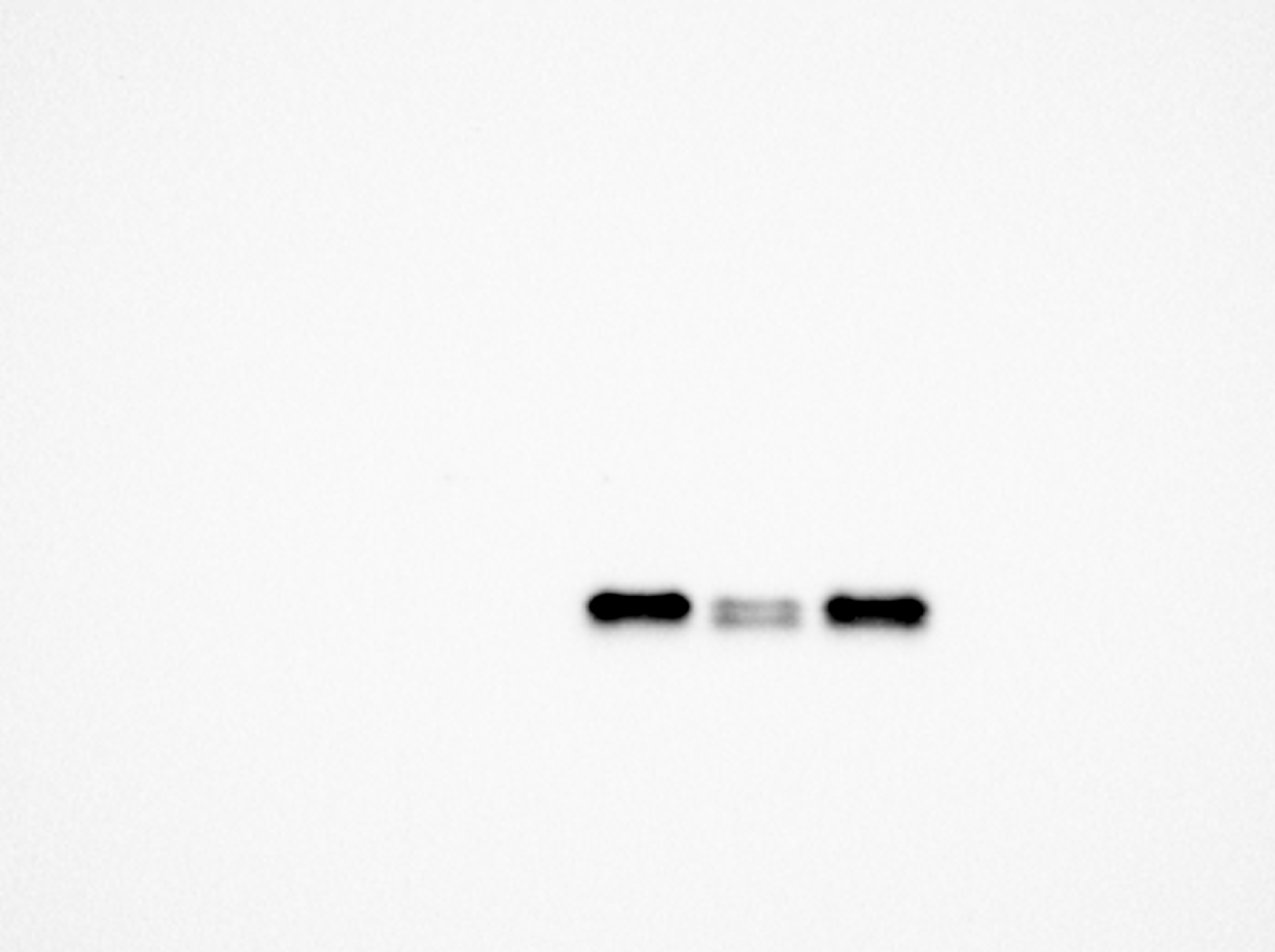

Supplement: Figure 5—source data 3. [file elife-80497-fig5-data3.zip › Figure 5-source data 3/Figure 5C/Figure 5 C - P(Ser65)-EIF4EBP1.tif]

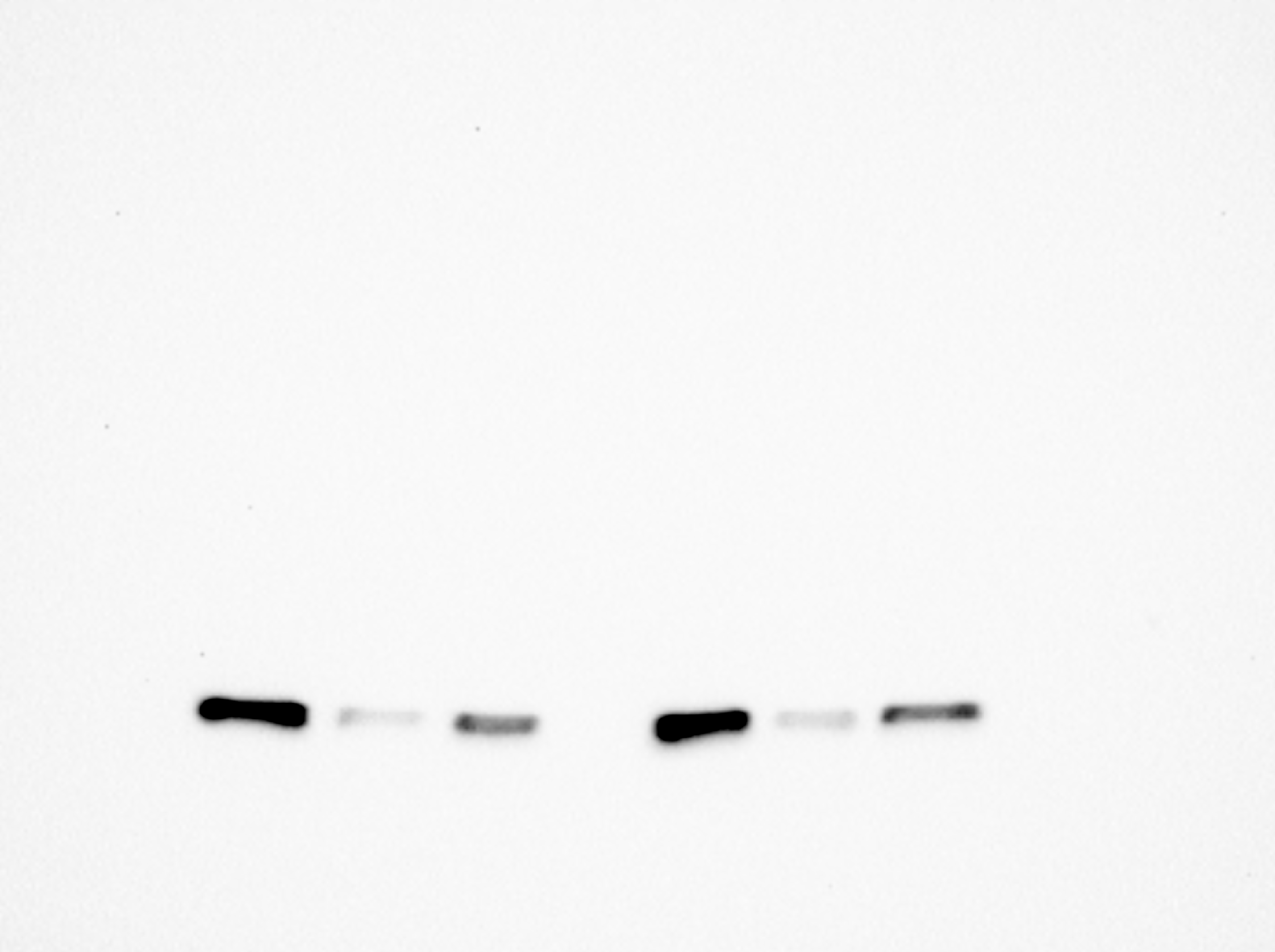

Supplement: Figure 5—source data 3. [file elife-80497-fig5-data3.zip › Figure 5-source data 3/Figure 5C/Figure 5 C - P(Ser235-236) -RPS6.tif]

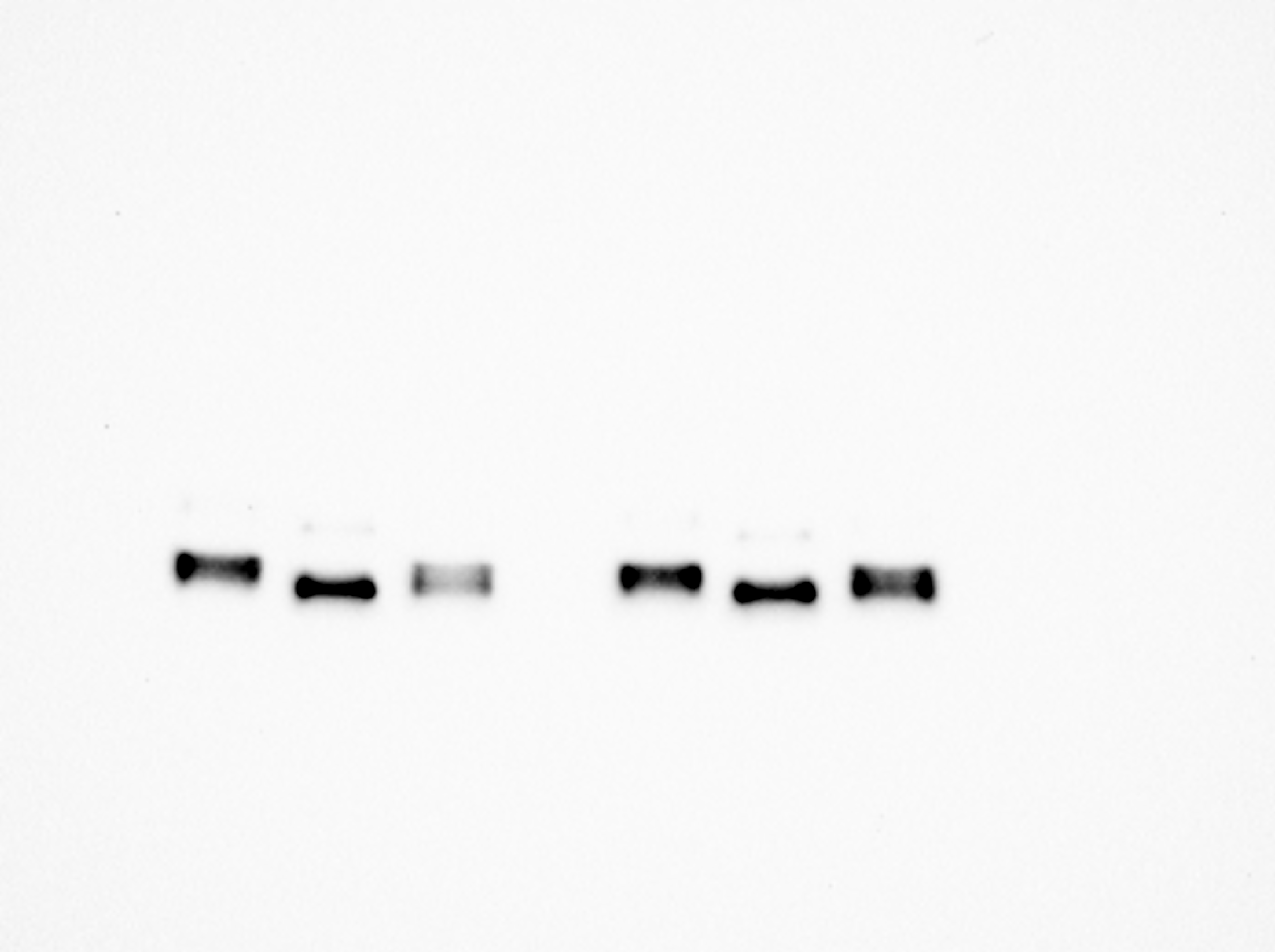

Supplement: Figure 5—source data 3. [file elife-80497-fig5-data3.zip › Figure 5-source data 3/Figure 5C/Figure 5 C - RPS6KB.tif]

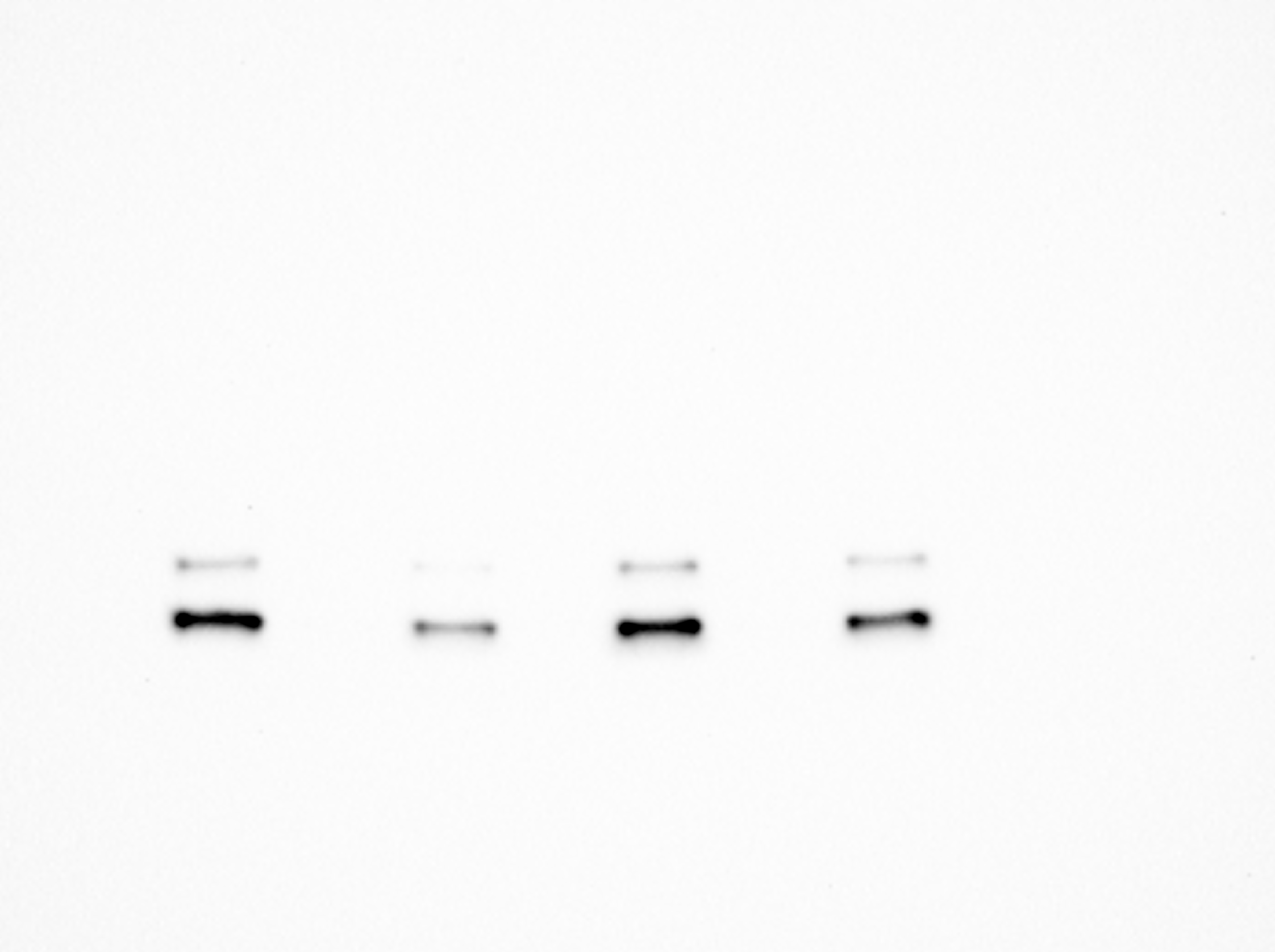

Supplement: Figure 5—source data 3. [file elife-80497-fig5-data3.zip › Figure 5-source data 3/Figure 5C/Figure 5 C - P(Thr389)-RPS6KB.tif]

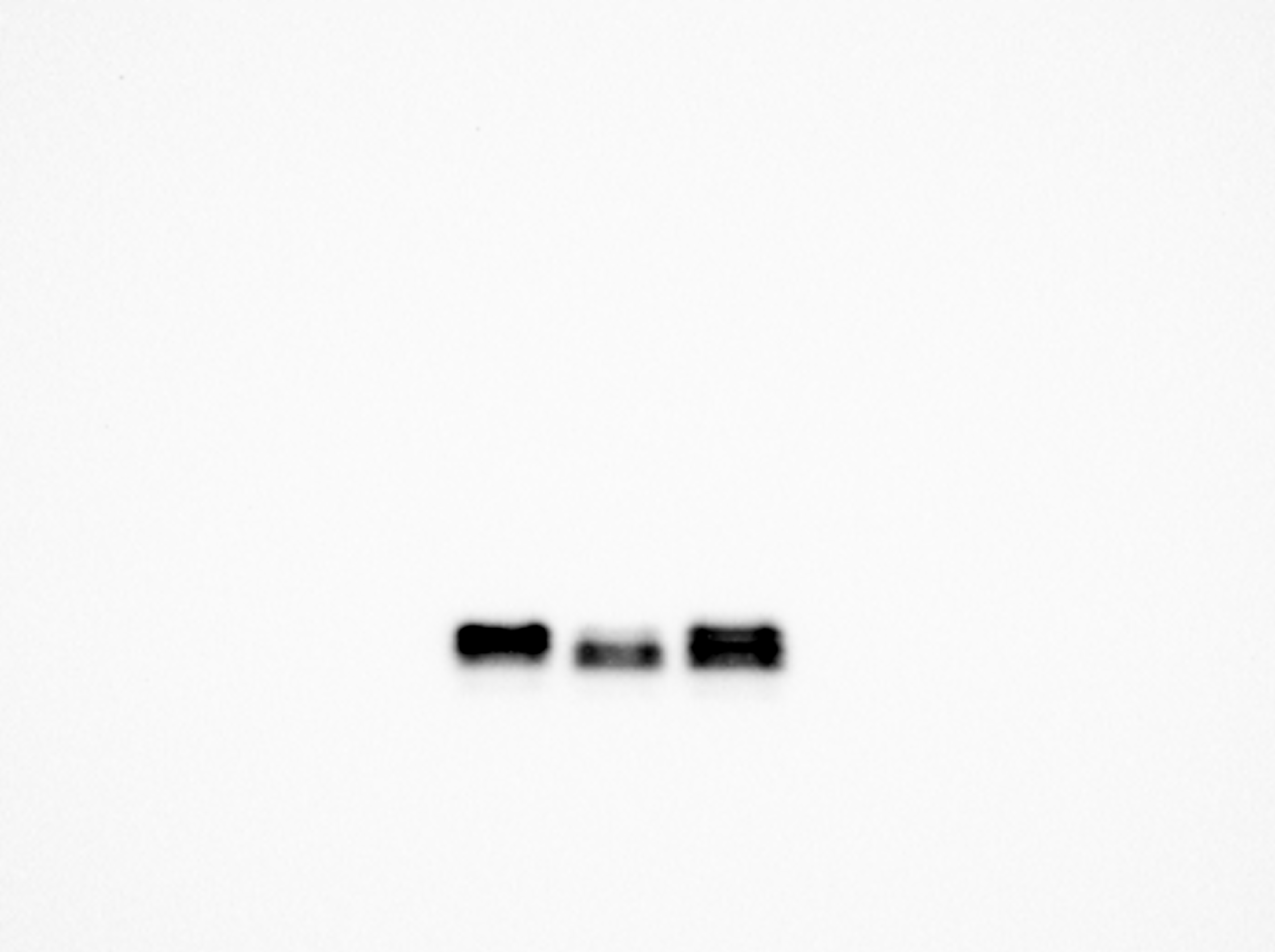

Supplement: Figure 5—source data 3. [file elife-80497-fig5-data3.zip › Figure 5-source data 3/Figure 5C/Figure 5 C - P(Thr3746)-EIF4EBP1.tif]

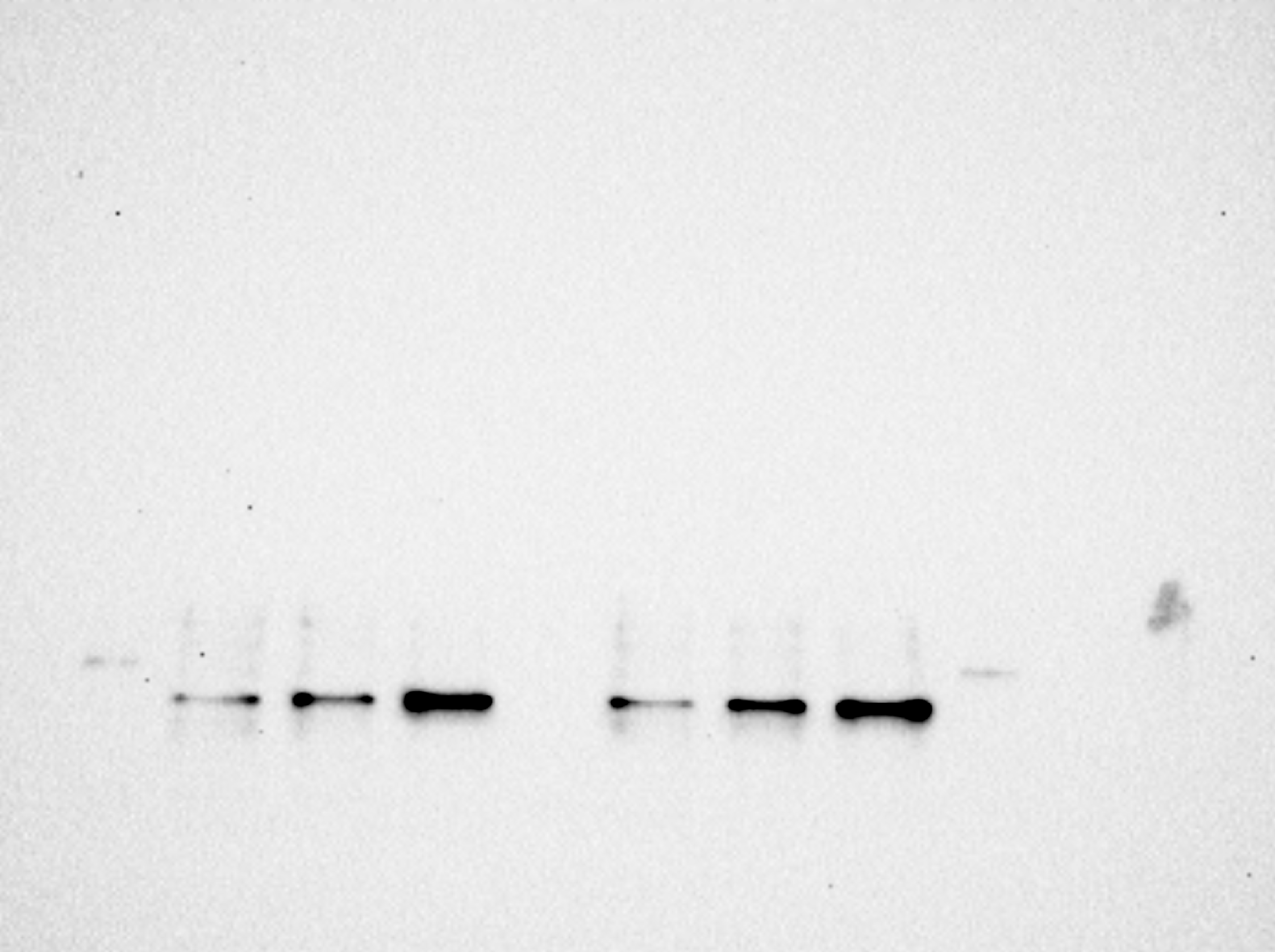

Supplement: Figure 5—source data 3. [file elife-80497-fig5-data3.zip › Figure 5-source data 3/Figure 5C/Figure 5 C - P(Thr308)-AKT1.tif]

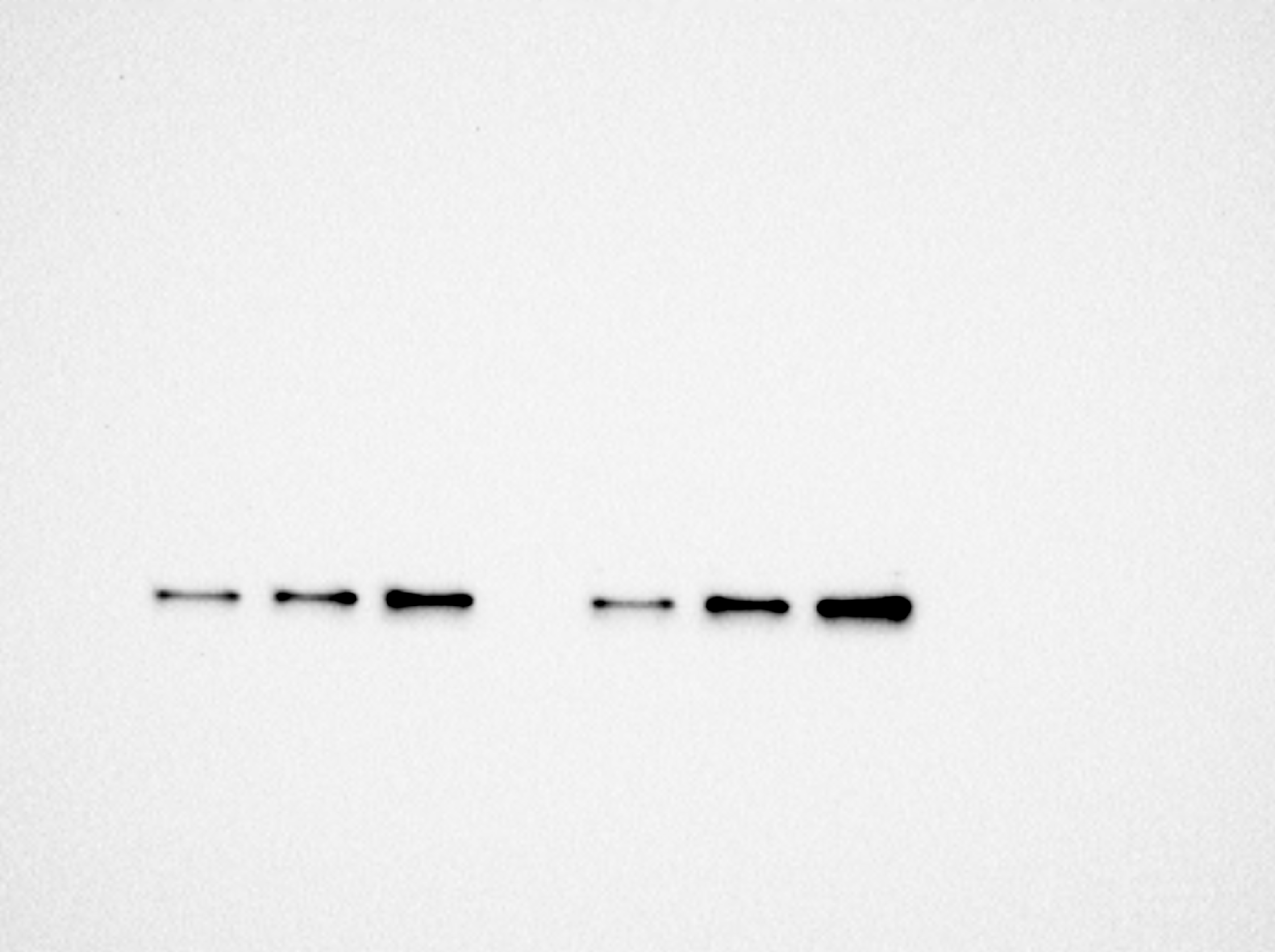

Supplement: Figure 5—source data 3. [file elife-80497-fig5-data3.zip › Figure 5-source data 3/Figure 5C/Figure 5 C - P(Ser473)-AKT1.tif]

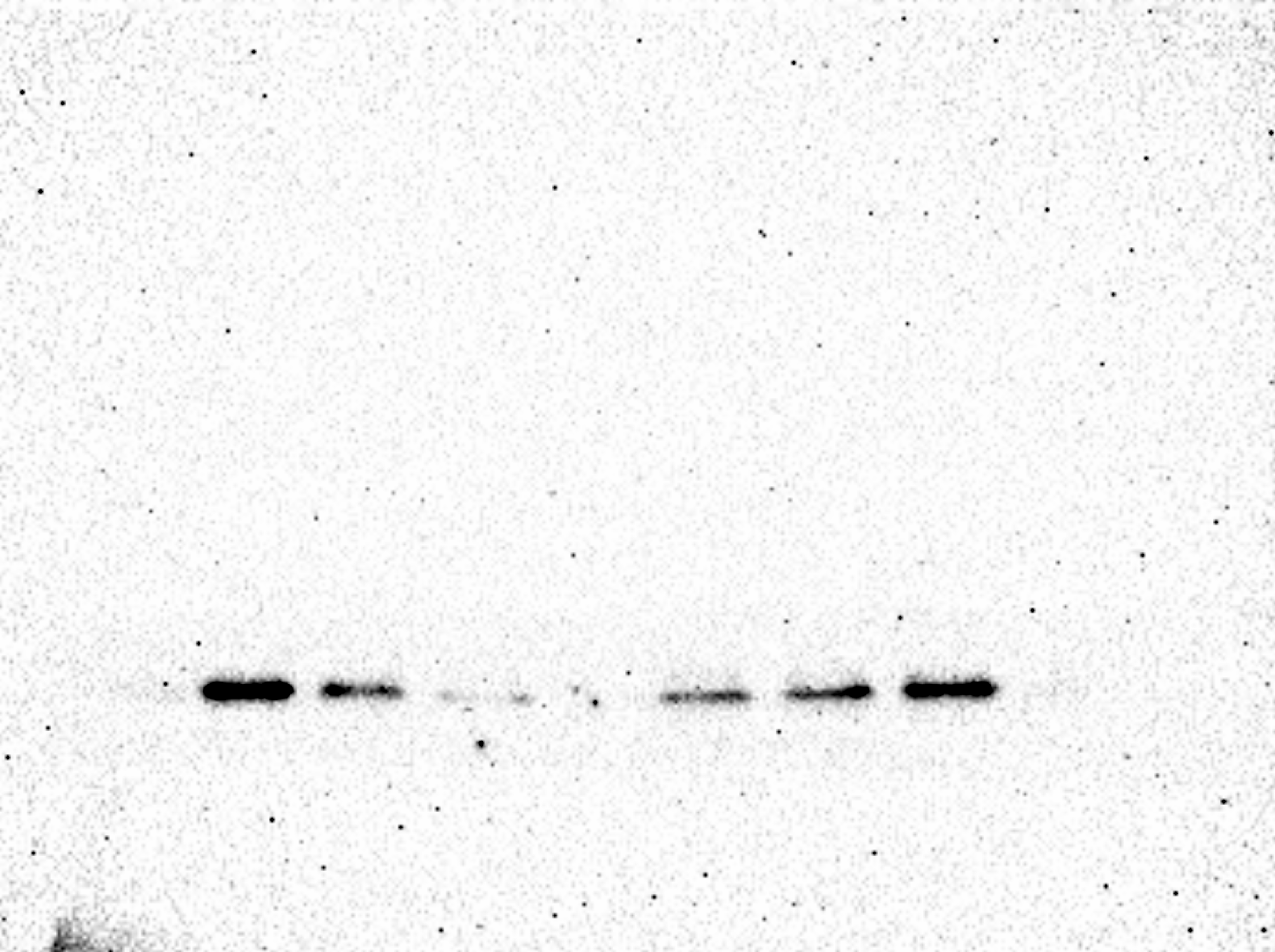

Supplement: Figure 5—source data 3. [file elife-80497-fig5-data3.zip › Figure 5-source data 3/Figure 5C/Figure 5 C - RPS6.tif]

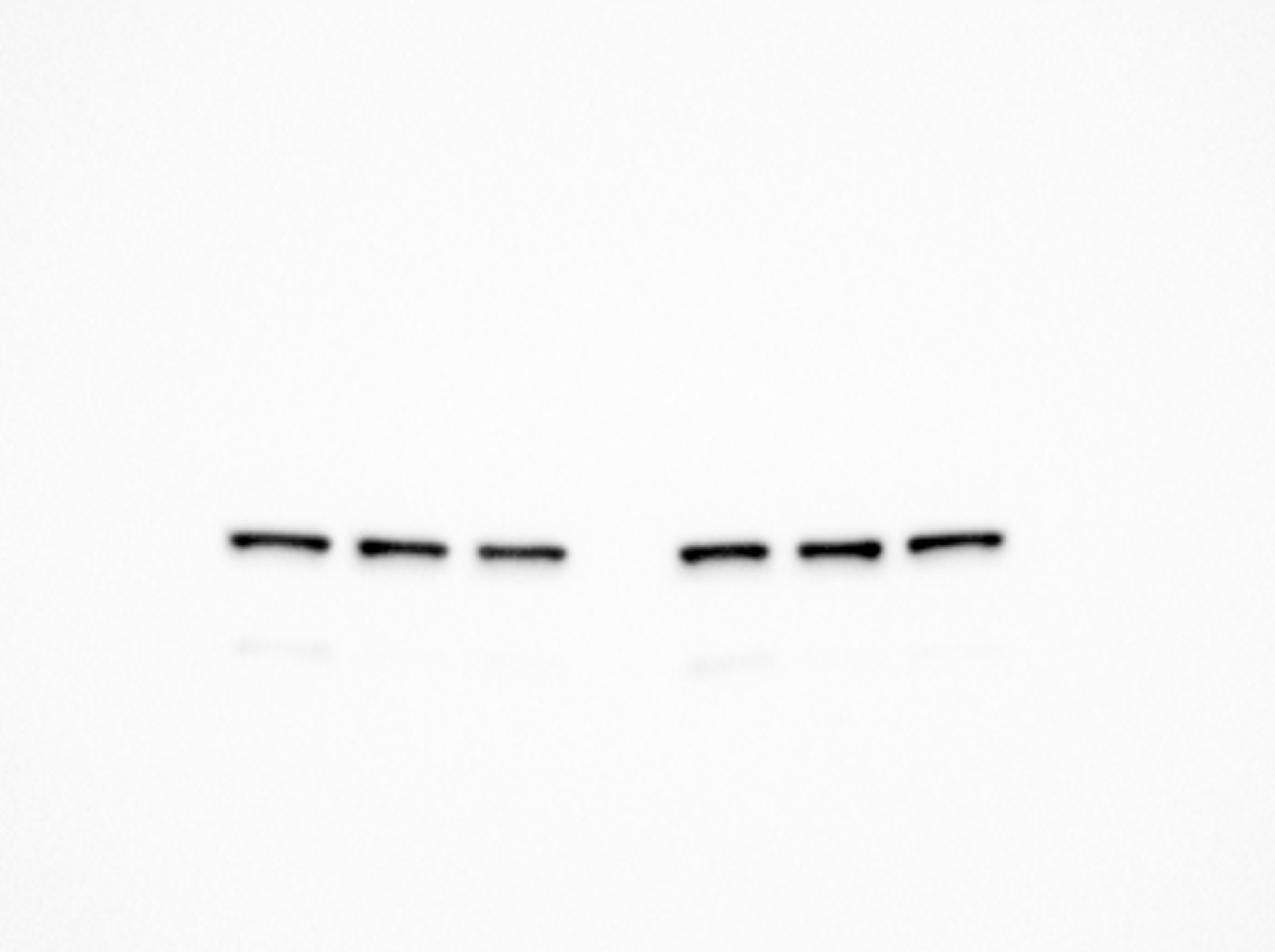

Supplement: Figure 5—source data 3. [file elife-80497-fig5-data3.zip › Figure 5-source data 3/Figure 5C/Figure 5 C - actin.tif]

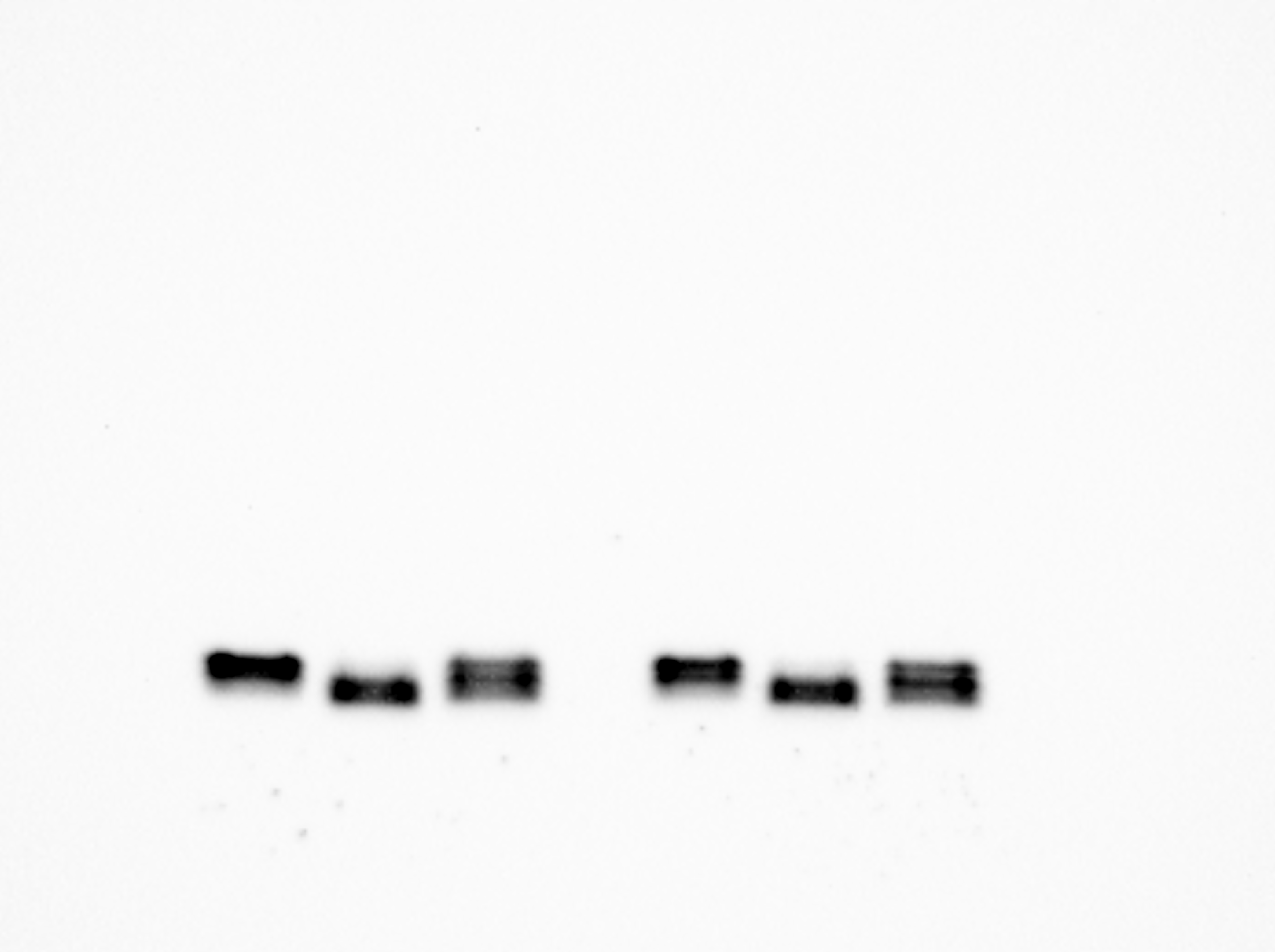

Supplement: Figure 5—source data 3. [file elife-80497-fig5-data3.zip › Figure 5-source data 3/Figure 5C/Figure 5 C - EIF4EBP1.tif]

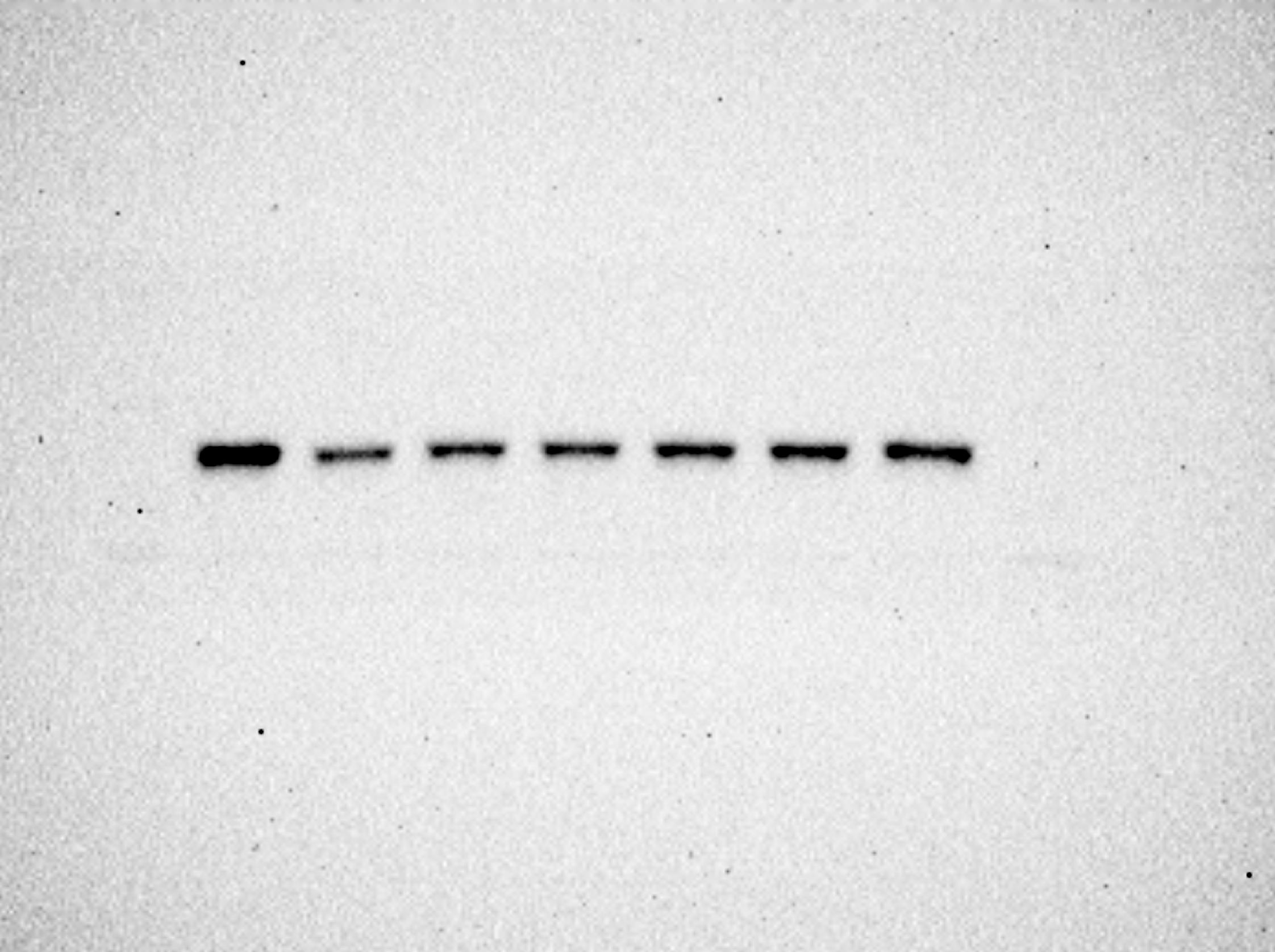

Supplement: Figure 5—source data 3. [file elife-80497-fig5-data3.zip › Figure 5-source data 3/Figure 5B/Figure 5 B - RPS6.tif]

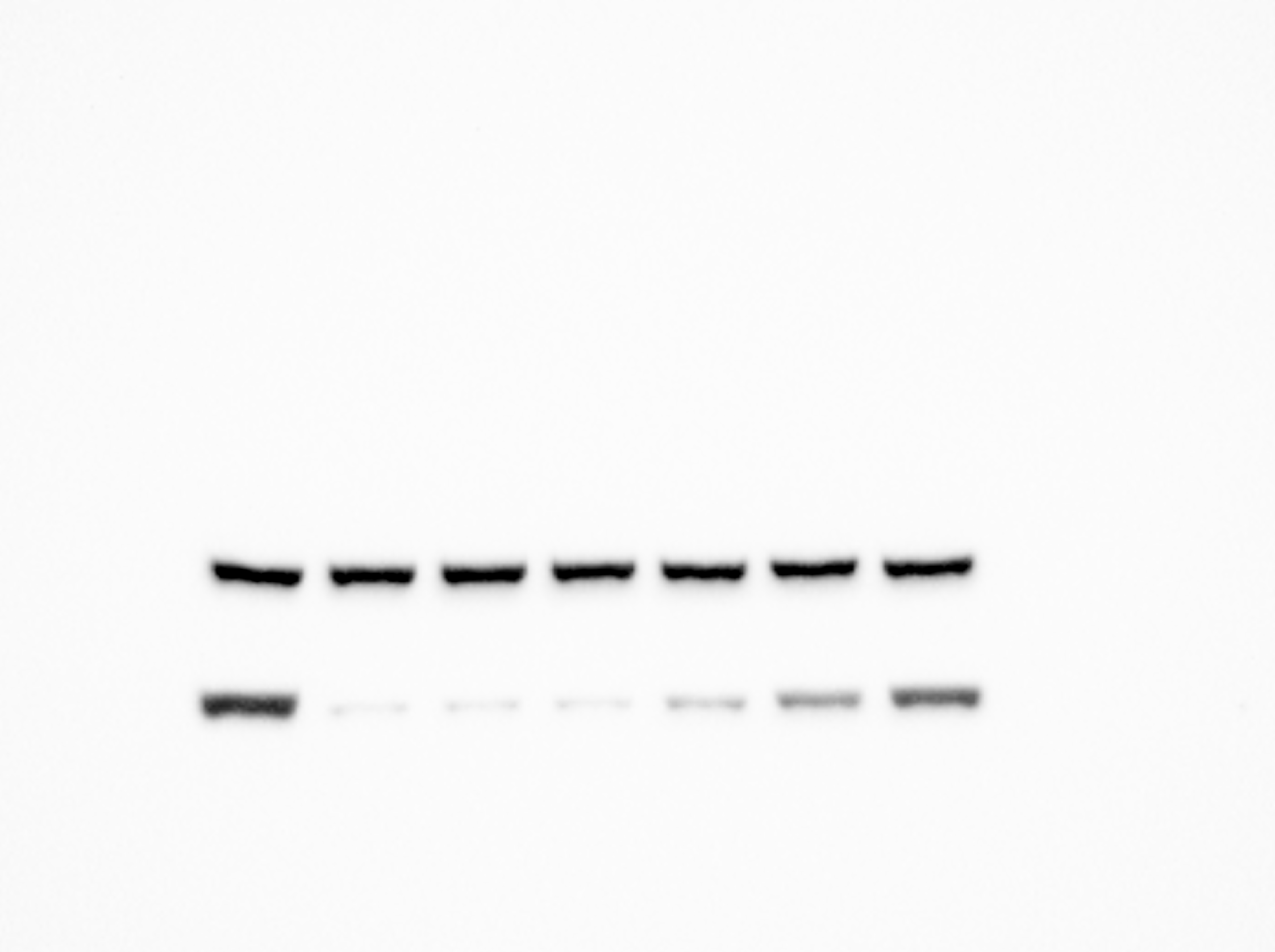

Supplement: Figure 5—source data 3. [file elife-80497-fig5-data3.zip › Figure 5-source data 3/Figure 5B/Figure 5 B - actin.tif]

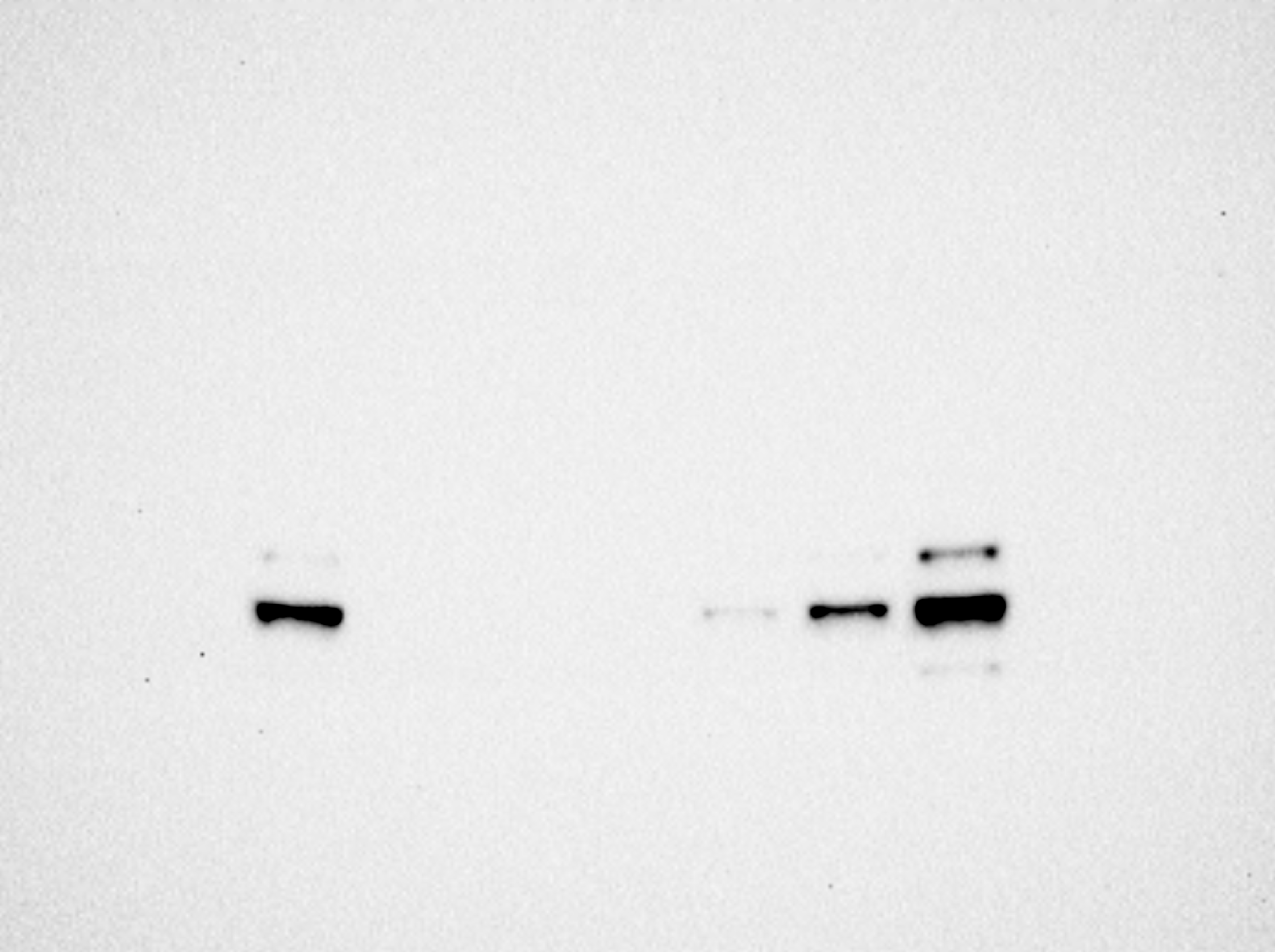

Supplement: Figure 5—source data 3. [file elife-80497-fig5-data3.zip › Figure 5-source data 3/Figure 5B/Figure 5 B - P(Thr389)-RPS6KB.tif]

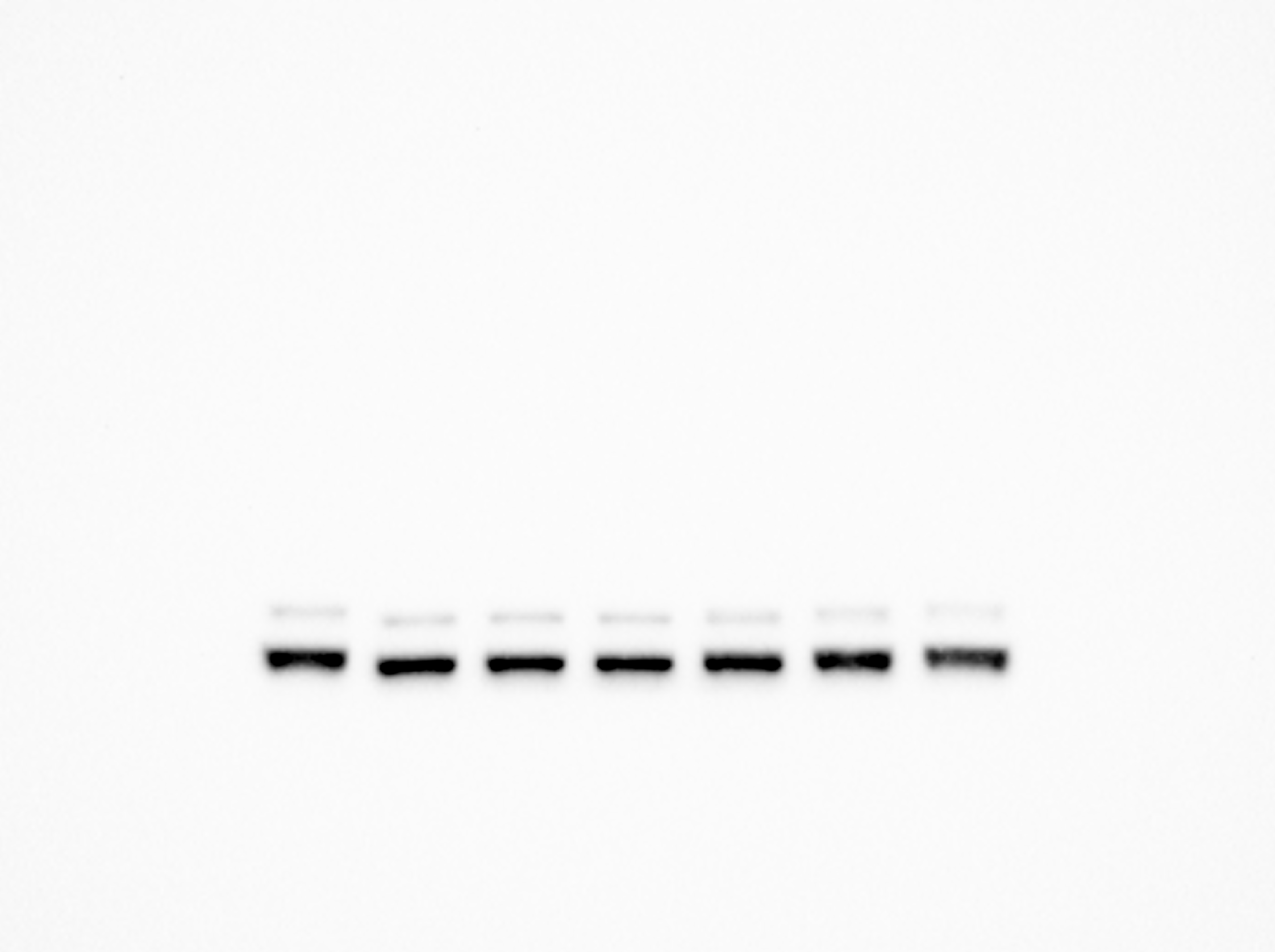

Supplement: Figure 5—source data 3. [file elife-80497-fig5-data3.zip › Figure 5-source data 3/Figure 5B/Figure 5 B - RPS6KB.tif]

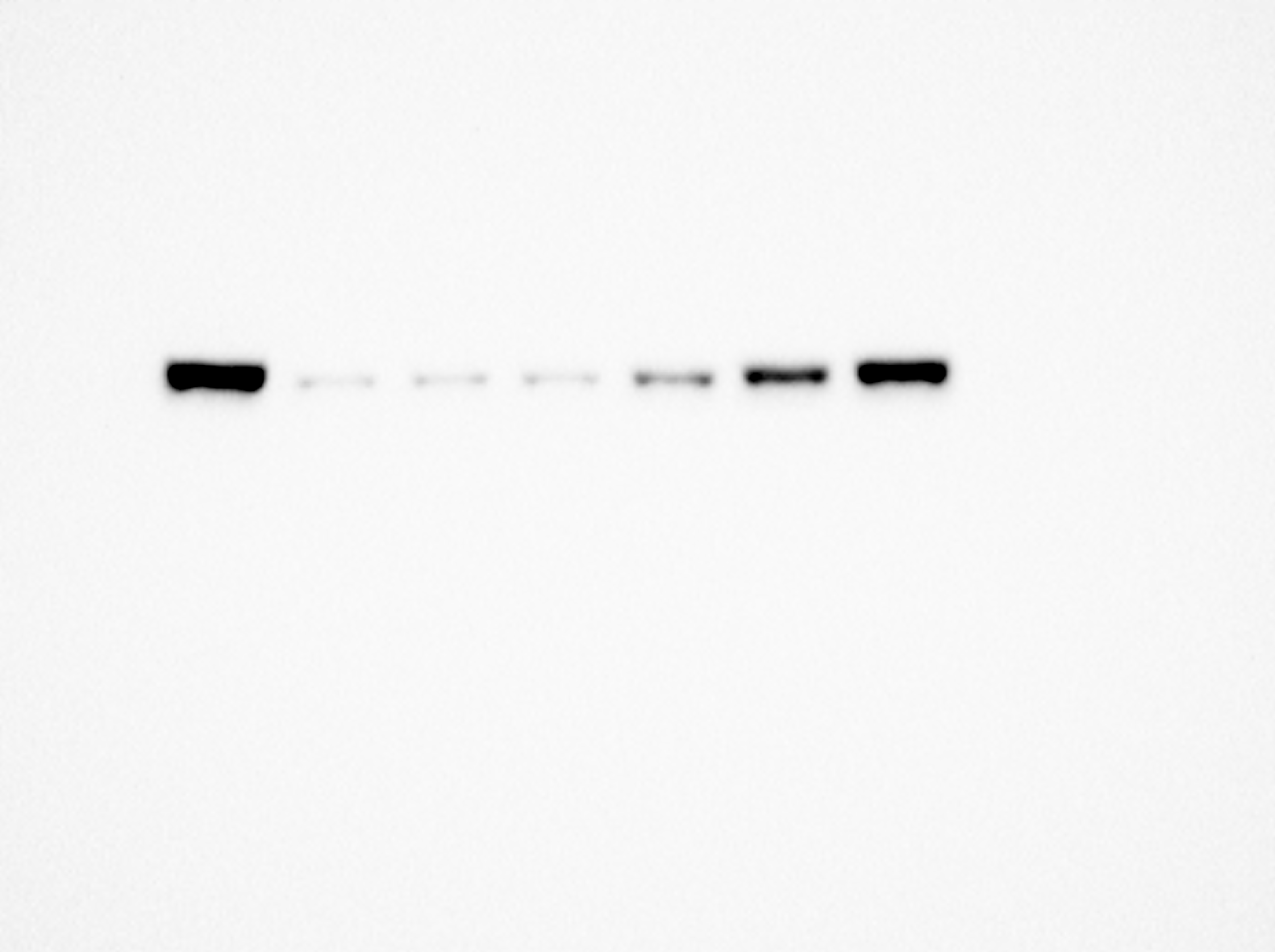

Supplement: Figure 5—source data 3. [file elife-80497-fig5-data3.zip › Figure 5-source data 3/Figure 5B/Figure 5 B - P(Ser235-236) -RPS6.tif]
