# Supplementary material for: Manganese is a physiologically relevant TORC1 activator in yeast and mammals
Source: eLife. 2022 Jul 29;11:e80497. doi: 10.7554/eLife.80497 (PMC9337852; doi:10.7554/eLife.80497)

Figure 1-Figure Supplement 1C

Samples loaded:

WT *GFP-TOR1* 0, 0.5, 1, 2 h RAP treatment; *pmr1*Δ *GFP-TOR1* 0, 0.5, 1, 2 h RAP treatment; *bsd2*Δ *GFP-TOR1* 0, 0.5, 1, 2 h RAP treatment; *pmr1*Δ *bsd2*Δ *GFP-TOR1* 0, 0.5, 1, 2 h RAP treatment.

Samples highlighted in blue are those shown in Figure 1-Figure supplement 1.

anti-GFP

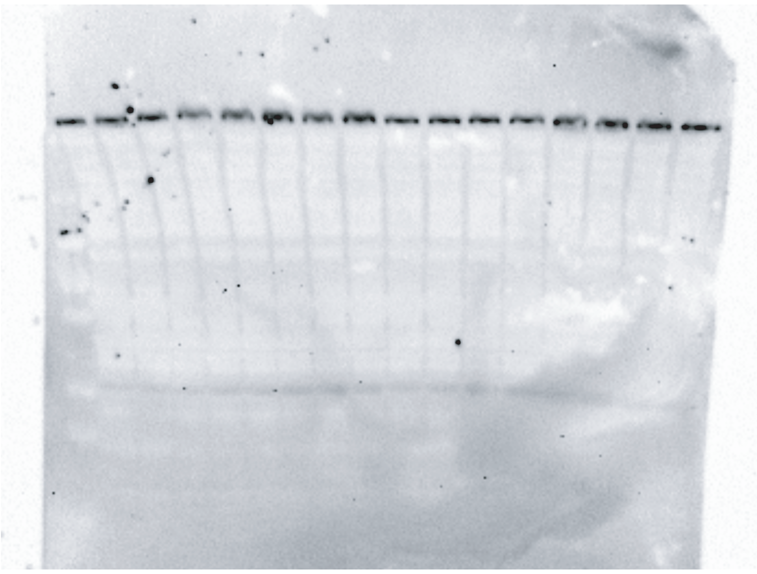

anti-G-6-PDH

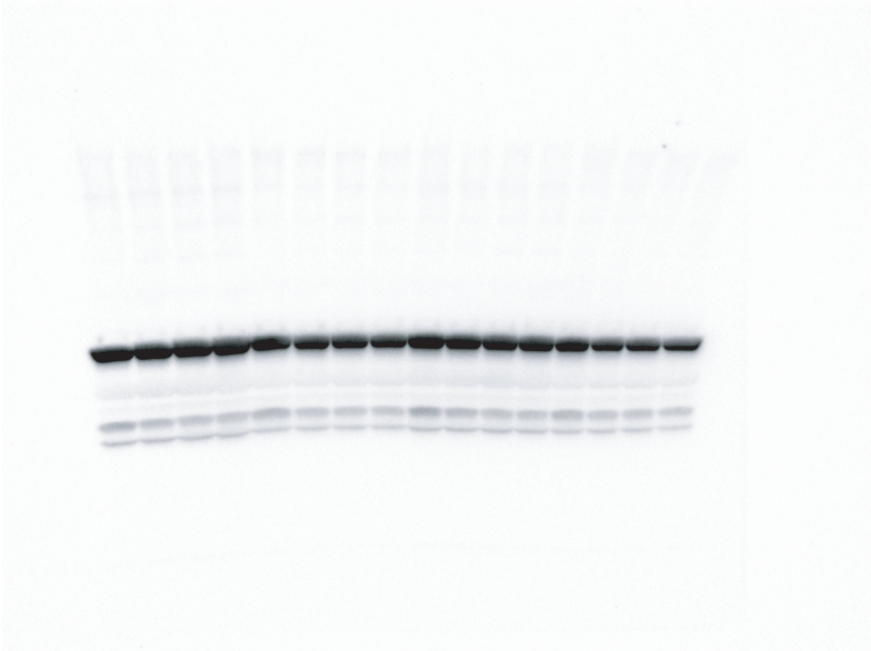

Supplement: Figure 1—figure supplement 1—source data 1. [file elife-80497-fig1-figsupp1-data1.pdf]
