# Supplementary material for: Manganese is a physiologically relevant TORC1 activator in yeast and mammals
Source: eLife. 2022 Jul 29;11:e80497. doi: 10.7554/eLife.80497 (PMC9337852; doi:10.7554/eLife.80497)

Figure 2A and 2C

EXPT1-Atg8-Om45
Samples loaded:
WT *ATG8-GFP* 0, 1, 2, 4 and 6h; *pmr1*Δ *ATG8-GFP* 0, 1, 2, 4 and 6h; blank; WT *GFP-OM45* 0, 1, 2, 4 and 6h; *pmr1*Δ *GFP-OM45* 0, 1, 2, 4 and 6h.

anti-GFP

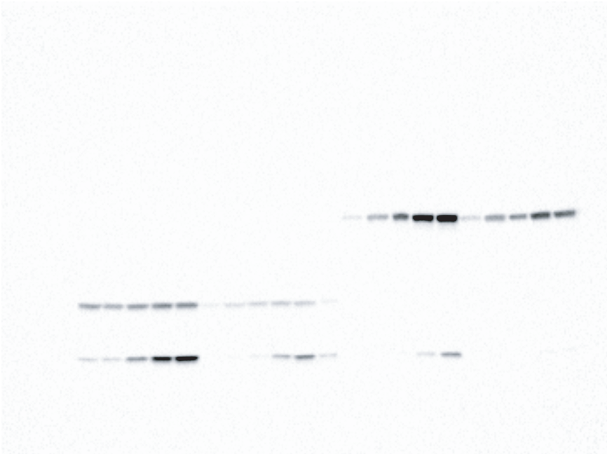

anti-G-6-PDH

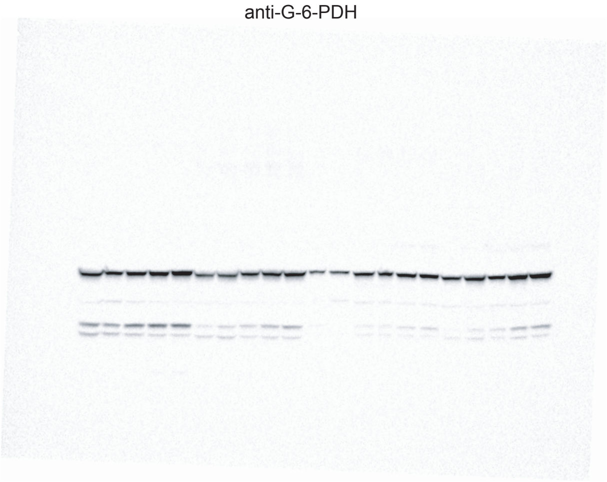

EXPT2-Atg8
Samples loaded:
WT *ATG8-GFP* 0, 1, 2, 4 and 6h; *pmr1*Δ *ATG8-GFP* 0, 1, 2, 4 and 6h; *smf2*Δ *ATG8-GFP* 0, 1, 2, 4 and 6h; *pmr1*Δ *smf2*Δ *ATG8-GFP* 0, 1, 2, 4 and 6h.

anti-GFP

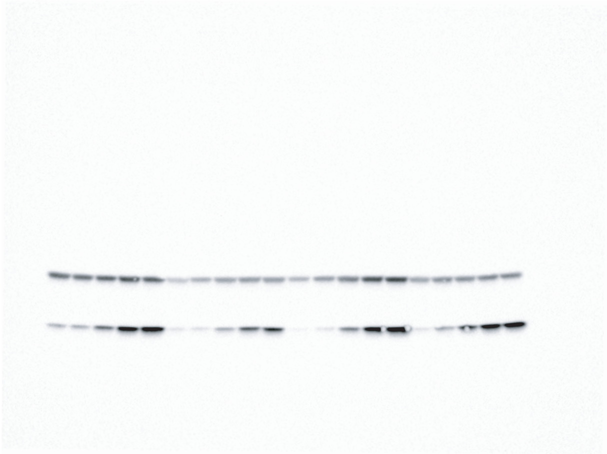

anti-G-6-PDH

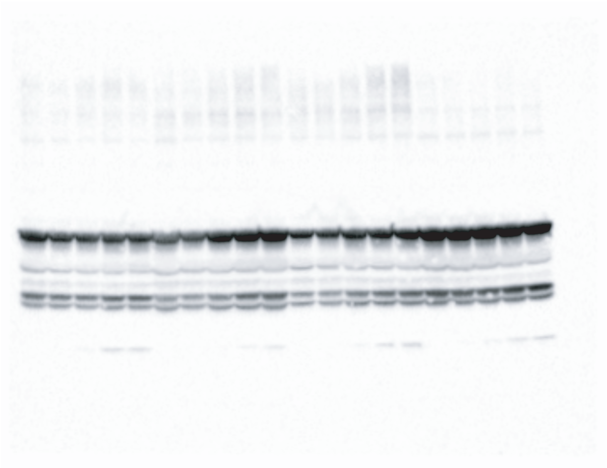

EXPT2-Om45
Samples loaded:
WT *GFP-OM45* 0, 1, 2, 4 and 6h; *pmr1*Δ *GFP-OM45* 0, 1, 2, 4 and 6h; *smf2*Δ *GFP-OM45* 0, 1, 2, 4 and 6h; *pmr1*Δ *smf2*Δ *GFP-OM45* 0, 1, 2, 4 and 6h.

anti-GFP

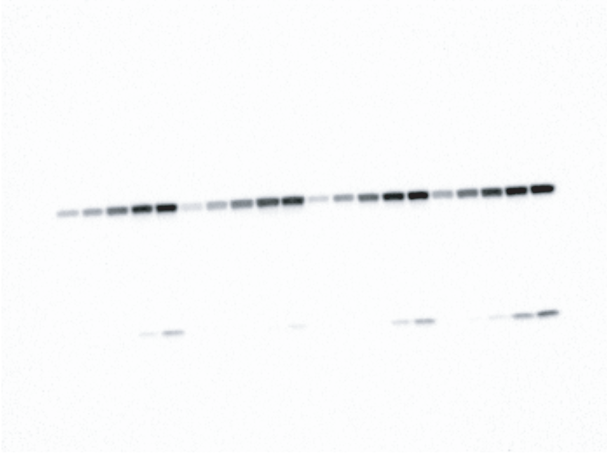

anti-G-6-PDH

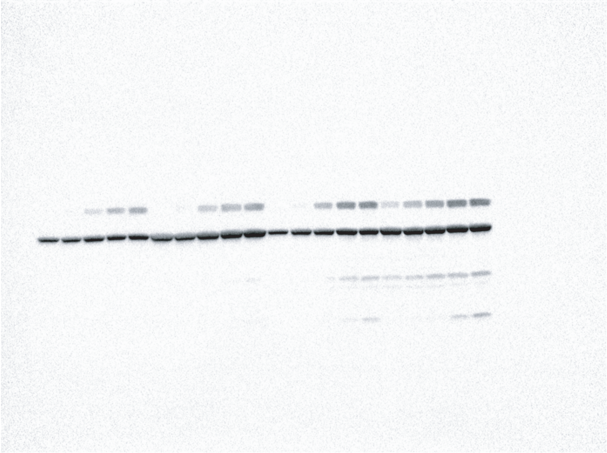

EXPT3-Atg8

Samples loaded:

WT *ATG8-GFP* 0, 2, 4 and 6h; *pmr1*Δ *ATG8-GFP* 0, 2, 4 and 6h; *smf2*Δ *ATG8-GFP* 0, 2, 4 and 6h; *pmr1*Δ *smf2*Δ *ATG8-GFP* 0, 2, 4 and 6h;
*smf2*Δ *ATG8-GFP bis* 0, 2, 4 and 6h; *pmr1*Δ *smf2*Δ *ATG8-GFP bis* 0, 2, 4 and 6h.

The samples highlighted in blue are those shown in Figure 2A.

anti-GFP, right part of blot

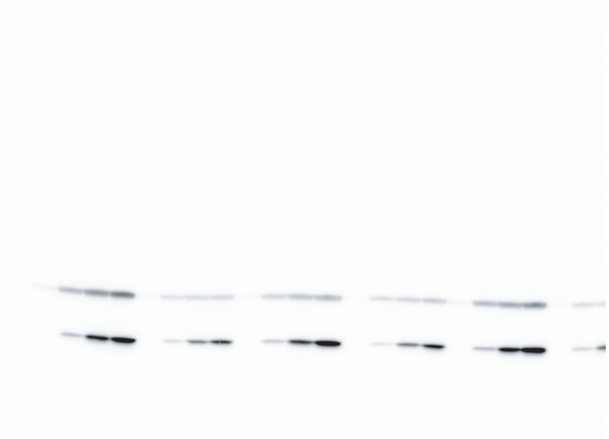

anti-GFP, left part of blot

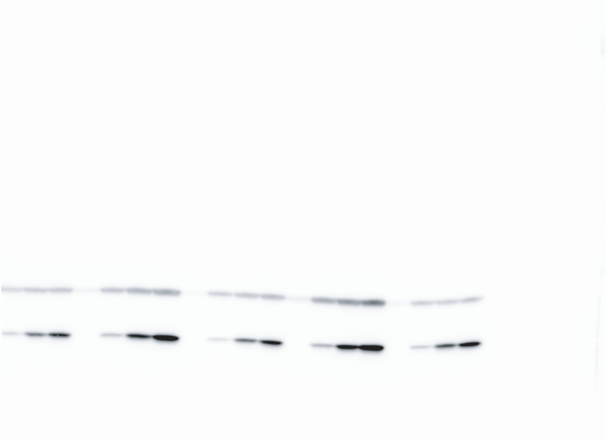

anti-G-6-PDH, right part of blot

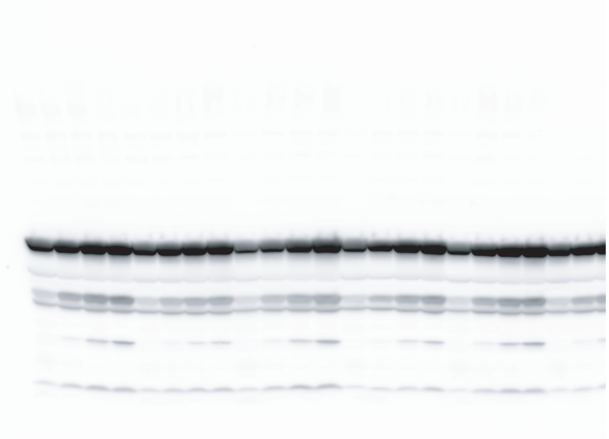

anti-G-6-PDH, left part of blot

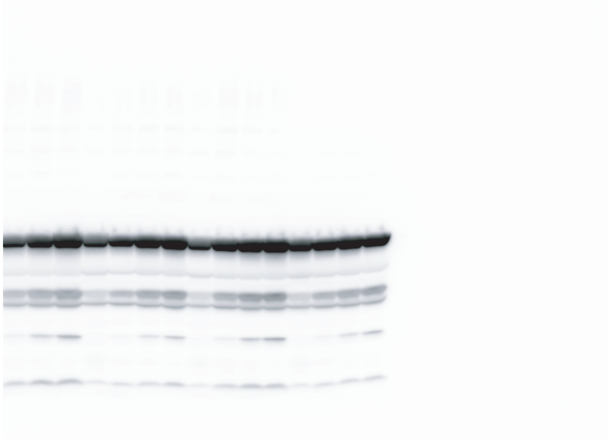

EXPT3-Om45

Samples loaded:

WT *GFP-OM45* 0, 2, 4 and 6h; *pmr1*Δ *GFP-OM45* 0, 2, 4 and 6h; *smf2*Δ *GFP-OM45* 0, 2, 4 and 6h; *pmr1*Δ *smf2*Δ *GFP-OM45* 0, 2, 4 and 6h; blank;
*smf2*Δ *GFP-OM45 bis* 0, 2, 4 and 6h; *pmr1*Δ *smf2*Δ *GFP-OM45 bis* 0, 2, 4 and 6h.

The samples highlighted in blue are those shown in Figure 2C.

anti-GFP, right part of blot

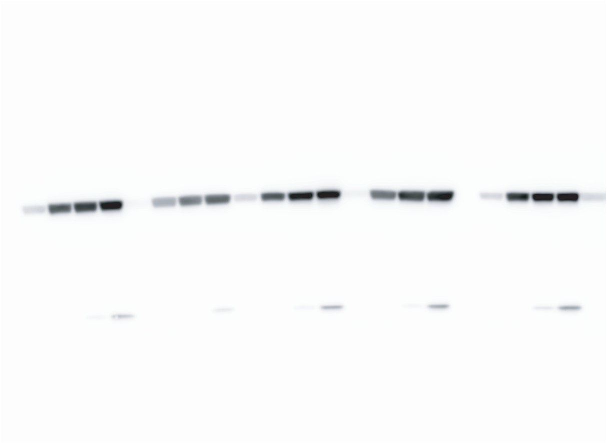

anti-GFP, left part of blot

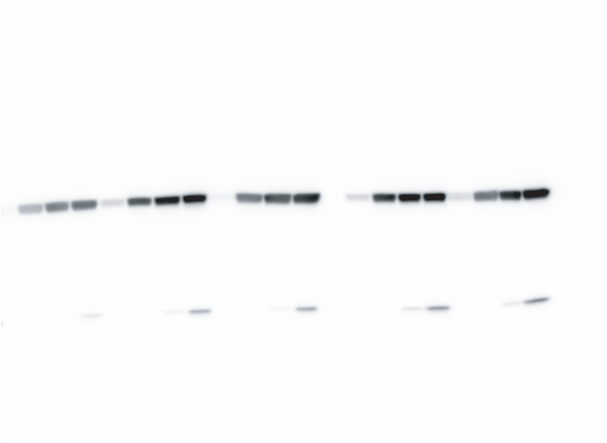

anti-G-6-PDH, right part of blot

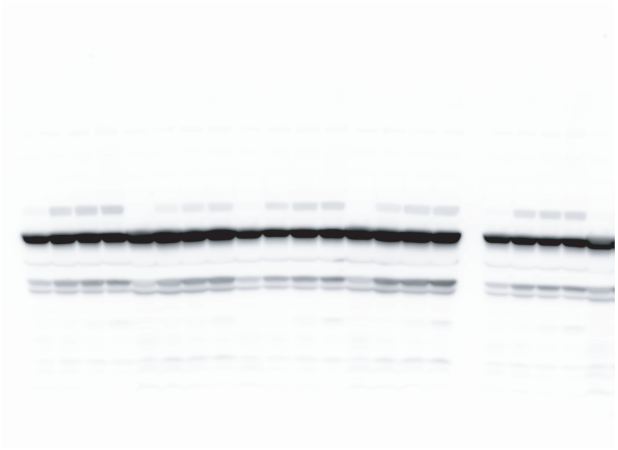

anti-G-6-PDH, left part of blot

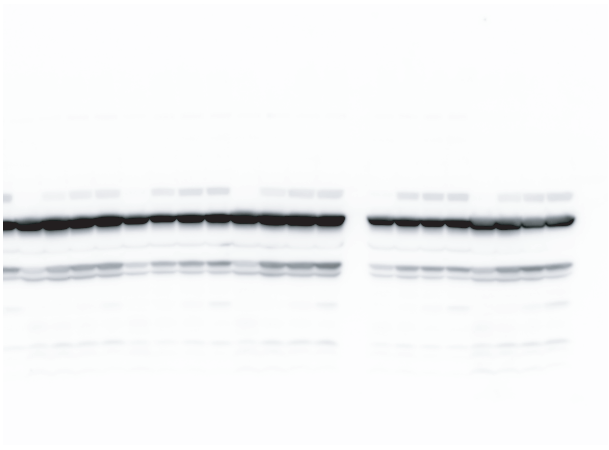

EXPT4-Atg8

Samples loaded:

WT *ATG8-GFP* 0, 1, 2, 4 and 6h; *pmr1*Δ *ATG8-GFP* 0, 1, 2, 4 and 6h; *smf2*Δ *ATG8-GFP* 0, 1, 2, 4 and 6h; *pmr1*Δ *smf2*Δ *ATG8-GFP* 0, 1, 2, 4 and 6h.

anti-GFP

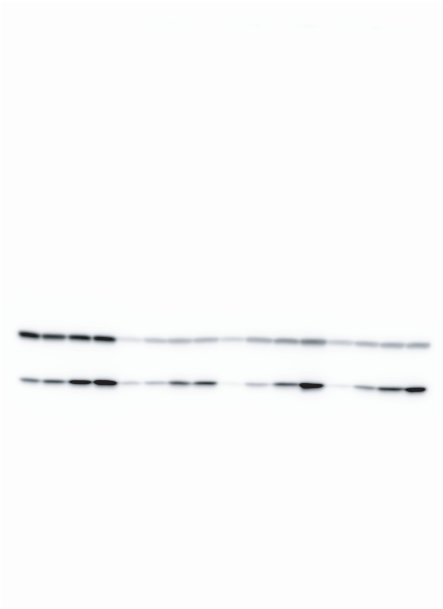

anti-G-6-PDH

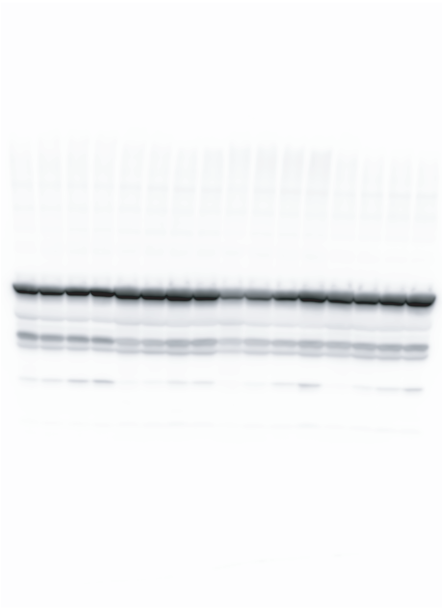

Figure 2F

EXPT1-EXPT2
Samples loaded:
WT *RTG3-GFP* 0, 15, and 30 min; *pmr1Δ RTG3-GFP* 0, 15, and 30 min; *smf2Δ RTG3-GFP* 0, 15, and 30 min; *pmr1Δ smf2Δ RTG3-GFP* 0, 15, and 30 min (exp1)
WT *RTG3-GFP* 0, 15, and 30 min; *pmr1Δ RTG3-GFP* 0, 15, and 30 min; *smf2Δ RTG3-GFP* 0, 15, and 30 min; *pmr1Δ smf2Δ RTG3-GFP* 0, 15, and 30 min (exp2)
The samples highlighted in blue are those shown in Figure 2F.

anti-GFP, left part of blot

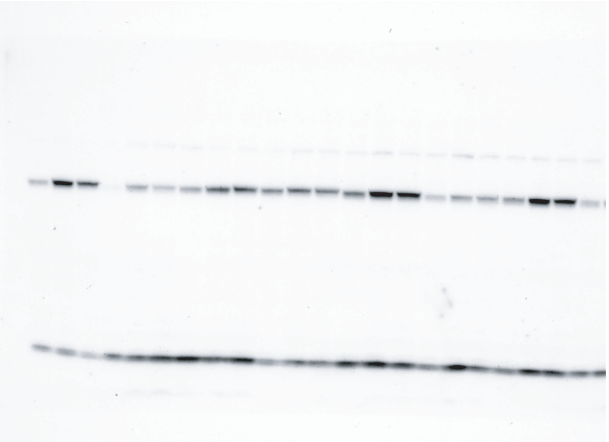

anti-GFP, right part of blot

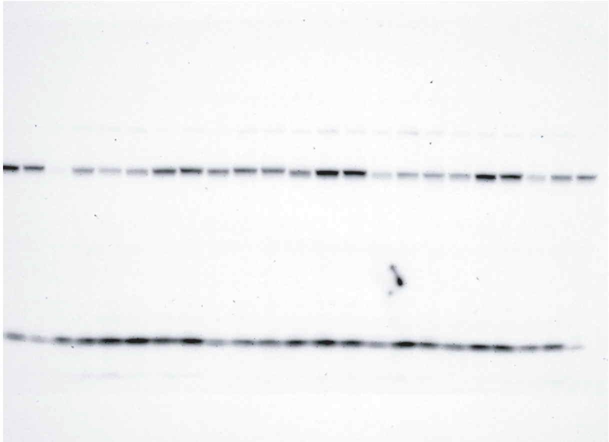

anti-G-6-PDH, left part of blot

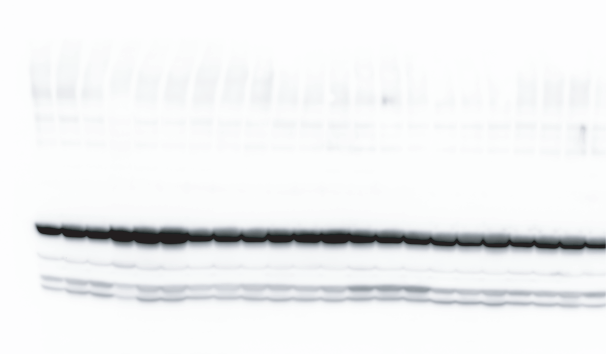

anti-G-6-PDH, right part of blot

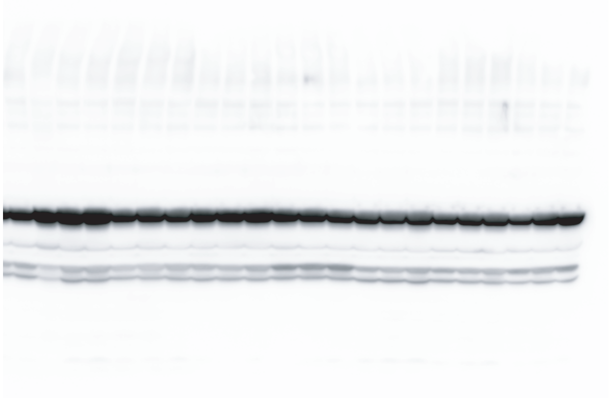

EXPT3
Samples loaded:
WT *RTG3-GFP* 0, 15, and 30 min; *pmr1Δ RTG3-GFP* 0, 15, and 30 min; *smf2Δ RTG3-GFP* 0, 15, and 30 min; *pmr1Δ smf2Δ RTG3-GFP* 0, 15, and 30 min.

anti-GFP

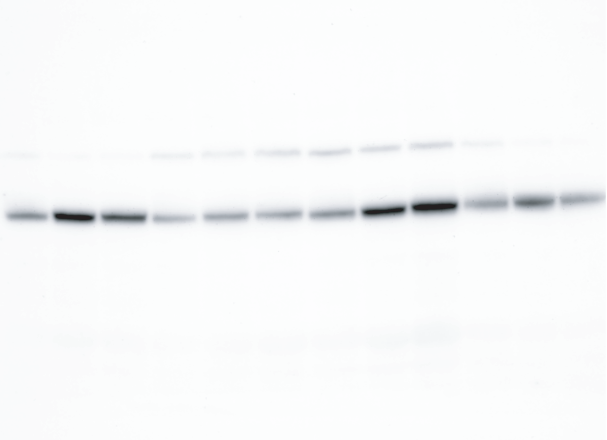

anti-G-6-PDH

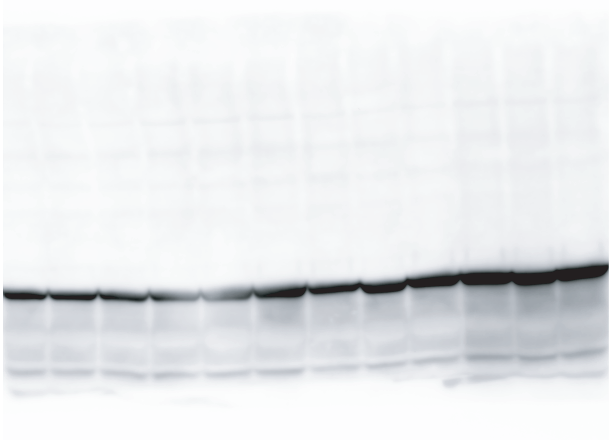

EXPT4
Samples loaded:
WT *RTG3-GFP* 0, 15, and 30 min; *pmr1Δ RTG3-GFP* 0, 15, and 30 min; *smf2Δ RTG3-GFP* 0, 15, and 30 min; *pmr1Δ smf2Δ RTG3-GFP* 0, 15, and 30 min.

anti-GFP

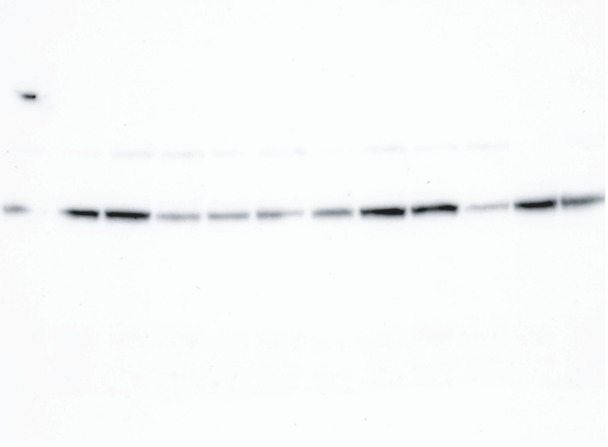

anti-G-6-PDH

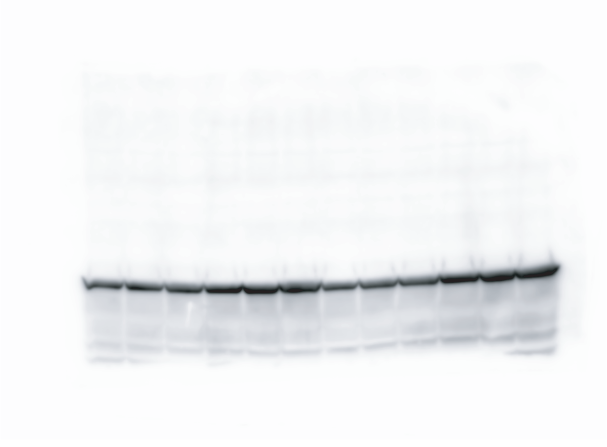

Supplement: Figure 2—source data 2. [file elife-80497-fig2-data2.pdf]
