# Supplementary material for: Manganese is a physiologically relevant TORC1 activator in yeast and mammals
Source: eLife. 2022 Jul 29;11:e80497. doi: 10.7554/eLife.80497 (PMC9337852; doi:10.7554/eLife.80497)

Figure 3A

Exp1.
Samples loaded:
MgCl2 (μM) MnCl2 (μM)
640 320 160 80 40 20 640 320 160 80 40 20

Sypro Ruby Staining

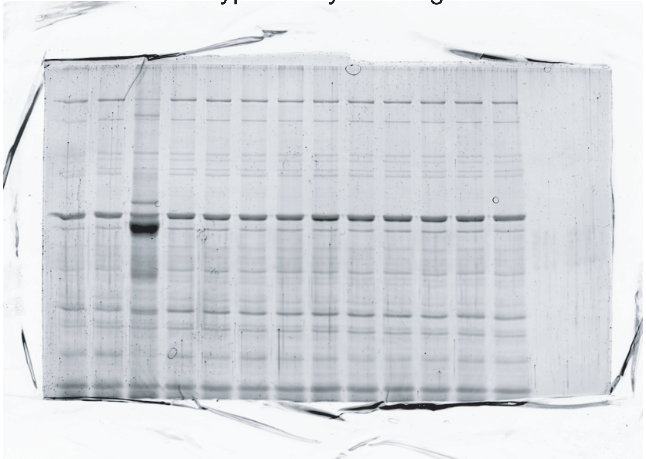

Autoradiography

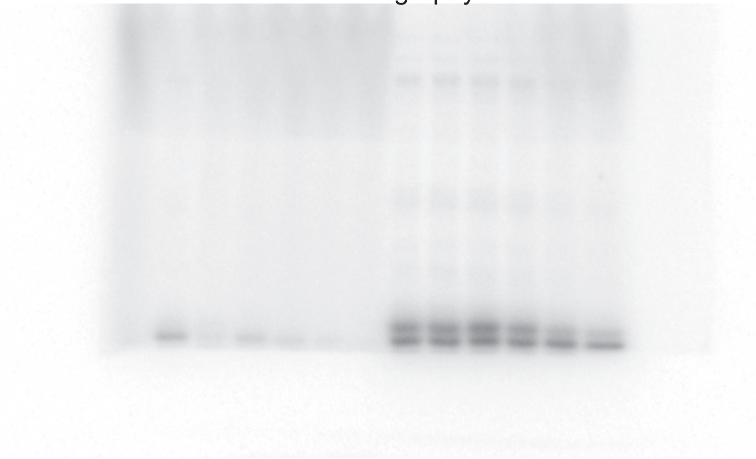

Exp2. Data shown in Figure 3A
Samples loaded:
MgCl2 (μM) MnCl2 (μM)
640 320 160 80 40 20 640 320 160 80 40 20

Sypro Ruby Staining

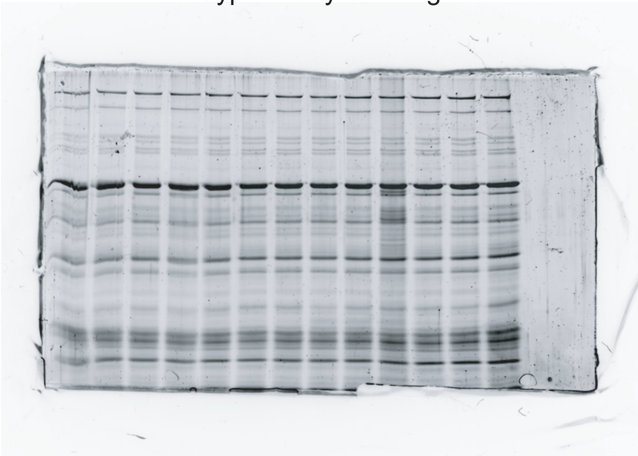

Autoradiography

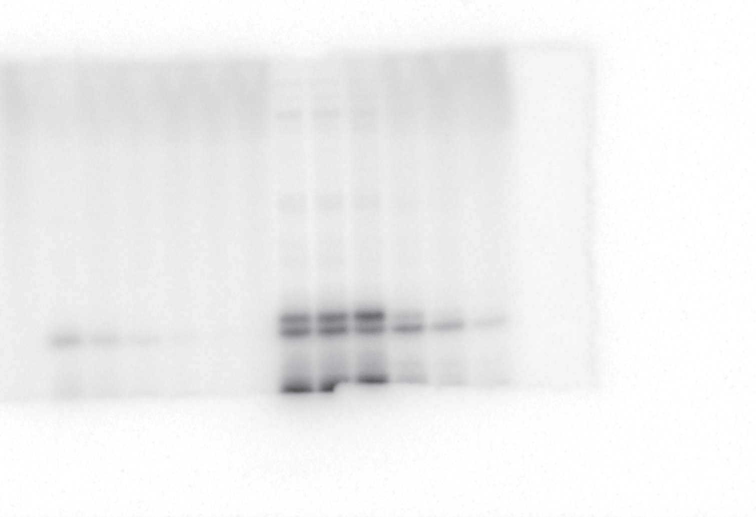

Exp3.
Samples loaded:
MgCl2 (μM) MnCl2 (μM)
20 40 80 160 320 640 20 40 80 160 320 640

Sypro Ruby Staining

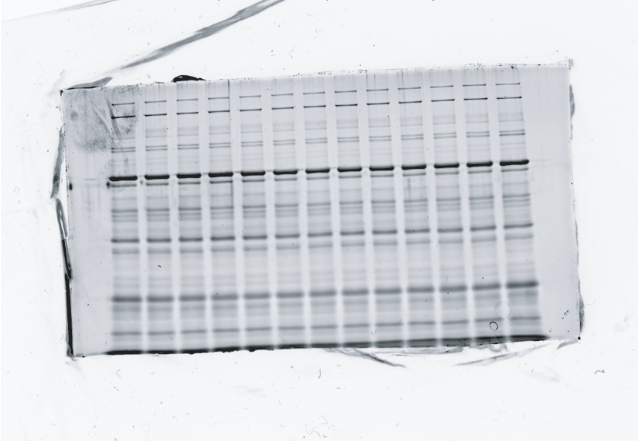

Autoradiography

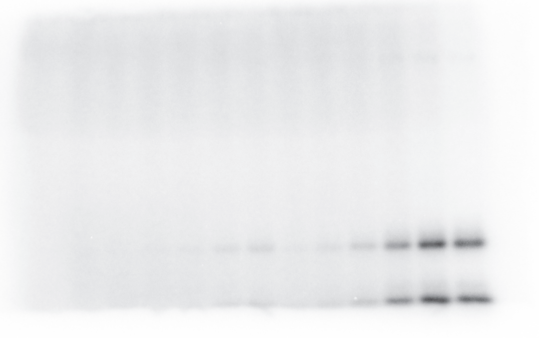

Figure 3C

Exp1. Data shown in Figure 3C.
Samples loaded:
MgCl2 4 mM, ATP (µM) MnCl2 0.16 mM, ATP (µM) MgCl2 4 mM, MnCl2 0.16 mM, ATP (µM)
1,2 3,6 11 33 100 1,2 3,6 11 33 100 1,2 3,6 11 33 100

Sypro Ruby Staining

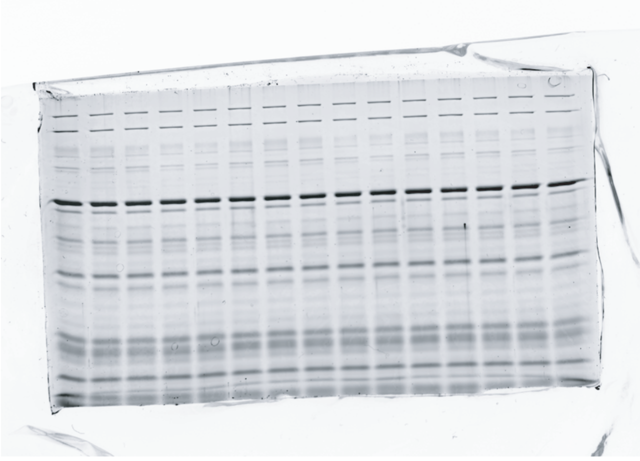

Autoradiography

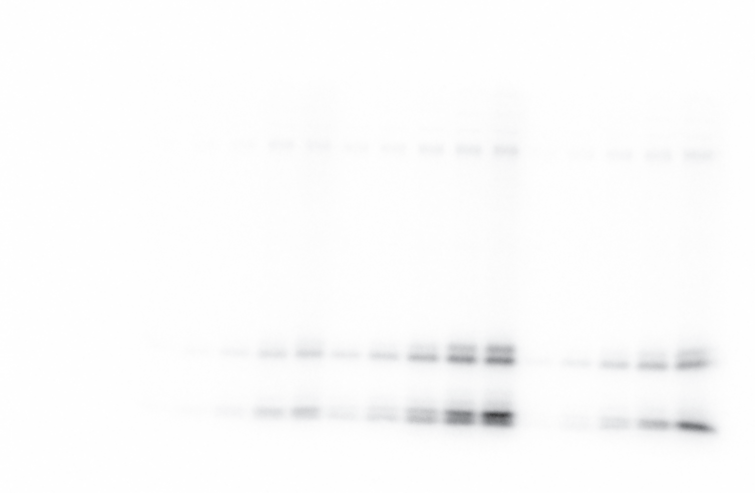

Exp2.
Samples loaded:
MgCl2 4 mM, ATP (µM) MnCl2 0.16 mM, ATP (µM) MgCl2 4 mM, MnCl2 0.16 mM, ATP (µM)
1,2 3,6 11 33 100 1,2 3,6 11 33 100 1,2 3,6 11 33 100

Sypro Ruby Staining

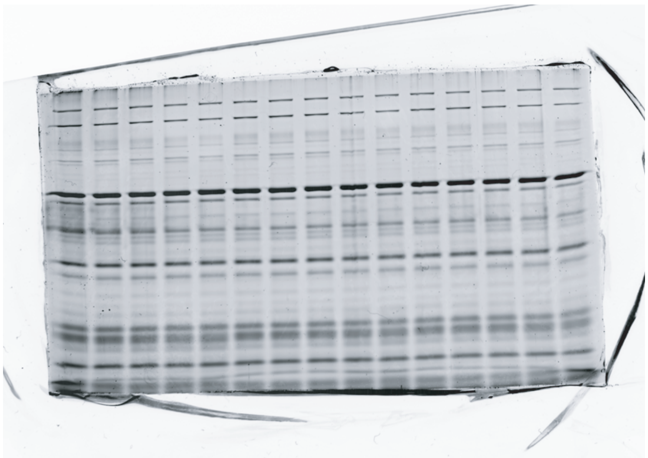

Autoradiography

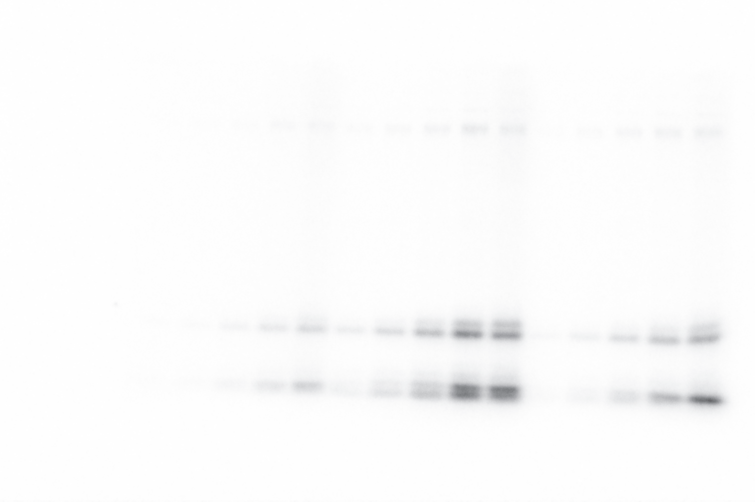

Exp3.
Samples loaded:
MgCl2 4 mM, MnCl2 0.16 mM, ATP (µM)MnCl2 0.16 mM, ATP (µM) MgCl2 4 mM, ATP (µM)
1,2 3,6 11 33 100 1,2 3,6 11 33 100 1,2 3,6 11 33 100

Sypro Ruby Staining

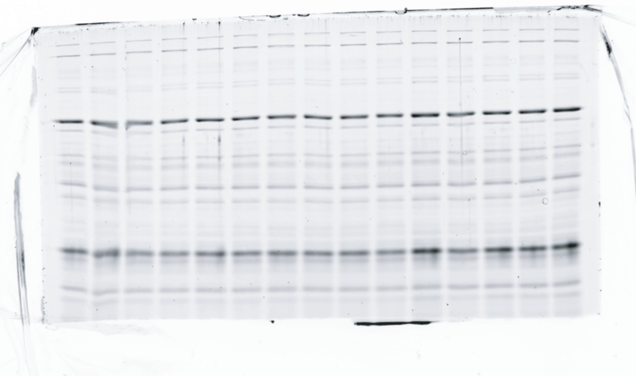

Autoradiography

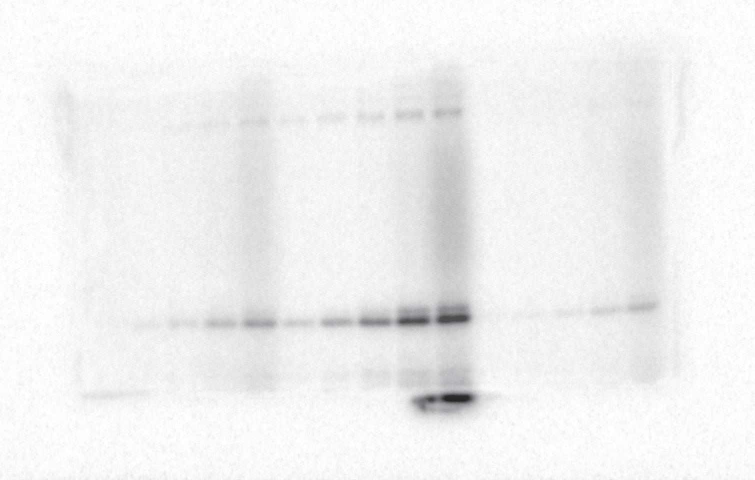

Expt4.

Samples loaded:

MgCl2 4 mM, ATP (µM) MnCl2 4 mM, ATP (µM)

1,2 3,6 11 33 100 300 600 1,2 3,6 11 33 100 300 600

The 300 and 600 µM samples were not quantified.

Sypro Ruby Staining

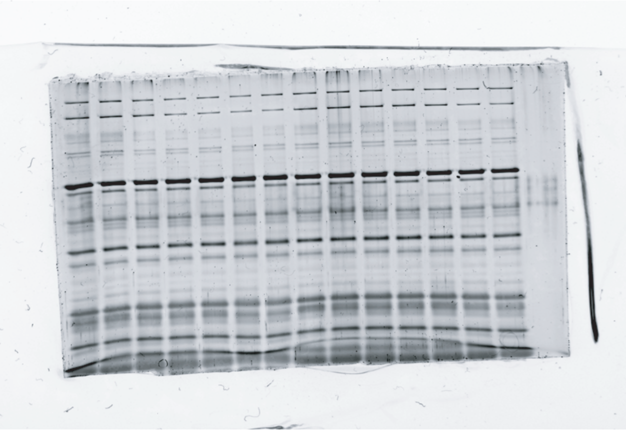

Autoradiography

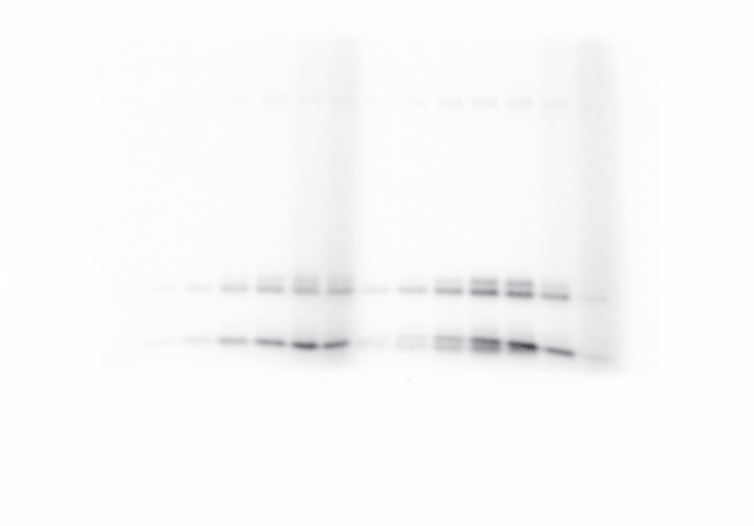

Supplement: Figure 3—source data 2. [file elife-80497-fig3-data2.pdf]
