# Supplementary material for: Manganese is a physiologically relevant TORC1 activator in yeast and mammals
Source: eLife. 2022 Jul 29;11:e80497. doi: 10.7554/eLife.80497 (PMC9337852; doi:10.7554/eLife.80497)

Figure 4A
EXPT1, 2, 3
Samples loaded:
0, 0.5, 1 and 2h RAP treatment; 0, 0.5, 1 and 2h CHX treatment; 0, 0.5, 1 and 2h RAP and CHX treatment; 0h no treatment
The blots shown in Figure 4A correspond to Expt 3.

anti-GFP

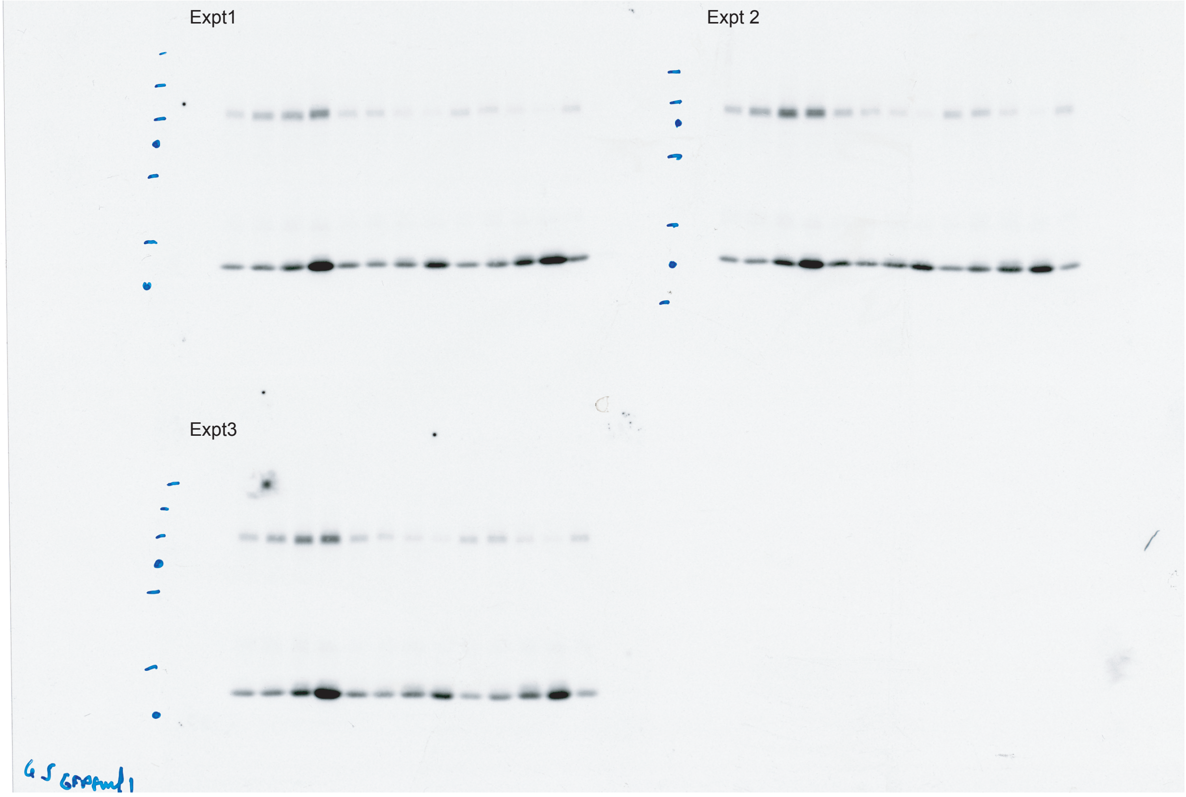

anti-Adh1

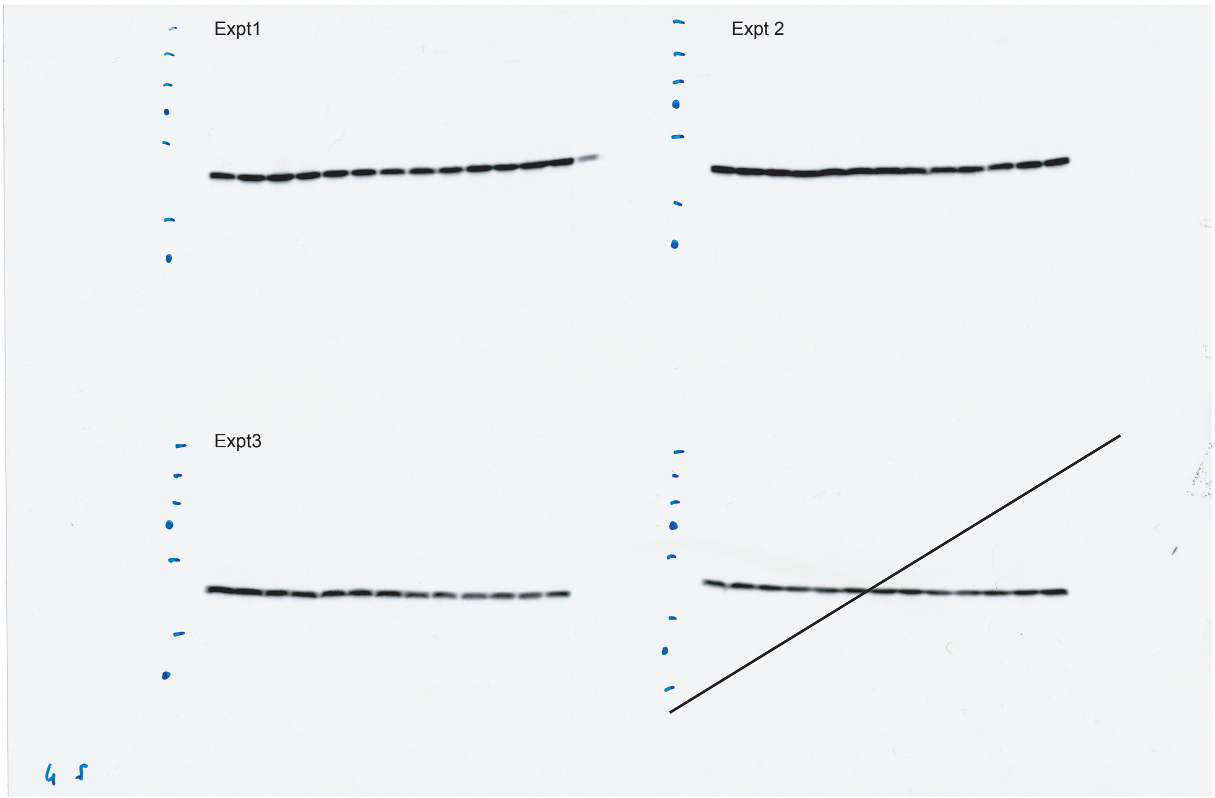

Figure 4D
EXPT1, 2, 3
Samples loaded:
0, 0.5, 1 and 2h RAP treatment; 0, 0.5, 1 and 2h CHX treatment; 0, 0.5, 1 and 2h RAP and CHX treatment; 0h no treatment
The blots shown in Figure 4D correspond to Expt 3.

anti-GFP

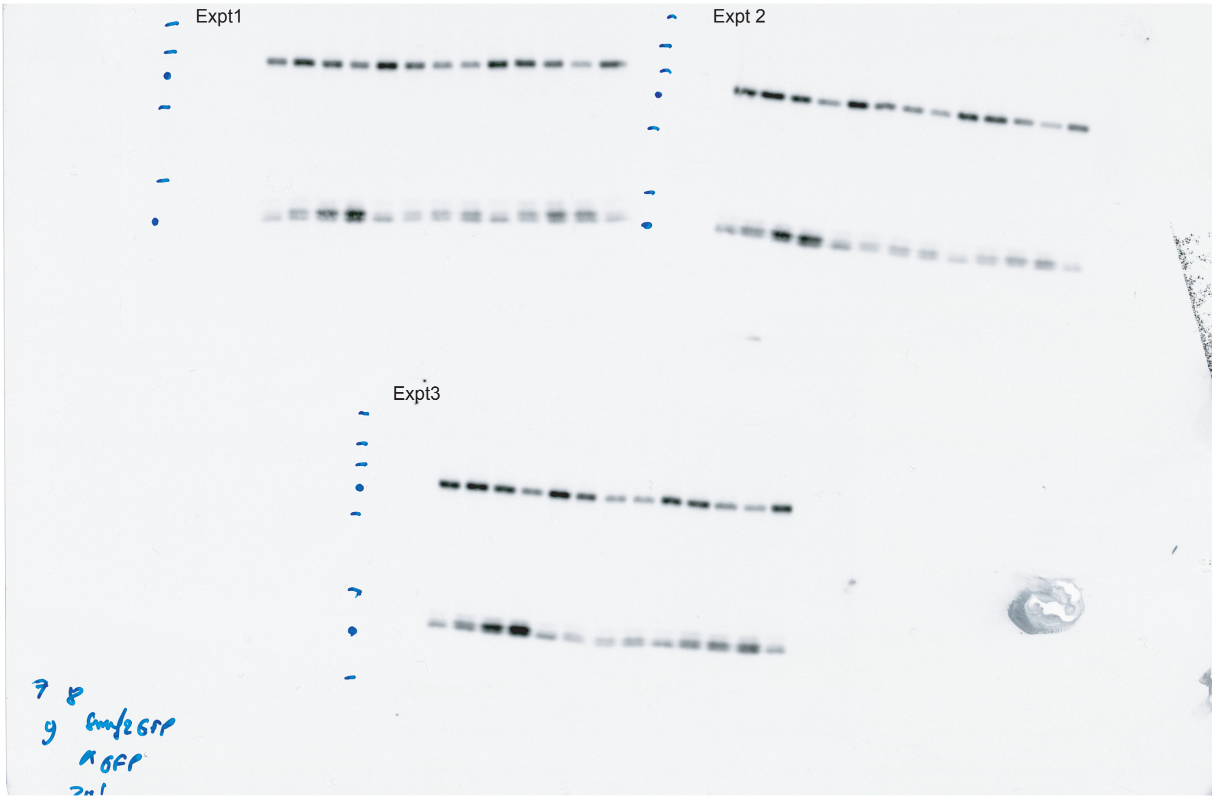

anti-Adh1

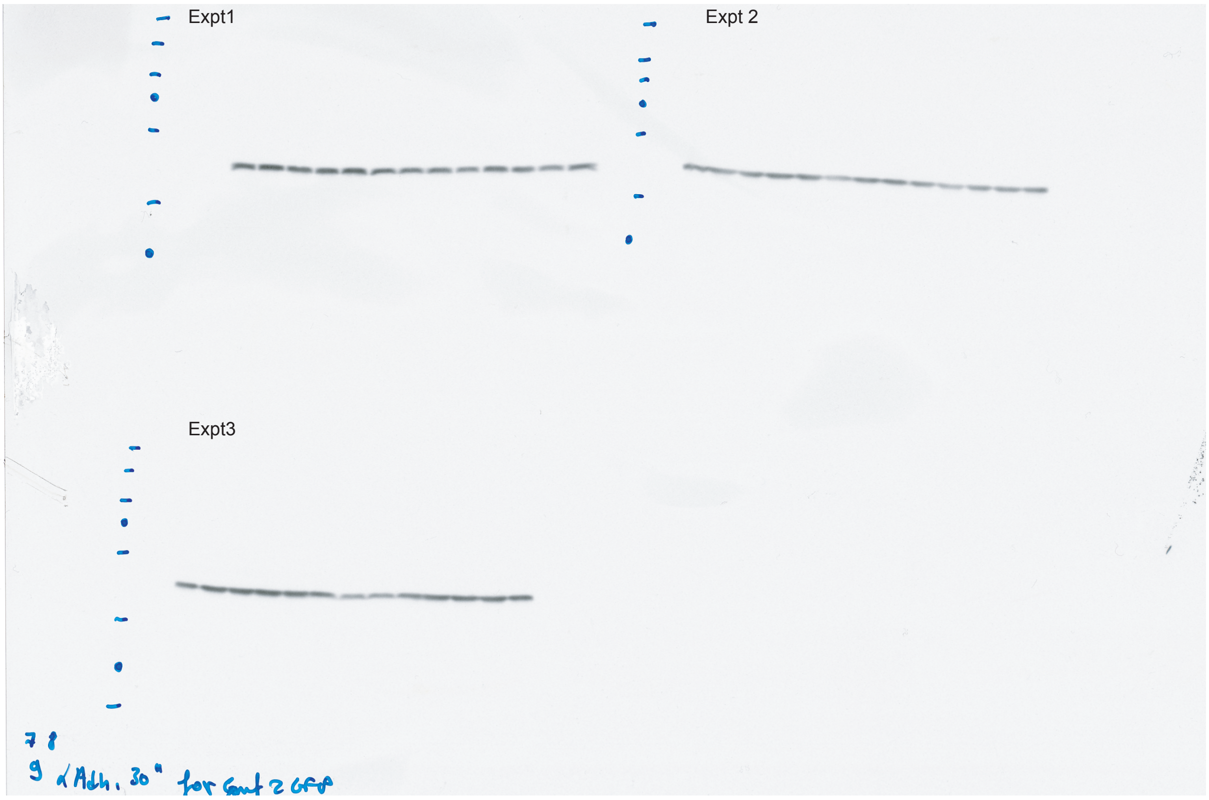

Supplement: Figure 4—source data 2. [file elife-80497-fig4-data2.pdf]
