# Supplementary material for: Manganese is a physiologically relevant TORC1 activator in yeast and mammals
Source: eLife. 2022 Jul 29;11:e80497. doi: 10.7554/eLife.80497 (PMC9337852; doi:10.7554/eLife.80497)

Figure 5A

Samples loaded:
U2OS: +AA; 0, 0.05, 0.1, 0.25, 0.5, 1 mM $MnCl_2$

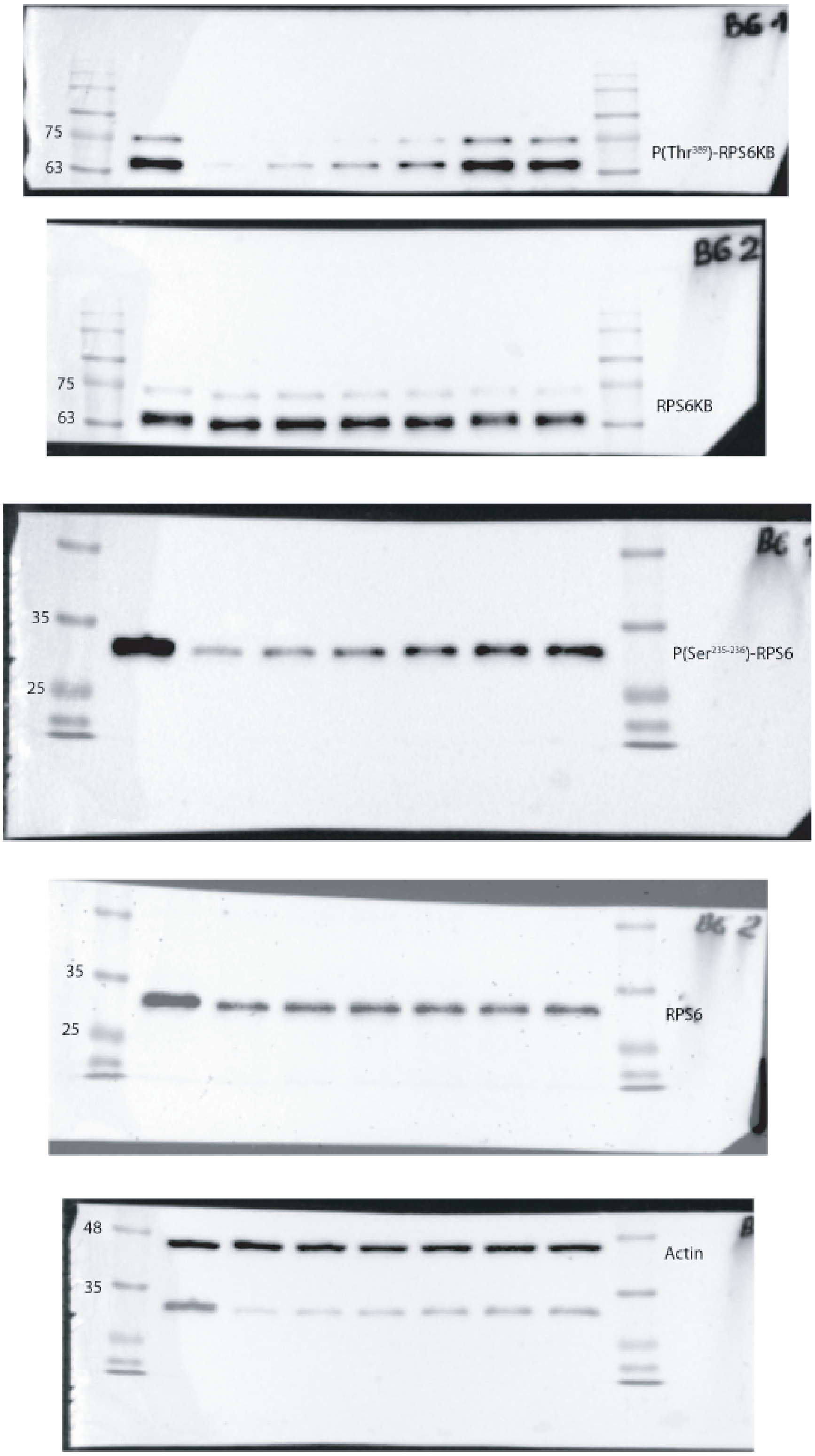

P(Thr^{389})-RPS6KB

RPS6KB

P(Ser$^{235-236}$)-RPS6

RPS6

Actin

Figure 5B

Samples loaded:
HEK293T: +AA; 0, 0.05, 0.1, 0.25, 0.5, 1 mM $MnCl_2$

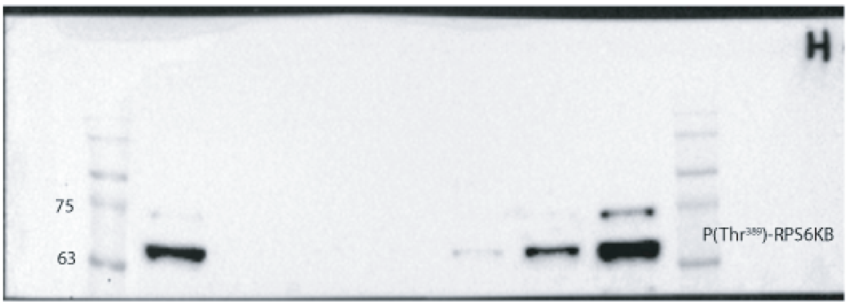

P(Thr^{389})-RPS6KB

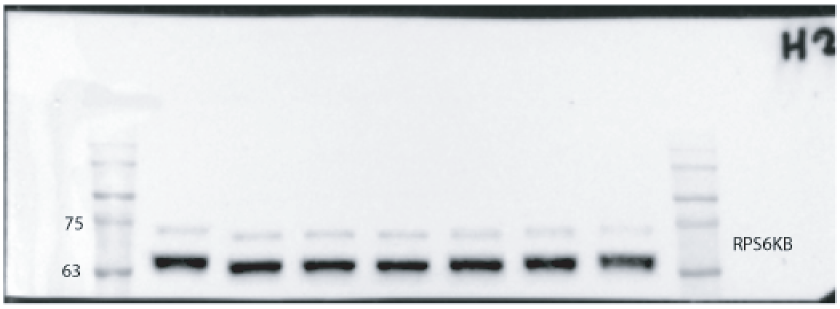

RPS6KB

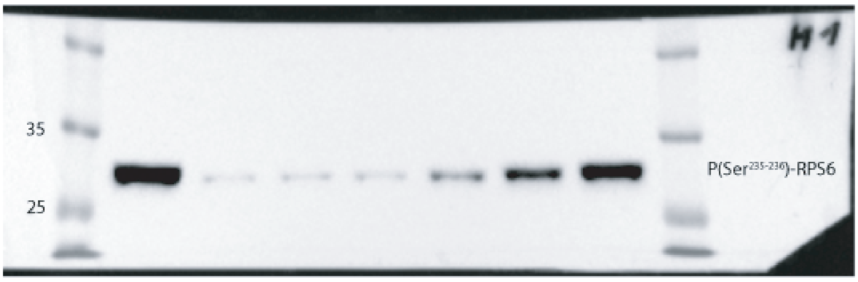

P(Ser$^{235\text{-}236}$)-RPS6

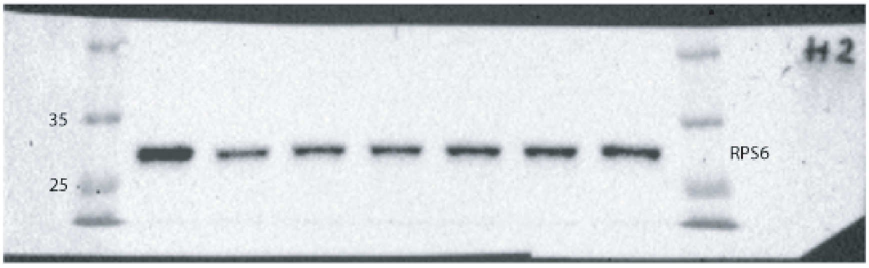

RPS6

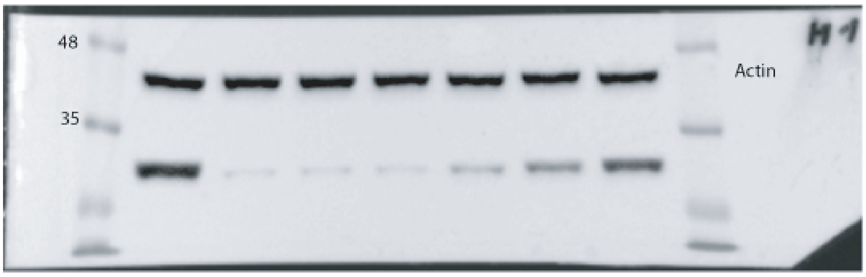

Actin

Figure 5C

Samples loaded:
U2OS: +AA -MnCl$_2$; -AA -MnCl$_2$; -AA +MnCl$_2$

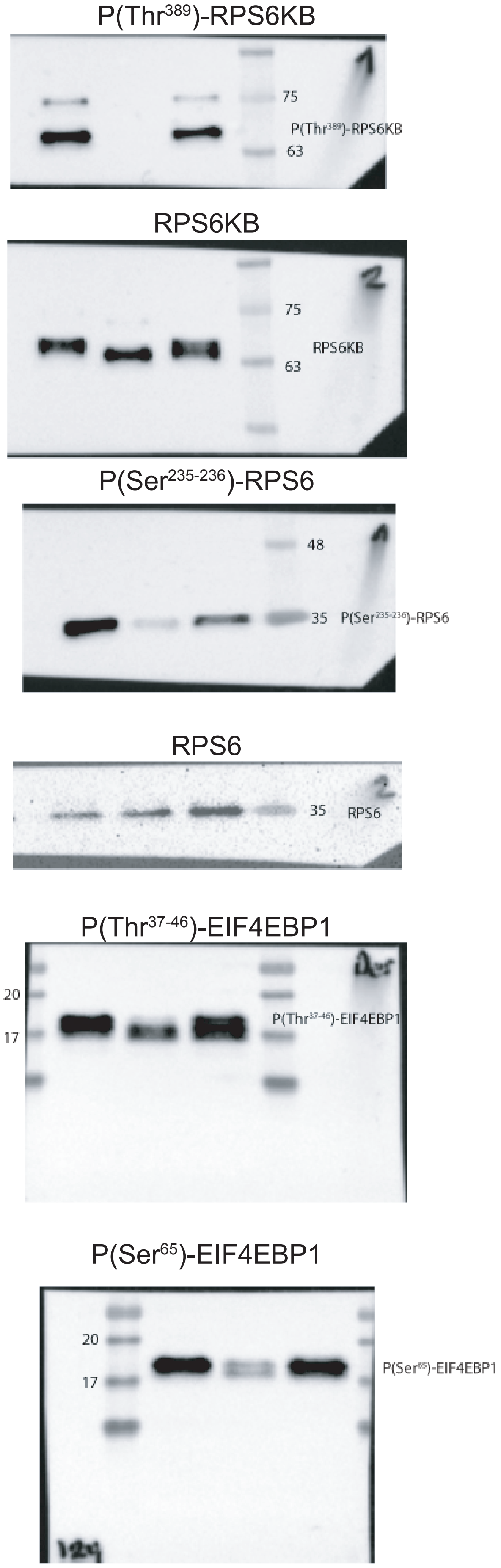

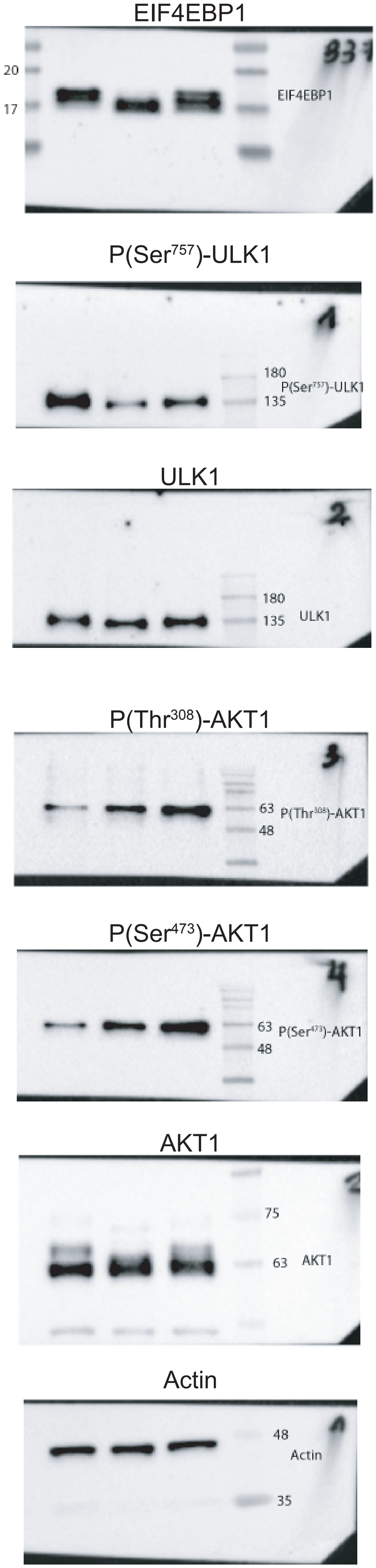

Figure 5D

Samples loaded:
U2OS: +AA -$MnCl_2$; -AA -$MnCl_2$; -AA +$MnCl_2$

MAP1LC3

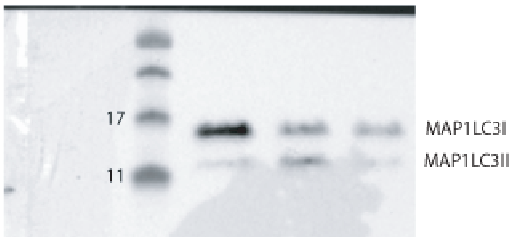

Actin

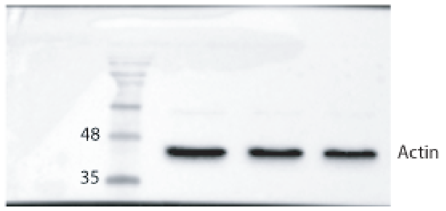

Figure 5E

Samples loaded:
U2OS: +AA -$MnCl_2$+CQ; -AA -$MnCl_2$+CQ; -AA +$MnCl_2$+CQ

MAP1LC3

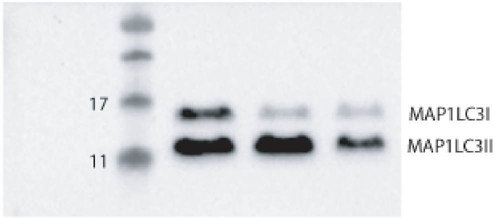

Actin

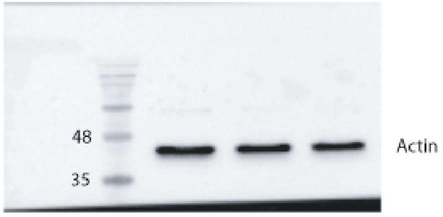

Supplement: Figure 5—source data 2. [file elife-80497-fig5-data2.pdf]
