# Supplementary material for: Manganese is a physiologically relevant TORC1 activator in yeast and mammals
Source: eLife. 2022 Jul 29;11:e80497. doi: 10.7554/eLife.80497 (PMC9337852; doi:10.7554/eLife.80497)
Supplement: Supplementary file 1. [file elife-80497-supp1.docx]

**Table 1.** RTG1-3 target genes down-regulated in *pmr1∆* cells.

| **Gene name** | **Fold change** |
| --- | --- |
| *BDH2* | -4,8 |
| *CIT2* | -1,65 |
| *FMP48* | -5,1 |
| *HSP12* | -4,75 |
| *HXT5* | -2,2 |
| *IDH1* | -1,85 |
| *IDH2* | -1,6 |
| *MSC1* | -2,75 |
| *PHM7* | -1,85 |
| *RTC3* | -2,2 |
| *SPS100* | -3,15 |
| *TKL2* | -3 |
| *YNL194C* | -8,4 |

Data from García-Rodríguez et al, 2012. GEO accession GSE29420
