# Supplementary material for: Manganese is a physiologically relevant TORC1 activator in yeast and mammals
Source: eLife. 2022 Jul 29;11:e80497. doi: 10.7554/eLife.80497 (PMC9337852; doi:10.7554/eLife.80497)
Supplement: Supplementary file 2. [file elife-80497-supp2.docx]

**Table 2.** Plasmids used in this study.

| **Plasmid** | **Relevant Genotype** | **Source** |
| --- | --- | --- |
| VGp160 | *GFP-ATG8 CEN URA3* | V. Goder |
| pSIVu | Cloning vector for single integration into the *URA3* locus | S. Pelet |
| pVA1458 | *SMF1pGFP-SMF1 URA3* | V. Albanese/S. Leon |
| p4301 | *SMF1pGFP-SMF1 Sac*I/*Kpn*I fragment from pVA1458 cloned into single integration plasmid pSIVu *URA3* | This study |
| p2GPD-SMF2 | *SMF2 URA3* | This study |
| p2GPD-Slc11a1 | *cSlc11a1 URA3* | This study |
| p2GPD-Slc11a2 | *cSlc11a2 URA3* | This study |
| pRS416-RTG3 | *RTG3p::RTG3-GFP URA3* | E. de Nadal |
| pRS416 | *URA3* | P. Hieter |
| p2GPD | *URA3* | K. D. Hirschi |
| pVCX1-M1 | *VXC1-M1 URA3* | K. D. Hirschi |
