# Supplementary material for: Manganese is a physiologically relevant TORC1 activator in yeast and mammals
Source: eLife. 2022 Jul 29;11:e80497. doi: 10.7554/eLife.80497 (PMC9337852; doi:10.7554/eLife.80497)
Supplement: Supplementary file 3. [file elife-80497-supp3.docx]

**Table 3.** Yeast strains used in this study.

| **Strains** | **Relevant Genotype** | **Source** |
| --- | --- | --- |
| BY4741 | *MATa his3Δ1 leu2Δ0 met15Δ0 ura3Δ0* | Euroscarf |
| YGL167C | *pmr1∆::KAN*, isogenic to BY4741 | Euroscarf |
| YBR290W | *bsd2∆::KAN*, isogenic to BY4741 | Euroscarf |
| RW187 | *pmr1∆::NAT bsd2∆::KAN*, isogenic to BY4741 | This study |
| YOL122C | *smf1∆::KAN*, isogenic to BY4741 | Euroscarf |
| NG222 | *pmr1∆::NAT smf1∆::KAN*, isogenic to BY4741 | R. Wellinger |
| YHR050W | *smf2∆::KAN*, isogenic to BY4741 | Euroscarf |
| NGY183 | *pmr1∆::NAT smf2∆::KAN*, isogenic to BY4741 | R. Wellinger |
| RWY188 | *bsd2∆::KAN smf2∆::NAT*, isogenic to BY4741 | This study |
| RKH395 | *MATa LEU2::GFP-TOR1 his3∆1 leu2∆0 ura3∆0* | C. De Virgilio |
| RWY128 | *pmr1∆::NAT,* isogenic to RKH395 | This study |
| YKR039W | *gap1∆::KAN*, isogenic to BY4741 | Euroscarf |
| RWY176 | *pmr1∆::NAT gap1∆::KAN*, isogenic to BY4741 | This study |
| RWY178 | *pmr1∆::NAT atx2∆::KAN*, isogenic to BY4741 | This study |
| RWY056 | *pmr1∆::NAT gdt1∆::KAN*, isogenic to BY4741 | This study |
| NGY232 | *pmr1∆::NAT spf1∆::KAN*, isogenic to BY4741 | This study |
| NGY190 | *pmr1∆::NAT ccc1∆::KAN*, isogenic to BY4741 | R. Wellinger |
| RWY 174 | *pmr1∆::NAT mtm1∆::KAN*, isogenic to BY4741 | This study |
| NGY223 | *pmr1∆::NAT pho84∆::KAN*, isogenic to BY4741 | R. Wellinger |
| SEY6210 | *MATα leu2-3,112 ura3-52 his3-∆200 trp-∆901 lys2-801 suc2-∆9 GAL* | C. Ungermann |
| CUY4517 | *OM45-GFP::HIS3*, isogenic to SEY6210 | C. Ungermann |
| RWY158 | *pmr1∆::KAN OM45-GFP::HIS3*, isogenic to SEY6210 | This study |
| RWY189 | *smf2∆::KAN OM45-GFP::HIS3*, isogenic to SEY6210 | This study |
| RWY190 | *pmr1∆::NAT smf2∆::KAN OM45-GFP::HIS3*, isogenic to SEY6210 | This study |
| NGY233 | *SMF2-GFP- CaURA3*, isogenic to BY4741 | R. Wellinger |
| MP6988 | *smf1∆::KAN URA3::SMF1p-GFP-SMF1*, isogenic to BY4741 | This study |
| MP6994 | *smf1∆::KAN URA3::SMF1p-GFP-SMF1 VPH1-mCherry::HIS3,* isogenic to BY4741 | This study |
| MP6998 | *SMF2-GFP- CaURA3 VPH1-mCherry::HIS3*, isogenic to BY4741 | This study |
